# Supplementary material for: Ion binding with charge inversion combined with screening modulates DEAD box helicase phase transitions
Source: Cell Rep. 2023 Nov 18;42(11):113375. doi: 10.1016/j.celrep.2023.113375 (PMC10935546; doi:10.1016/j.celrep.2023.113375)
Supplement: Document S1. Figures S1‒S5, Tables S1‒S3, and supplemental references [file mmc1.pdf]

Cell Reports, Volume *42*

Supplemental information

Ion binding with charge inversion combined with screening modulates DEAD box helicase phase transitions

Michael D. Crabtree, Jack Holland, Arvind S. Pillai, Purnima S. Kompella, Leon Babl, Noah N. Turner, James T. Eaton, Georg K.A. Hochberg, Dirk G.A.L. Aarts, Christina Redfield, Andrew J. Baldwin, and Timothy J. Nott

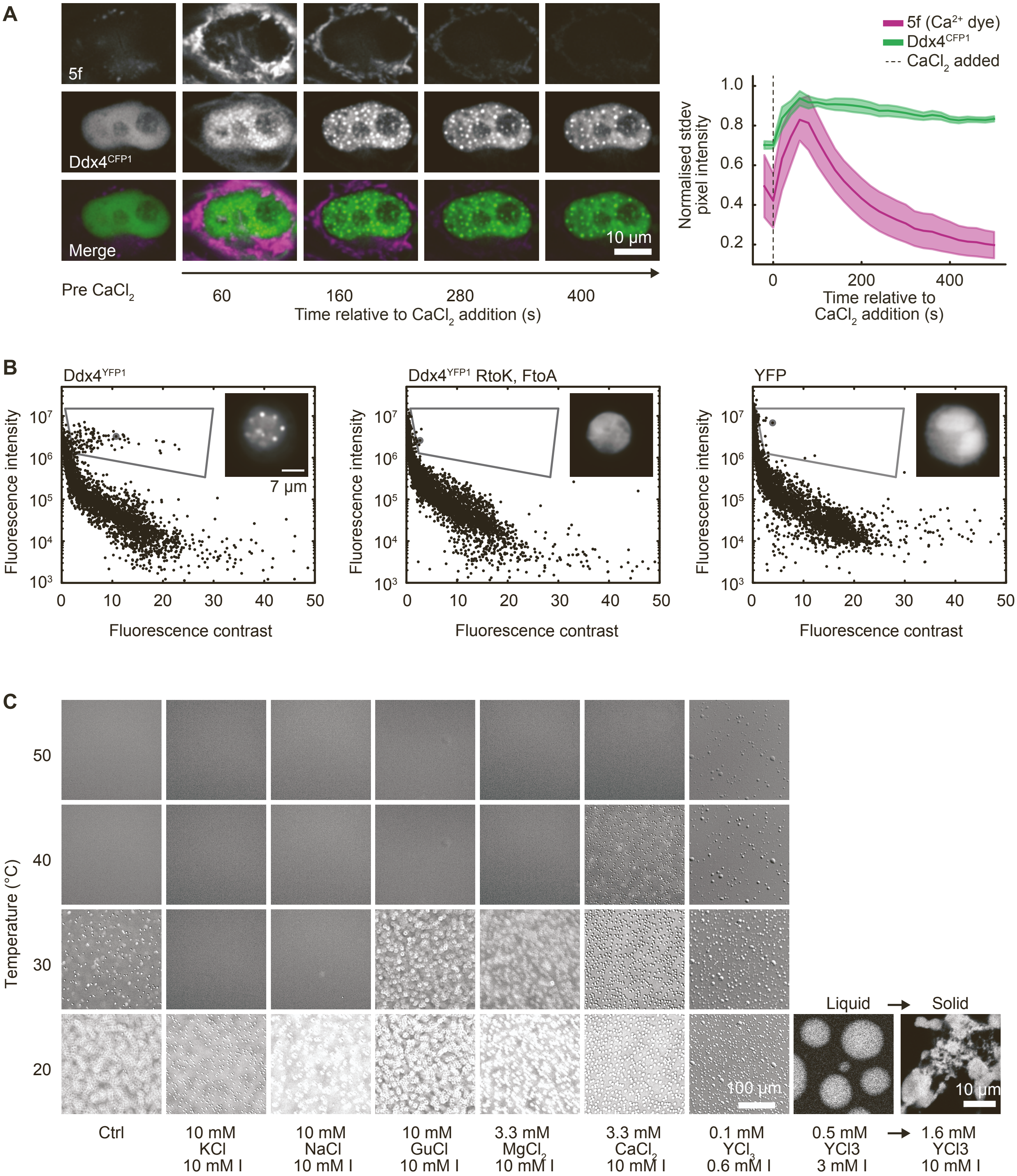
A
5f
Ddx4CFP1
Merge
10 μm
Pre CaCl2
60
160
280
400
Time relative to CaCl2 addition (s)
5f (Ca2+ dye)
Ddx4CFP1
CaCl2 added
Normalised stdev pixel intensity
1.0
0.8
0.6
0.4
0.2
0
200
400
Time relative to CaCl2 addition (s)
B
Ddx4YFP1
Ddx4YFP1 RtoK, FtoA
YFP
Fluorescence intensity
Fluorescence contrast
7 μm
0
10
20
30
40
50
C
Temperature (°C)
50
40
30
20
Liquid
Solid
100 μm
10 μm
Ctrl
10 mM KCl 10 mM I
10 mM NaCl 10 mM I
10 mM GuCl 10 mM I
3.3 mM MgCl2 10 mM I
3.3 mM CaCl2 10 mM I
0.1 mM YCl3 0.6 mM I
0.5 mM YCl3 3 mM I
1.6 mM YCl3 10 mM I

Supp Fig 1. Cation valency modulates Ddx4^{N1} phase separation propensity.

(A) Snapshots of a live cell imaging experiment in which $CaCl_2$ was added to the growth media surrounding live HeLa cells expressing Ddx4^{CFP1} and continuously monitored (left). Quantitation of the fluorescence emission of a Ca^{2+} ion sensitive dye (Fluo 5F) and Ddx4^{CFP1} showing the coincidence of condensate formation and intracellular influx of Ca^{2+} ions. Scale bar 10 μm. Errors indicate SEM.

(B) Flow cytometry data for HeLa cells expressing Ddx4^{YFP1} indicated a subset of cells with high fluorescence intensity and contrast that were not present in cells expressing either an RtoK + FtoA mutant known to reduce the phase separation propensity of Ddx4[1,2], or YFP alone. Images taken during flow cytometry indicated that this subset was reporting on cells with condensate-like features, allowing the number of cells with condensates to be determined in a high throughput manner. Scale bar 7 μm. A grey box indicates the region where cells with condensates were identified and markers with grey outlines indicate the data point that corresponds to each cell image. Data represents a run of 100,000 cells for each construct, with approximately 1% of Ddx4^{YFP1} cells falling within the region outlined with the grey box.

(C) DIC images indicate the effect of ions on the stability of Ddx4^{N1} condensates. Salt concentrations and ionic strengths (I) are indicated. Scale bar 100 μm. Note the transition from liquid-like to solid-like morphology observed upon increasing YCl_3 concentration from 0.5 to 1.6 mM (fluorescence micrographs where the fluorescence signal comes from free Alexa 488 dye, scale bar 10 μm).

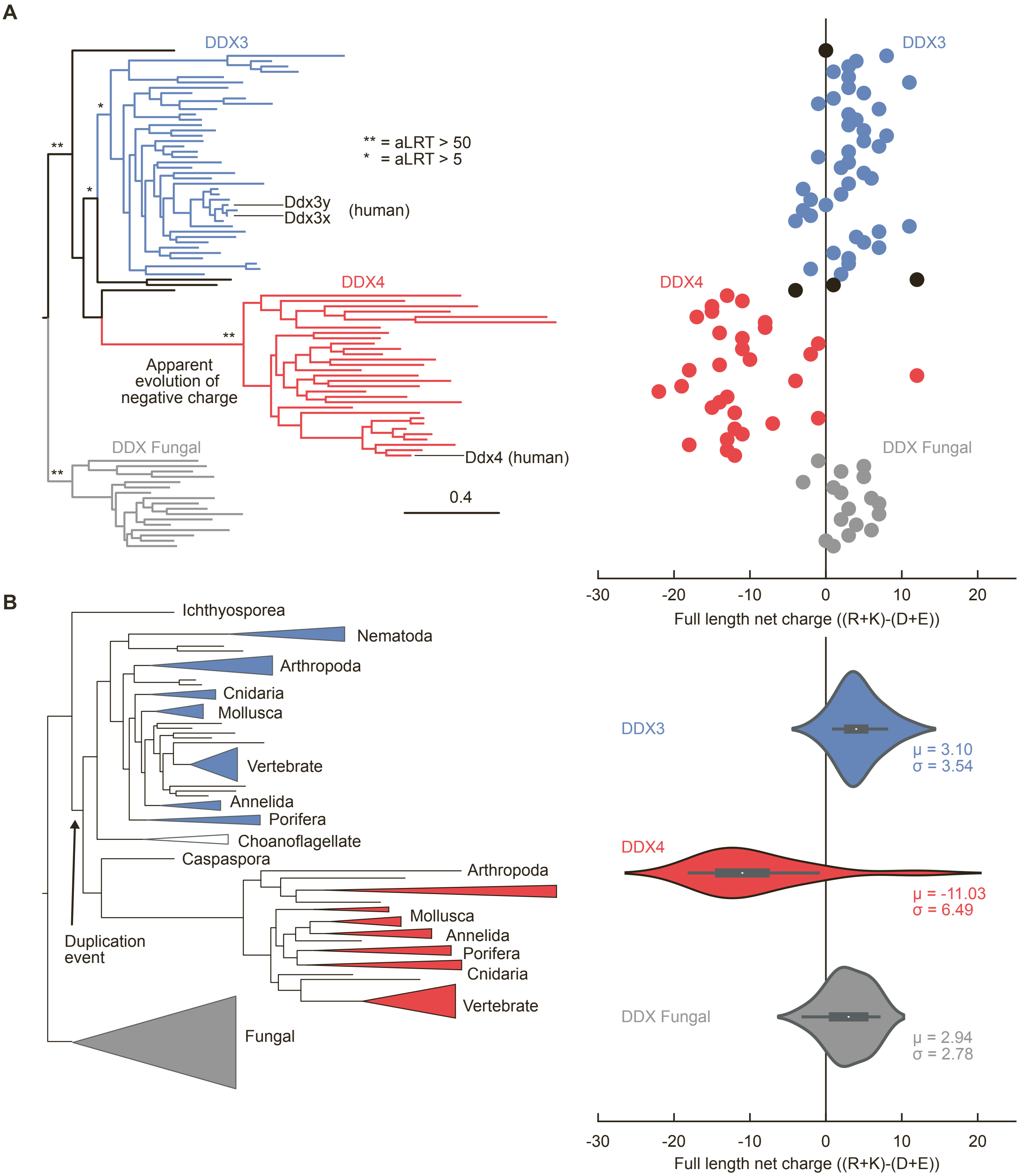
A
DDX3
** = aLRT > 50
* = aLRT > 5
Ddx3y
Ddx3x
(human)
DDX4
Apparent
evolution of
negative charge
DDX Fungal
Ddx4 (human)
0.4
DDX3
DDX4
DDX Fungal
-30
-20
-10
0
10
20
Full length net charge ((R+K)-(D+E))
B
Ichthyosporea
Nematoda
Arthropoda
Cnidaria
Mollusca
Vertebrate
Annelida
Porifera
Choanoflagellate
Caspaspora
Arthropoda
Mollusca
Annelida
Porifera
Cnidaria
Vertebrate
Duplication
event
Fungal
DDX3
μ = 3.10
σ = 3.54
DDX4
μ = -11.03
σ = 6.49
DDX Fungal
μ = 2.94
σ = 2.78
-30
-20
-10
0
10
20
Full length net charge ((R+K)-(D+E))

Supp Fig 2. Evolution of Ddx4 and Ddx3 proteins

(A) Reconstruction of the evolutionary history of DEAD box helicases Ddx4 and Ddx3. Left: Maximum likelihood (ML) tree was constructed based on sequence alignments of the folded domains from 94 animal and fungal species. The animal Ddx4, Ddx3 and fungal clades are shown in red, blue and grey respectively. Eukaryotic clades that are neither animals nor fungi are shown in black. Approximate likelihood-ratio test (aLRT) statistics for selected nodes are indicated. Right: Scatterplot of simple net charge expressed as $(R\ (arg) + K\ (lys)) - (D\ (asp) + E\ (glu))$ for full length Ddx proteins; the values are aligned with branch-tips to which they correspond.

(B) Left: ML phylogeny simplified to show major clades of animals that appear to have one copy of each Ddx paralog. The node that corresponds to the Ddx3/Ddx4 duplication event is indicated. Right: Violin plots of net charge of Ddx4 proteins compared to Ddx3x and fungal Ddx proteins, with mean (μ) and s.d. (σ) indicated.

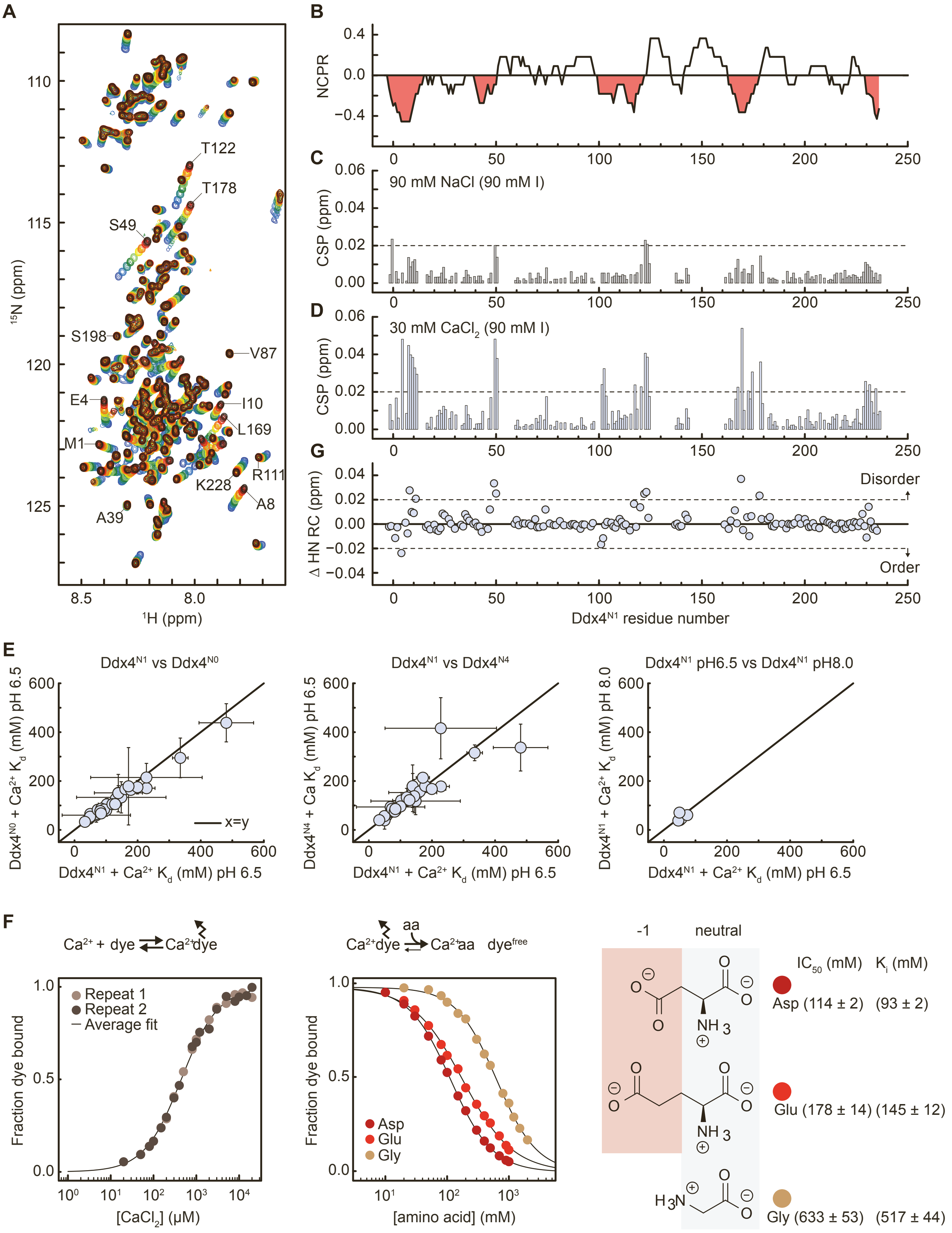
A
B
C
D
G
E
F
T122
T178
S49
S198
V87
E4
I10
L169
M1
R111
K228
A8
A39
^{15}N (ppm)
^{1}H (ppm)
NCPR
90 mM NaCl (90 mM I)
CSP (ppm)
30 mM CaCl$_2$ (90 mM I)
Disorder
Order
Δ HN RC (ppm)
Ddx4^{N1} residue number
Ddx4^{N1} vs Ddx4^{N0}
Ddx4^{N1} vs Ddx4^{N4}
Ddx4^{N1} pH6.5 vs Ddx4^{N1} pH8.0
Ddx4^{N0} + Ca^{2+} K$_d$ (mM) pH 6.5
Ddx4^{N4} + Ca K$_d$ (mM) pH 6.5
Ddx4^{N1} + Ca^{2+} K$_d$ (mM) pH 8.0
Ddx4^{N1} + Ca^{2+} K$_d$ (mM) pH 6.5
x=y
Ca^{2+} + dye ⇄ Ca^{2+}dye
Repeat 1
Repeat 2
Average fit
Fraction dye bound
[CaCl$_2$] (μM)
Ca^{2+}dye → Ca^{2+}aa dye^{free}
aa
Asp
Glu
Gly
[amino acid] (mM)
-1
neutral
IC$_{50}$ (mM) K$_i$ (mM)
Asp (114 ± 2) (93 ± 2)
Glu (178 ± 14) (145 ± 12)
Gly (633 ± 53) (517 ± 44)

Supp Fig 3. The divalent ion Ca^{2+} binds to individual negatively charged sidechains.

(A) ^{1}H-^{15}N BEST-TROSY spectra of $^{15N}Ddx4^{N1}$ at pH 6.5 in the presence of 0 (black), 3.3 (dark red), 10 (red), 30 (orange), 50 (yellow), 100 (light green), 150 mM (green), 250 mM (aqua), 400 mM (light blue), and 600 (dark blue) $CaCl_2$. Residues that show relatively large chemical shift perturbations (CSP) are labelled, as are residues that show minimal CSPs (A39, V87, S198).

(B) Net charge per residue (NCPR, 10 amino acid window, 1 amino acid step) for $Ddx4^{N1}$, calculated using IDDomainSpotter[3].

(C) Per residue CSP for $^{15N}Ddx4^{N1}$ at pH 6.5 at 90 mM additional NaCl compared to 0 mM additional salt. Dashed line indicates a CSP of 0.02 ppm.

(D) Per residue CSP for $^{15N}Ddx4^{N1}$ at pH 6.5 at 30 mM additional $CaCl_2$ compared to 0 mM additional salt. Dashed line indicates a CSP of 0.02 ppm.

(E) Correlations of residue-specific K_d for different samples or conditions compared to the $Ddx4^{N1}$ reference sample. Error bars indicate the estimated standard error for the best-fit value.

(F) Binding isotherm for Ca^{2+} ions and the calcium sensitive dye, Fluo 5N (left). Data from two repeats are shown. Solid line represents the fit values using the mean K_d. A competition-based experiment was used to monitor the binding of amino acids to calcium (right). IC_{50} values were extracted using equation 10 and converted to K_i using equation 11. Carboxylic acid sidechains of D (asp) and E (glu) carry a charge of -1 (pink box). The +1 and -1 charges of the amino and carboxy groups of the amino acid backbone result in a net charge for this region of the molecule of 0 (grey box). Errors represent the error of the fit.

(G) Change in combined ^{1}H and ^{15}N chemical shift relative to the Neighbor Corrected intrinsically disordered protein (IDP) Library (ΔHN RC)[4] at pH 6.5 at 30 mM additional $CaCl_2$ compared to 0 mM additional salt. Dashed line indicates a CSP of 0.02 ppm. Positive and negative values indicate moving towards or away from the reference value for a random coil, respectively.

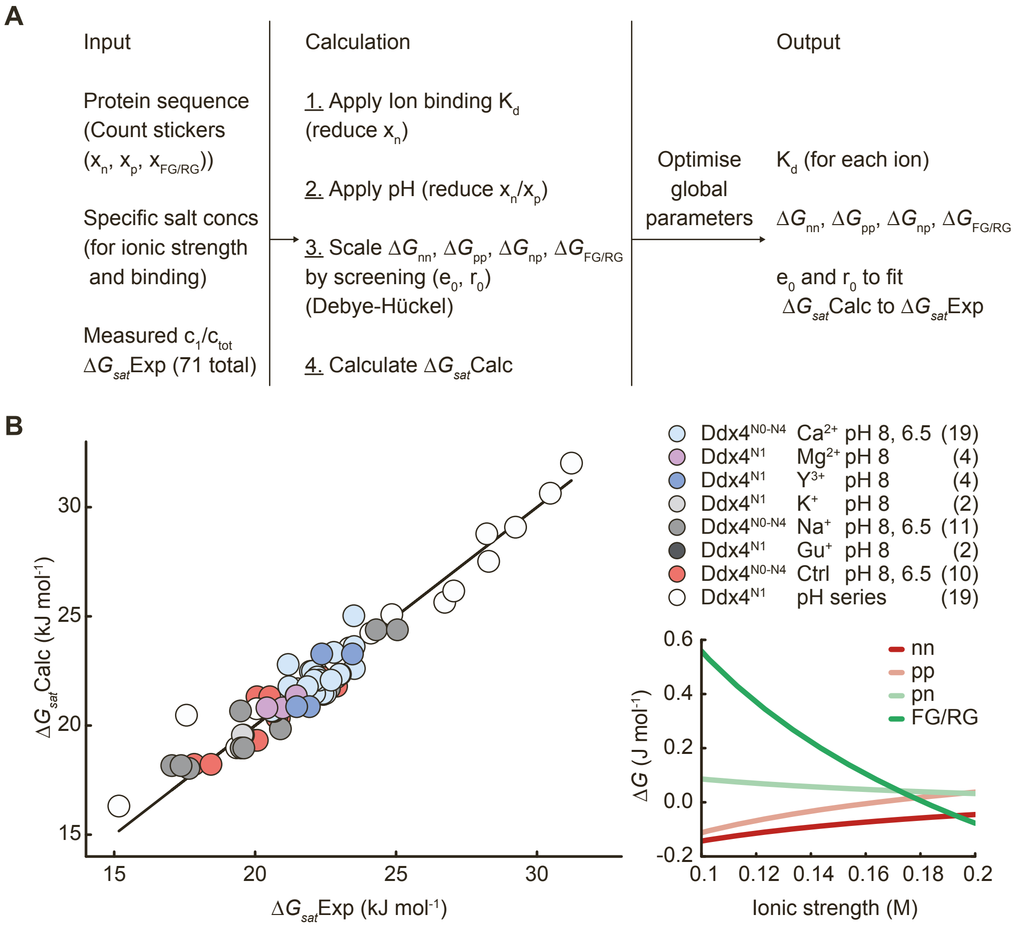
A
Input
Protein sequence (Count stickers (x_n, x_p, $x_{FG/RG}$))
Specific salt concs (for ionic strength and binding)
Measured c_1/c_{tot} ΔG_{sat}Exp (71 total)
Calculation
1. Apply Ion binding K_d (reduce x_n)
2. Apply pH (reduce x_n/x_p)
3. Scale ΔG_{nn}, ΔG_{pp}, ΔG_{np}, $\Delta G_{FG/RG}$ by screening (e_0, r_0) (Debye-Hückel)
4. Calculate ΔG_{sat}Calc
Optimise global parameters
Output
K_d (for each ion)
ΔG_{nn}, ΔG_{pp}, ΔG_{np}, $\Delta G_{FG/RG}$
e_0 and r_0 to fit ΔG_{sat}Calc to ΔG_{sat}Exp
B
$Ddx4^{N0-N4}$ Ca^{2+} pH 8, 6.5 (19)
$Ddx4^{N1}$ Mg^{2+} pH 8 (4)
$Ddx4^{N1}$ Y^{3+} pH 8 (4)
$Ddx4^{N1}$ K^{+} pH 8 (2)
$Ddx4^{N0-N4}$ Na^{+} pH 8, 6.5 (11)
$Ddx4^{N1}$ Gu^{+} pH 8 (2)
$Ddx4^{N0-N4}$ Ctrl pH 8, 6.5 (10)
$Ddx4^{N1}$ pH series (19)
ΔG_{sat}Calc (kJ mol^{-1})
ΔG_{sat}Exp (kJ mol^{-1})
15
20
25
30
ΔG (J mol^{-1})
Ionic strength (M)
0.6
0.4
0.2
0.0
-0.2
0.1
0.12
0.14
0.16
0.18
0.2
nn
pp
pn
FG/RG

Supp Fig 4. Modeling condensate stability.
(A) Schematic representation of the stickers and spacers model used to explain condensate stability.
(B) Correlation of experimental and calculated ΔG_{sat}, fitted values, and the impact of ionic strength on attractive and repulsive interactions in the model.

A

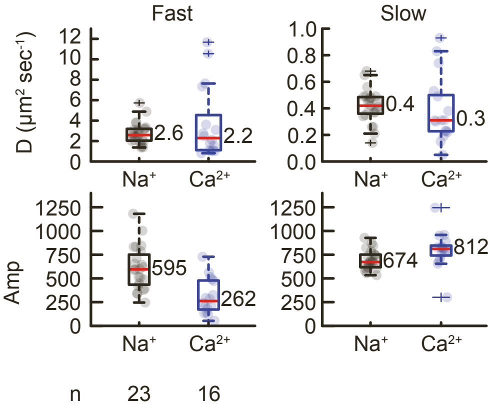

Chi squared values for Na^+ versus Ca^{2+} FRAP datasets:

	Na^+ mono	Na^+ bi	Na^+ ratio: mono/bi	Ca^{2+} mono	Ca^{2+} bi	Ca^{2+} ratio: mono/bi
Mean	124041	14575	8.1	40392	9317	4.4
SD	86039	5262	2.6	27526	4709	1.6

B

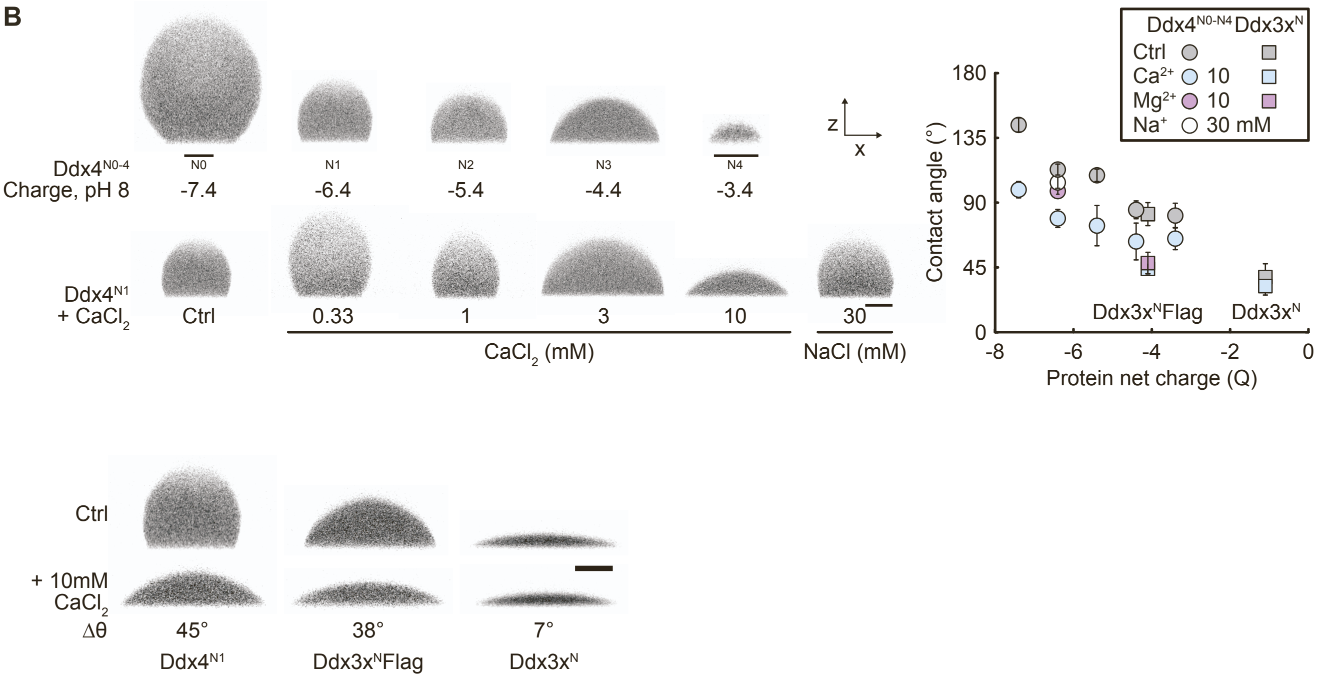

C

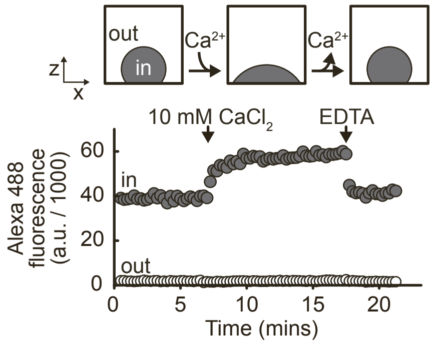

D

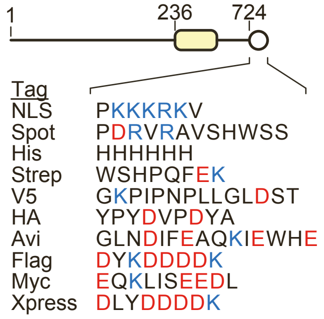

E

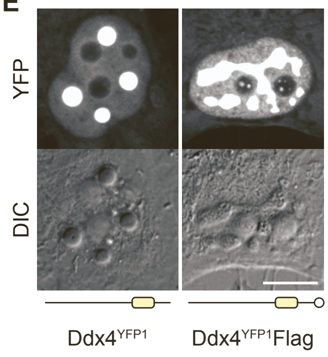

Supp Fig 5. Emergent properties of Ddx4$^{N0\text{-}N4}$ biomolecular condensates.

(A) Box plots showing fast and slow diffusion coefficients ($\mu m^2\ sec^{-1}$) and amplitudes from biexponential fits of individual FRAP curves. Red bar indicates median values (indicated for each dataset), box limits indicate interquartile range, whiskers indicate max/min values, crosses indicate outliers. n indicates the number of condensates measured. Comparison of chi squared values from mono and bi exponential fits are tabulated.

(B) Confocal fluorescence microscopy xz images of Ddx4$^{N0\text{-}N4}$ charge series, Ddx3x^{N} and Ddx3x^{N}Flag condensates sitting on siliconized glass coverslips indicate the correlation between divalent ion addition, net charge and condensate contact angles. Image contrast, generated using Alexa 488, was adjusted to highlight the profile of the condensates. Scale bars apply to the respective rows of images and indicate 5 µm. Ddx4^{N0} has a unique scale bar in the top row. Plot showing droplet contact angles following addition of 30 mM NaCl, 10 mM $MgCl_2$ or 10 mM $CaCl_2$. Error bars indicate standard deviation.

(C) Summary of the data from Movie S1. Addition of 10 mM $CaCl_2$ to a sample containing Ddx4^{N1} condensates increased the maximum Alexa 488 fluorescence intensity originating inside Ddx4^{N1} condensates (grey) whereas no change in Alexa 488 fluorescence intensity was observed in the dilute phase surrounding Ddx4^{N1} condensates (white). Addition of EDTA (20 mM) returned the condensates to their initial appearance and Alexa 488 fluorescence intensity (partitioning).

(D) Ddx4^{YFP1} was tagged at its C-terminus with a variety of common peptide affinity purification tags. Position and sequences of the tags are indicated. Full protein sequences are given in Table S1.

(E). Morphologies of Ddx4^{YFP1} and Ddx4^{YFP1}Flag condensates inside HeLa cells. The spherical appearance of Ddx4^{YFP1} condensates is consistent with a higher surface tension than the less stable Ddx4^{YFP1}Flag condensates.

Protein name	Sequence
Ddx4^{N1}	GAMGSMGDEDWEAEINPHMSSYVPIFEKDRYSGENGDNFNRTPASSSEMDDGPSRRDHFMKSGFASGRNFGNRDAGECNKR DNTSTMGGFGVGKSFGNRGFSNSRFEDGDSSGFWRESSNDCEDNPTRNRGFSKRGGYRDGNNSEASGPYRRGGRGSFRGCRG GFGLGSPNNDLDPDECMQRTGGLFGSRRPVLSGTGNGDTSQSRSGSGSERGGYKGLNEEVITGSGKNSWKSEAEGGES
Ddx3x^{N}	GAMGSMSHVAVENALGLDQQFAGLDLNSSDNQSGGSTASKGRYIPPHLRNREATKGFYDKDSSGWSSSKDKDAYSSFGSRSDS RGKSSFFSDRGSGSRGRFDDRGRSDYDGIGSRGDRSGFGKFERGGNSRWCDKSDEDDWS
Ddx3x^{N_FLAG}	GAMGSMSHVAVENALGLDQQFAGLDLNSSDNQSGGSTASKGRYIPPHLRNREATKGFYDKDSSGWSSSKDKDAYSSFGSRSDS RGKSSFFSDRGSGSRGRFDDRGRSDYDGIGSRGDRSGFGKFERGGNSRWCDKSDEDDWSDYKDDDDK
Ddx3y^{N}	GAMGSMSHVVVKNDPELDQQLANLDLNSEKQSGGASTASKGRYIPPHLRNREASKGFHDKDSSGWSCSKDKDAYSSFGSRDSR GKPGYFSERGSGSRGRFDDRGRSDYDGIGNRERPGFGRFERSGHSRWCDKSVEDDWS
Vasa^{N}	GAMGSMSDDWDDEPIVDTRGARGGDWSDDEDTAKSFSGEAEGDGVGGSGGEGGGYQGGNRDVFGRIGGGRGGGAGGYRG GNRDGGGFHGGRREGERDFRGGEGGFRGGQGGSRGGQGGSRGGQGGFRGGEGGFRGRLYENEDGDERRGRLDREERGGER RGRLDREERGGERGERGDGGFARRRRNEDDINNNNNIV
Bel^{N}	GAMGSMSNAINQNGTGLEQQVAGLDLNGGSADYSGPITSKTSTNSVTGGVYVPPHLRGGGGNNNAADAESQGQGQGQGQG FDSRSGNPRQETRDPQQSRGGGGEYRRGGGGGGRGFNRQSGDYGYGSGGGGRRGGGGRFEDNYNGGEFDSRRGGDWNRS GGGGGGGRGFGRGPSYRGGGGGSGSNLNEQTAEDGQAQQQQQPRNDRWQEPERPAGFDGSEGGQSAGGNRSYNNRGER GGGGYNSRWKEGGGSNVDYT
drDdx4^{N}	GAMGSMDDWEEDQSPVVSCSSGFGLGSNGSDGGFKSFYTGGAGNDKSNSEGTEGSSWKMTGDSFRGRGGRGGSRGGRGGF SGFKSEIDENGSDGGWNGGESRGRGRGGFRGGFRSGSRDENDENRNDDGWKGGESRGRGRGGFGGSFRGGFRDGGNEDTG RRGFGRENNENGNDEGGEGRGRGRGGFRGGFRDGGGDESGKRGFGRGGFRGRNEEVFSKVTTADKLDQEG
Ddx4^{N0}	GAMGSMGDEDWEAEINPHMSSYVPIFEKDRYSGENGDNFNRTPASSSEMDDGPSRRDHFMKSGFASGRNFGNRDAGECNKR DNTSTMGGFGVGKSFGNRGFSNSRFEDGDSSGFWRESSNDCEDNPTRNRGFSKRGGYRDGNNSEASGPYRRGGRGSFRGCRG GFGLGSPNNDLDPDECMQRTGGLFGSRRPVLSGTGNGDTSQSRSGSGSERGGYKGLNEEVITGSGKNSWKSEAEGGESSD
Ddx4^{N1}	GAMGSMGDEDWEAEINPHMSSYVPIFEKDRYSGENGDNFNRTPASSSEMDDGPSRRDHFMKSGFASGRNFGNRDAGECNKR DNTSTMGGFGVGKSFGNRGFSNSRFEDGDSSGFWRESSNDCEDNPTRNRGFSKRGGYRDGNNSEASGPYRRGGRGSFRGCRG GFGLGSPNNDLDPDECMQRTGGLFGSRRPVLSGTGNGDTSQSRSGSGSERGGYKGLNEEVITGSGKNSWKSEAEGGES
Ddx4^{N2}	GAMGSMGDEDWEAEINPHMSSYVPIFEKDRYSGENGDNFNRTPASSSEMDDGPSRRDHFMKSGFASGRNFGNRDAGECNKR DNTSTMGGFGVGKSFGNRGFSNSRFEDGDSSGFWRESSNDCEDNPTRNRGFSKRGGYRDGNNSEASGPYRRGGRGSFRGCRG GFGLGSPNNDLDPDECMQRTGGLFGSRRPVLSGTGNGDTSQSRSGSGSERGGYKGLNEEVITGSGKNSWKSEAEGG
Ddx4^{N3}	GAMGSMGDEDWEAEINPHMSSYVPIFEKDRYSGENGDNFNRTPASSSEMDDGPSRRDHFMKSGFASGRNFGNRDAGECNKR DNTSTMGGFGVGKSFGNRGFSNSRFEDGDSSGFWRESSNDCEDNPTRNRGFSKRGGYRDGNNSEASGPYRRGGRGSFRGCRG GFGLGSPNNDLDPDECMQRTGGLFGSRRPVLSGTGNGDTSQSRSGSGSERGGYKGLNEEVITGSGKNSWKSEA
Ddx4^{N4}	GAMGSMGDEDWEAEINPHMSSYVPIFEKDRYSGENGDNFNRTPASSSEMDDGPSRRDHFMKSGFASGRNFGNRDAGECNKR DNTSTMGGFGVGKSFGNRGFSNSRFEDGDSSGFWRESSNDCEDNPTRNRGFSKRGGYRDGNNSEASGPYRRGGRGSFRGCRG GFGLGSPNNDLDPDECMQRTGGLFGSRRPVLSGTGNGDTSQSRSGSGSERGGYKGLNEEVITGSGKNSWKS
YFP (mCitrine; recombinantly expressed and purified)	GAMGSMVSKGEELFTGVVPILVELDGDVNGHKFSVSGEGEGDATYGKLTLKFICTTGKLPVPWPTLVTTFGYGLMCFARYPDHM KQHDFFKSAMPEGYVQERTIFFKDDGNYKTRAEVKFEGDTLVNRIELKGIDFKEDGNILGHKLEYNYNSHNVYIMADKQKNGIKV NFKIRHNIEDGSVQLADHYQQNTPIGDGPVLLPDNHYLSYQSKLSKDPNEKRDHMVLLEFVTAAGIT
Ddx4^{YFP0}	MGDEDWEAEINPHMSSYVPIFEKDRYSGENGDNFNRTPASSSEMDDGPSRRDHFMKSGFASGRNFGNRDAGECNKRDNTST MGGFGVGKSFGNRGFSNSRFEDGDSSGFWRESSNDCEDNPTRNRGFSKRGGYRDGNNSEASGPYRRGGRGSFRGCRGGFGLG SPNNDLDPDECMQRTGGLFGSRRPVLSGTGNGDTSQSRSGSGSERGGYKGLNEEVITGSGKNSWKSEAEGGESSDMVSKGEELF TGVVPILVELDGDVNGHKFSVSGEGEGDATYGKLTLKFICTTGKLPVPWPTLVTTFGYGLMCFARYPDHMKQHDFFKSAMPEGY VQERTIFFKDDGNYKTRAEVKFEGDTLVNRIELKGIDFKEDGNILGHKLEYNYNSHNVYIMADKQKNGIKVNFKIRHNIEDGSVQLA DHYQQNTPIGDGPVLLPDNHYLSYQSKLSKDPNEKRDHMVLLEFVTAAGITFSTYIPGFSGSTRGNVFASVDTRKGKSTLNTAGFSS SQAPNPVDDESWD
Ddx4^{YFP1}	MGDEDWEAEINPHMSSYVPIFEKDRYSGENGDNFNRTPASSSEMDDGPSRRDHFMKSGFASGRNFGNRDAGECNKRDNTST MGGFGVGKSFGNRGFSNSRFEDGDSSGFWRESSNDCEDNPTRNRGFSKRGGYRDGNNSEASGPYRRGGRGSFRGCRGGFGLG SPNNDLDPDECMQRTGGLFGSRRPVLSGTGNGDTSQSRSGSGSERGGYKGLNEEVITGSGKNSWKSEAEGGESMVSKGEELFTG VVPILVELDGDVNGHKFSVSGEGEGDATYGKLTLKFICTTGKLPVPWPTLVTTFGYGLMCFARYPDHMKQHDFFKSAMPEGYVQ ERTIFFKDDGNYKTRAEVKFEGDTLVNRIELKGIDFKEDGNILGHKLEYNYNSHNVYIMADKQKNGIKVNFKIRHNIEDGSVQLADH YQQNTPIGDGPVLLPDNHYLSYQSKLSKDPNEKRDHMVLLEFVTAAGITFSTYIPGFSGSTRGNVFASVDTRKGKSTLNTAGFSSSQ APNPVDDESWD
Ddx4^{YFP2}	MGDEDWEAEINPHMSSYVPIFEKDRYSGENGDNFNRTPASSSEMDDGPSRRDHFMKSGFASGRNFGNRDAGECNKRDNTST MGGFGVGKSFGNRGFSNSRFEDGDSSGFWRESSNDCEDNPTRNRGFSKRGGYRDGNNSEASGPYRRGGRGSFRGCRGGFGLG SPNNDLDPDECMQRTGGLFGSRRPVLSGTGNGDTSQSRSGSGSERGGYKGLNEEVITGSGKNSWKSEAEGGMVSKGEELFTGV VPILVELDGDVNGHKFSVSGEGEGDATYGKLTLKFICTTGKLPVPWPTLVTTFGYGLMCFARYPDHMKQHDFFKSAMPEGYVQE RTIFFKDDGNYKTRAEVKFEGDTLVNRIELKGIDFKEDGNILGHKLEYNYNSHNVYIMADKQKNGIKVNFKIRHNIEDGSVQLADHY

	QQNTPIGDGPVLLPDNHYLSYQSKLSKDPNEKRDHMVLLEFVTAAGITFSTYIPGFSGSTRGNVFASVDTRKGKSTLNTAGFSSSQA<br>PNPVDDESWD
Ddx4^{YFP3}	MGDEDWEAEINPHMSSYVPIFEKDRYSGENGDNFNRTPASSSEMDDGPSRRDHFMKSGFASGRNFGNRDAGECNKRDNTST<br>MGGFGVGKSFGNRGFSNSRFEDGDSSGFWRESSNDCEDNPTRNRGFSKRGGYRDGNNSEASGPYRRGGRGSFRGCRGGFGLG<br>SPNNDLDPDECMQRTGGLFGSRRPVLSGTGNGDTSQSRSGSGSERGGYKGLNEEVITGSGKNSWKSEAMVSKGEELFTGVVPIL<br>VELDGDVNGHKFSVSGEGEGDATYGKLTLKFICTTGKLPVPWPTLVTTFGYGLMCFARYPDHMKQHDFFKSAMPEGYVQERTIFF<br>KDDGNYKTRAEVKFEGDTLVNRIELKGIDFKEDGNILGHKLEYNYNSHNVYIMADKQKNGIKVNFKIRHNIEDGSVQLADHYQQN<br>TPIGDGPVLLPDNHYLSYQSKLSKDPNEKRDHMVLLEFVTAAGITFSTYIPGFSGSTRGNVFASVDTRKGKSTLNTAGFSSSQAPNP<br>VDDESWD
Ddx4^{YFP4}	MGDEDWEAEINPHMSSYVPIFEKDRYSGENGDNFNRTPASSSEMDDGPSRRDHFMKSGFASGRNFGNRDAGECNKRDNTST<br>MGGFGVGKSFGNRGFSNSRFEDGDSSGFWRESSNDCEDNPTRNRGFSKRGGYRDGNNSEASGPYRRGGRGSFRGCRGGFGLG<br>SPNNDLDPDECMQRTGGLFGSRRPVLSGTGNGDTSQSRSGSGSERGGYKGLNEEVITGSGKNSWKSMVSKGEELFTGVVPILVE<br>LDGDVNGHKFSVSGEGEGDATYGKLTLKFICTTGKLPVPWPTLVTTFGYGLMCFARYPDHMKQHDFFKSAMPEGYVQERTIFFK<br>DDGNYKTRAEVKFEGDTLVNRIELKGIDFKEDGNILGHKLEYNYNSHNVYIMADKQKNGIKVNFKIRHNIEDGSVQLADHYQQNT<br>PIGDGPVLLPDNHYLSYQSKLSKDPNEKRDHMVLLEFVTAAGITFSTYIPGFSGSTRGNVFASVDTRKGKSTLNTAGFSSSQAPNPV<br>DDESWD
YFP (mCitrine; expressed in HeLa cells)	MVSKGEELFTGVVPILVELDGDVNGHKFSVSGEGEGDATYGKLTLKFICTTGKLPVPWPTLVTTFGYGLMCFARYPDHMKQHDFF<br>KSAMPEGYVQERTIFFKDDGNYKTRAEVKFEGDTLVNRIELKGIDFKEDGNILGHKLEYNYNSHNVYIMADKQKNGIKVNFKIRHNI<br>EDGSVQLADHYQQNTPIGDGPVLLPDNHYLSYQSKLSKDPNEKRDHMVLLEFVTAAGITLERPLESRGPV
Ddx4^{YFP1}RtoK, FtoA	MGDEDWEAEINPHMSSYVPIFEKDRYSGENGDNFNRTPASSSEMDDGPSKRDHFMKSGAASGKNAGNRDAGECNKRDNTST<br>MGGAGVGKSAGNKGASNSKFEDGDSSGFWRESSNDCEDNPTRNKGASKKGGYRDGNNSEASGPYRKGGKGSAKGCKGGAGL<br>GSPNNDLDPDECMQRTGGLAGSKRPVLSGTGNGDTSQSKSGSGSEKGGYKGLNEEVITGSGKNSWKSEAEGGESSDTQGPKVT<br>LQMVSKGEELFTGVVPILVELDGDVNGHKFSVSGEGEGDATYGKLTLKFICTTGKLPVPWPTLVTTFGYGLMCFARYPDHMKQH<br>DFFKSAMPEGYVQERTIFFKDDGNYKTRAEVKFEGDTLVNRIELKGIDFKEDGNILGHKLEYNYNSHNVYIMADKQKNGIKVNFKIR<br>HNIEDGSVQLADHYQQNTPIGDGPVLLPDNHYLSYQSKLSKDPNEKRDHMVLLEFVTAAGITLEASTYIPGASGSTKGNVFASVDT<br>RKGKSTLNTAGFSSSQAPNPVDDESWD
Ddx4^{CFP1}	MGDEDWEAEINPHMSSYVPIFEKDRYSGENGDNFNRTPASSSEMDDGPSRRDHFMKSGFASGRNFGNRDAGECNKRDNTST<br>MGGFGVGKSFGNRGFSNSRFEDGDSSGFWRESSNDCEDNPTRNRGFSKRGGYRDGNNSEASGPYRRGGRGSFRGCRGGFGLG<br>SPNNDLDPDECMQRTGGLFGSRRPVLSGTGNGDTSQSRSGSGSERGGYKGLNEEVITGSGKNSWKSEAEGGESMVSKGEELFTG<br>VVPILVELDGDVNGHKFSVSGEGEGDATYGKLTLKFICTTGKLPVPWPTLVTTLTWGVQCFARYPDHMKQHDFFKSAMPEGYVQ<br>ERTIFFKDDGNYKTRAEVKFEGDTLVNRIELKGIDFKEDGNILGHKLEYNAISDNVYITADKQKNGIKANFKIRHNIEDGSVQLADHY<br>QQNTPIGDGPVLLPDNHYLSTQSKLSKDPNEKRDHMVLLEFVTAAGITFSTYIPGFSGSTRGNVFASVDTRKGKSTLNTAGFSSSQA<br>PNPVDDESWD
Ddx4^{YFP1}His	MGDEDWEAEINPHMSSYVPIFEKDRYSGENGDNFNRTPASSSEMDDGPSRRDHFMKSGFASGRNFGNRDAGECNKRDNTST<br>MGGFGVGKSFGNRGFSNSRFEDGDSSGFWRESSNDCEDNPTRNRGFSKRGGYRDGNNSEASGPYRRGGRGSFRGCRGGFGLG<br>SPNNDLDPDECMQRTGGLFGSRRPVLSGTGNGDTSQSRSGSGSERGGYKGLNEEVITGSGKNSWKSEAEGGESMVSKGEELFTG<br>VVPILVELDGDVNGHKFSVSGEGEGDATYGKLTLKFICTTGKLPVPWPTLVTTFGYGLMCFARYPDHMKQHDFFKSAMPEGYVQ<br>ERTIFFKDDGNYKTRAEVKFEGDTLVNRIELKGIDFKEDGNILGHKLEYNYNSHNVYIMADKQKNGIKVNFKIRHNIEDGSVQLADH<br>YQQNTPIGDGPVLLPDNHYLSYQSKLSKDPNEKRDHMVLLEFVTAAGITFSTYIPGFSGSTRGNVFASVDTRKGKSTLNTAGFSSSQ<br>APNPVDDESWDGSGSGHHHHHH
Ddx4^{YFP1}Myc	MGDEDWEAEINPHMSSYVPIFEKDRYSGENGDNFNRTPASSSEMDDGPSRRDHFMKSGFASGRNFGNRDAGECNKRDNTST<br>MGGFGVGKSFGNRGFSNSRFEDGDSSGFWRESSNDCEDNPTRNRGFSKRGGYRDGNNSEASGPYRRGGRGSFRGCRGGFGLG<br>SPNNDLDPDECMQRTGGLFGSRRPVLSGTGNGDTSQSRSGSGSERGGYKGLNEEVITGSGKNSWKSEAEGGESMVSKGEELFTG<br>VVPILVELDGDVNGHKFSVSGEGEGDATYGKLTLKFICTTGKLPVPWPTLVTTFGYGLMCFARYPDHMKQHDFFKSAMPEGYVQ<br>ERTIFFKDDGNYKTRAEVKFEGDTLVNRIELKGIDFKEDGNILGHKLEYNYNSHNVYIMADKQKNGIKVNFKIRHNIEDGSVQLADH<br>YQQNTPIGDGPVLLPDNHYLSYQSKLSKDPNEKRDHMVLLEFVTAAGITFSTYIPGFSGSTRGNVFASVDTRKGKSTLNTAGFSSSQ<br>APNPVDDESWDGSGSGEQKLISEEDL
Ddx4^{YFP1}Flag	MGDEDWEAEINPHMSSYVPIFEKDRYSGENGDNFNRTPASSSEMDDGPSRRDHFMKSGFASGRNFGNRDAGECNKRDNTST<br>MGGFGVGKSFGNRGFSNSRFEDGDSSGFWRESSNDCEDNPTRNRGFSKRGGYRDGNNSEASGPYRRGGRGSFRGCRGGFGLG<br>SPNNDLDPDECMQRTGGLFGSRRPVLSGTGNGDTSQSRSGSGSERGGYKGLNEEVITGSGKNSWKSEAEGGESMVSKGEELFTG<br>VVPILVELDGDVNGHKFSVSGEGEGDATYGKLTLKFICTTGKLPVPWPTLVTTFGYGLMCFARYPDHMKQHDFFKSAMPEGYVQ<br>ERTIFFKDDGNYKTRAEVKFEGDTLVNRIELKGIDFKEDGNILGHKLEYNYNSHNVYIMADKQKNGIKVNFKIRHNIEDGSVQLADH<br>YQQNTPIGDGPVLLPDNHYLSYQSKLSKDPNEKRDHMVLLEFVTAAGITFSTYIPGFSGSTRGNVFASVDTRKGKSTLNTAGFSSSQ<br>APNPVDDESWDGSGSGDYKDDDDK
Ddx4^{YFP1}HA	MGDEDWEAEINPHMSSYVPIFEKDRYSGENGDNFNRTPASSSEMDDGPSRRDHFMKSGFASGRNFGNRDAGECNKRDNTST<br>MGGFGVGKSFGNRGFSNSRFEDGDSSGFWRESSNDCEDNPTRNRGFSKRGGYRDGNNSEASGPYRRGGRGSFRGCRGGFGLG<br>SPNNDLDPDECMQRTGGLFGSRRPVLSGTGNGDTSQSRSGSGSERGGYKGLNEEVITGSGKNSWKSEAEGGESMVSKGEELFTG<br>VVPILVELDGDVNGHKFSVSGEGEGDATYGKLTLKFICTTGKLPVPWPTLVTTFGYGLMCFARYPDHMKQHDFFKSAMPEGYVQ<br>ERTIFFKDDGNYKTRAEVKFEGDTLVNRIELKGIDFKEDGNILGHKLEYNYNSHNVYIMADKQKNGIKVNFKIRHNIEDGSVQLADH<br>YQQNTPIGDGPVLLPDNHYLSYQSKLSKDPNEKRDHMVLLEFVTAAGITFSTYIPGFSGSTRGNVFASVDTRKGKSTLNTAGFSSSQ<br>APNPVDDESWDGSGSGYPYDVPDYA

Ddx4YFP1Strep	MGDEDWEAEINPHMSSYVPIFEKDRYSGENGDNFNRTPASSSEMDDGPSRRDHFMKSGFASGRNFGNRDAGECNKRDNTST<br>MGGFGVGKSFGNRGFSNSRFEDGDSSGFWRESSNDCEDNPTRNRGFSKRGGYRDGNNSEASGPYRRGGRGSFRGCRGGFGLG<br>SPNNDLDPDECMQRTGGLFGSRRPVLSGTGNGDTSQSRSGSGSERGGYKGLNEEVITGSGKNSWKSEAEGGESMVSKGEELFTG<br>VVPILVELDGDVNGHKFSVSGEGEGDATYGKLTLKFICTTGKLPVPWPTLVTTFGYGLMCFARYPDHMKQHDFFKSAMPEGYVQ<br>ERTIFFKDDGNYKTRAEVKFEGDTLVNRIELKGIDFKEDGNILGHKLEYNYNSHNVYIMADKQKNGIKVNFKIRHNIEDGSVQLADH<br>YQQNTPIGDGPVLLPDNHYLSYQSKLSKDPNEKRDHMVLLEFVTAAGITFSTYIPGFSGSTRGNVFASVDTRKGKSTLNTAGFSSSQ<br>APNPVDDESWDGSGSGWSHPQFEK
Ddx4YFP1Xpress	MGDEDWEAEINPHMSSYVPIFEKDRYSGENGDNFNRTPASSSEMDDGPSRRDHFMKSGFASGRNFGNRDAGECNKRDNTST<br>MGGFGVGKSFGNRGFSNSRFEDGDSSGFWRESSNDCEDNPTRNRGFSKRGGYRDGNNSEASGPYRRGGRGSFRGCRGGFGLG<br>SPNNDLDPDECMQRTGGLFGSRRPVLSGTGNGDTSQSRSGSGSERGGYKGLNEEVITGSGKNSWKSEAEGGESMVSKGEELFTG<br>VVPILVELDGDVNGHKFSVSGEGEGDATYGKLTLKFICTTGKLPVPWPTLVTTFGYGLMCFARYPDHMKQHDFFKSAMPEGYVQ<br>ERTIFFKDDGNYKTRAEVKFEGDTLVNRIELKGIDFKEDGNILGHKLEYNYNSHNVYIMADKQKNGIKVNFKIRHNIEDGSVQLADH<br>YQQNTPIGDGPVLLPDNHYLSYQSKLSKDPNEKRDHMVLLEFVTAAGITFSTYIPGFSGSTRGNVFASVDTRKGKSTLNTAGFSSSQ<br>APNPVDDESWDGSGSGDLYDDDDK
Ddx4YFP1Avi	MGDEDWEAEINPHMSSYVPIFEKDRYSGENGDNFNRTPASSSEMDDGPSRRDHFMKSGFASGRNFGNRDAGECNKRDNTST<br>MGGFGVGKSFGNRGFSNSRFEDGDSSGFWRESSNDCEDNPTRNRGFSKRGGYRDGNNSEASGPYRRGGRGSFRGCRGGFGLG<br>SPNNDLDPDECMQRTGGLFGSRRPVLSGTGNGDTSQSRSGSGSERGGYKGLNEEVITGSGKNSWKSEAEGGESMVSKGEELFTG<br>VVPILVELDGDVNGHKFSVSGEGEGDATYGKLTLKFICTTGKLPVPWPTLVTTFGYGLMCFARYPDHMKQHDFFKSAMPEGYVQ<br>ERTIFFKDDGNYKTRAEVKFEGDTLVNRIELKGIDFKEDGNILGHKLEYNYNSHNVYIMADKQKNGIKVNFKIRHNIEDGSVQLADH<br>YQQNTPIGDGPVLLPDNHYLSYQSKLSKDPNEKRDHMVLLEFVTAAGITFSTYIPGFSGSTRGNVFASVDTRKGKSTLNTAGFSSSQ<br>APNPVDDESWDGSGSGGLNDIFEAQKIEWHE
Ddx4YFP1V5	MGDEDWEAEINPHMSSYVPIFEKDRYSGENGDNFNRTPASSSEMDDGPSRRDHFMKSGFASGRNFGNRDAGECNKRDNTST<br>MGGFGVGKSFGNRGFSNSRFEDGDSSGFWRESSNDCEDNPTRNRGFSKRGGYRDGNNSEASGPYRRGGRGSFRGCRGGFGLG<br>SPNNDLDPDECMQRTGGLFGSRRPVLSGTGNGDTSQSRSGSGSERGGYKGLNEEVITGSGKNSWKSEAEGGESMVSKGEELFTG<br>VVPILVELDGDVNGHKFSVSGEGEGDATYGKLTLKFICTTGKLPVPWPTLVTTFGYGLMCFARYPDHMKQHDFFKSAMPEGYVQ<br>ERTIFFKDDGNYKTRAEVKFEGDTLVNRIELKGIDFKEDGNILGHKLEYNYNSHNVYIMADKQKNGIKVNFKIRHNIEDGSVQLADH<br>YQQNTPIGDGPVLLPDNHYLSYQSKLSKDPNEKRDHMVLLEFVTAAGITFSTYIPGFSGSTRGNVFASVDTRKGKSTLNTAGFSSSQ<br>APNPVDDESWDGSGSGGKPIPNPLLGLDST
Ddx4YFP1NLS	MGDEDWEAEINPHMSSYVPIFEKDRYSGENGDNFNRTPASSSEMDDGPSRRDHFMKSGFASGRNFGNRDAGECNKRDNTST<br>MGGFGVGKSFGNRGFSNSRFEDGDSSGFWRESSNDCEDNPTRNRGFSKRGGYRDGNNSEASGPYRRGGRGSFRGCRGGFGLG<br>SPNNDLDPDECMQRTGGLFGSRRPVLSGTGNGDTSQSRSGSGSERGGYKGLNEEVITGSGKNSWKSEAEGGESMVSKGEELFTG<br>VVPILVELDGDVNGHKFSVSGEGEGDATYGKLTLKFICTTGKLPVPWPTLVTTFGYGLMCFARYPDHMKQHDFFKSAMPEGYVQ<br>ERTIFFKDDGNYKTRAEVKFEGDTLVNRIELKGIDFKEDGNILGHKLEYNYNSHNVYIMADKQKNGIKVNFKIRHNIEDGSVQLADH<br>YQQNTPIGDGPVLLPDNHYLSYQSKLSKDPNEKRDHMVLLEFVTAAGITFSTYIPGFSGSTRGNVFASVDTRKGKSTLNTAGFSSSQ<br>APNPVDDESWDGSGSGPKKKRKV
Ddx4YFP1Spot	MGDEDWEAEINPHMSSYVPIFEKDRYSGENGDNFNRTPASSSEMDDGPSRRDHFMKSGFASGRNFGNRDAGECNKRDNTST<br>MGGFGVGKSFGNRGFSNSRFEDGDSSGFWRESSNDCEDNPTRNRGFSKRGGYRDGNNSEASGPYRRGGRGSFRGCRGGFGLG<br>SPNNDLDPDECMQRTGGLFGSRRPVLSGTGNGDTSQSRSGSGSERGGYKGLNEEVITGSGKNSWKSEAEGGESMVSKGEELFTG<br>VVPILVELDGDVNGHKFSVSGEGEGDATYGKLTLKFICTTGKLPVPWPTLVTTFGYGLMCFARYPDHMKQHDFFKSAMPEGYVQ<br>ERTIFFKDDGNYKTRAEVKFEGDTLVNRIELKGIDFKEDGNILGHKLEYNYNSHNVYIMADKQKNGIKVNFKIRHNIEDGSVQLADH<br>YQQNTPIGDGPVLLPDNHYLSYQSKLSKDPNEKRDHMVLLEFVTAAGITFSTYIPGFSGSTRGNVFASVDTRKGKSTLNTAGFSSSQ<br>APNPVDDESWDGSGSGPDRVRAVSHWSS

Table S1. Amino acid sequences of proteins used in the paper. Residual cloning tags at the N-termini of recombinantly expressed and purified protein sequences (GAMGS) are underlined. Residue numbering indicated in the main text starts from the first non-underlined amino acid.

Protein name / amino acid numbers in construct	Ddx4^{N1}/6-241 vs other sequences used in the study	Alignment length	% identity	% similarity
>Ddx4^{N1}/1-241	Ddx4^{N1}/6-241	236	100.0	100.0
>Ddx3x^{N}/1-142	Ddx3x^{N}/6-142	237	12.2	23.6
>Ddx3x^{N}_FLAG/1-150	Ddx3x^{N}_FLAG/6-150	237	12.2	24.5
>Ddx3y^{N}/1-140	Ddx3y^{N}/6-140	237	10.6	22.8
>Vasa^{N}/1-197	Vasa^{N}/6-197	247	17.0	30.8
>Bel^{N}/1-257	Bel^{N}/6-257	288	15.6	32.3
>drDdx4^{N}/1-230	drDdx4^{N}/6-230	255	20.8	38.4
>Ddx4^{N0}_238/1-243	Ddx4^{N0}_238/6-243	238	99.2	99.2
>Ddx4^{N1}_236/1-241	Ddx4^{N1}_236/6-241	236	100.0	100.0
>Ddx4^{N2}_234/1-239	Ddx4^{N2}_234/6-239	236	99.2	99.2
>Ddx4^{N3}_231/1-236	Ddx4^{N3}_231/6-236	236	97.9	97.9
>Ddx4^{N4}_229/1-234	Ddx4^{N4}_229/6-234	236	97.0	97.0

Table S2. Amino acid sequence similarity and identity scores for Ddx4 and Ddx3 homologues, related to Figure 2. Amino acid groupings were: AVLIM, FYW, GSTCHNQ, P, KR, DE. https://www.bioinformatics.org/sms2/ident_sim.html, ref [5].

Parameter	Fitted value	±	$x0$	Mean	Stdev
ΔG_{nn}	-21.91	1.12	-22.45	-22.43	1.07
ΔG_{pp}	-27.02	3.64	-26.13	-25.79	3.23
ΔG_{pn}	16.22	0.93	17.25	16.98	0.90
$\Delta G_{FG/RG}$	55.82	7.40	53.62	51.66	7.24
Ca^{2+}	73.22	14.08	73.18	74.37	16.51
Mg^{2+}	105.21	18.56	108.71	112.59	29.69
Y^{3+}	5.47	1.99	5.83	4.83	2.36
K^{+}	487.27	103.39	503.24	2739.25	15083.85
Na^{+}	3763.19	1700.23	4207.47	231054.69	871979.16
Gu^{+}	173.06	21.10	175.53	180.93	34.66
$r0_{nn}$	67.52	3.67	63.63	63.71	3.78
$r0_{pp}$	54.44	9.32	56.04	56.04	8.44
$r0_{pn}$	91.46	5.95	84.23	84.48	6.10
$r0_{FG/RG}$	25.97	4.53	27.31	27.72	4.71
erval	46.53	2.10	48.36	48.30	1.81

Table S3. Stickers and spacers model parameters.

Supplementary references

1. Nott, T. J. *et al.* Phase Transition of a Disordered Nuage Protein Generates Environmentally Responsive Membraneless Organelles. *Mol. Cell* **57**, 936–947 (2015).
2. Brady, J. P. *et al.* Structural and hydrodynamic properties of an intrinsically disordered region of a germ cell-specific protein on phase separation. *Proc. Natl. Acad. Sci. U. S. A.* **114**, E8194–E8203 (2017).
3. Millard, P. S. *et al.* IDDomainSpotter: Compositional bias reveals domains in long disordered protein regions—Insights from transcription factors. *Protein Sci.* **29**, 169–183 (2020).
4. Tamiola, K., Acar, B. & Mulder, F. A. A. Sequence-specific random coil chemical shifts of intrinsically disordered proteins. *J. Am. Chem. Soc.* **132**, 18000–18003 (2010).
5. Stothard, P. The sequence manipulation suite: JavaScript programs for analyzing and formatting protein and DNA sequences. *Biotechniques* **28**, (2000).
