# Supplementary material for: Ion binding with charge inversion combined with screening modulates DEAD box helicase phase transitions
Source: Cell Rep. 2023 Nov 18;42(11):113375. doi: 10.1016/j.celrep.2023.113375 (PMC10935546; doi:10.1016/j.celrep.2023.113375)
Supplement: Document S2. Article plus supplemental information [file mmc5.pdf]

Cell Reports

Article

Ion binding with charge inversion combined with screening modulates DEAD box helicase phase transitions

Graphical abstract

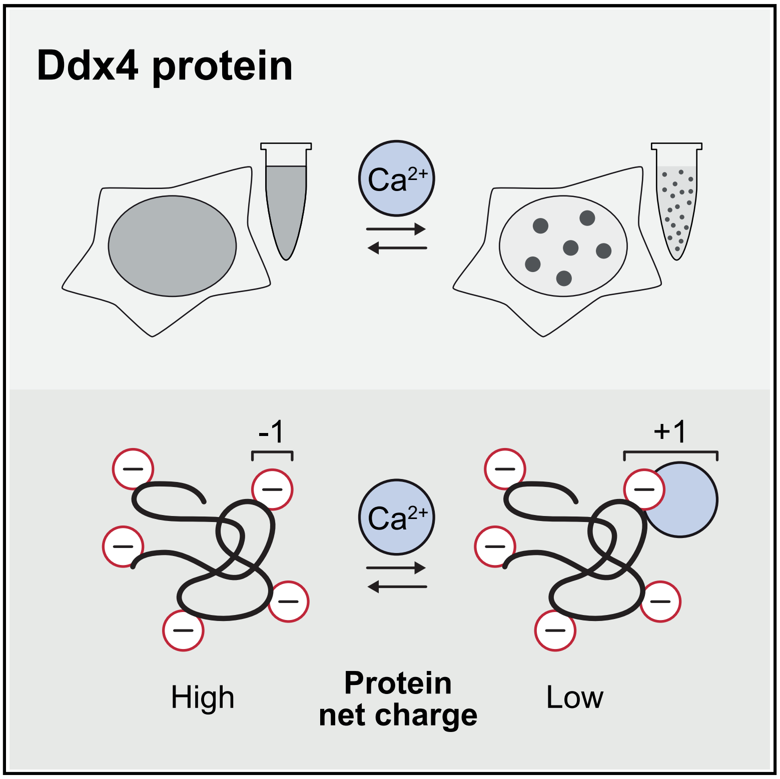

Highlights

- Multivalent cations promote Ddx4 phase transitions
- Ca^{2+} binds with low affinity to negatively charged residues in disordered regions
- Ca^{2+} binding lowers Ddx4 protein net charge, favoring phase separation
- Ca^{2+} binding alters condensate stability, dynamics, interfaces, and partitioning

Authors

Michael D. Crabtree, Jack Holland, Arvind S. Pillai, ..., Christina Redfield, Andrew J. Baldwin, Timothy J. Nott

Correspondence

andrew.baldwin@chem.ox.ac.uk (A.J.B.), tim.nott@kcl.ac.uk (T.J.N.)

In brief

Analyzing a family of model biomolecular condensates derived from Ddx4 and Ddx3 proteins over a range of solution conditions, Crabtree et al. show how a balance of repulsive and attractive electrostatic interactions regulate emergent condensate properties, and how these interactions can be tuned by signaling ions such as Ca^{2+}.

Crabtree et al., 2023, Cell Reports *42*, 113375
November 28, 2023 © 2023 The Authors.
https://doi.org/10.1016/j.celrep.2023.113375

CellPress

Cell Reports

CellPress
OPEN ACCESS

Article

Ion binding with charge inversion combined with screening modulates DEAD box helicase phase transitions

Michael D. Crabtree,[1] Jack Holland,[1] Arvind S. Pillai,[2] Purnima S. Kompella,[1] Leon Babl,[1] Noah N. Turner,[1] James T. Eaton,[3,6] Georg K.A. Hochberg,[4,5] Dirk G.A.L. Aarts,[3] Christina Redfield,[1] Andrew J. Baldwin,[3,6,*] and Timothy J. Nott[1,7,*]

[1]Department of Biochemistry, University of Oxford, South Parks Road, Oxford OX1 3QU, UK
[2]Institute for Protein Design, University of Washington, Seattle, WA 98195, USA
[3]Department of Chemistry, Physical & Theoretical Chemistry Laboratory, University of Oxford, Oxford OX1 3QZ, UK
[4]Department of Chemistry, Philipps University Marburg, Hans-Meerwein-Straße 4, 35032 Marburg, Germany
[5]Center for Synthetic Microbiology, Philipps University Marburg, Karl-von-Frisch-Straße 14, 35032 Marburg, Germany
[6]Kavli Insititute of Nanoscience Discovery, Dorothy Crowfoot Hodgkin Building, Sherrington Rd, Oxford, OX1 3QU, UK
[7]Lead contact
*Correspondence: andrew.baldwin@chem.ox.ac.uk (A.J.B.), tim.nott@kcl.ac.uk (T.J.N.)
https://doi.org/10.1016/j.celrep.2023.113375

SUMMARY

Membraneless organelles, or biomolecular condensates, enable cells to compartmentalize material and processes into unique biochemical environments. While specific, attractive molecular interactions are known to stabilize biomolecular condensates, repulsive interactions, and the balance between these opposing forces, are largely unexplored. Here, we demonstrate that repulsive and attractive electrostatic interactions regulate condensate stability, internal mobility, interfaces, and selective partitioning of molecules both *in vitro* and in cells. We find that signaling ions, such as calcium, alter repulsions between model Ddx3 and Ddx4 condensate proteins by directly binding to negatively charged amino acid sidechains and effectively inverting their charge, in a manner fundamentally dissimilar to electrostatic screening. Using a polymerization model combined with generalized stickers and spacers, we accurately quantify and predict condensate stability over a wide range of pH, salt concentrations, and amino acid sequences. Our model provides a general quantitative treatment for understanding how charge and ions reversibly control condensate stability.

INTRODUCTION

Biomolecular condensates, or membraneless organelles, are functional cellular compartments of concentrated protein and nucleic acid that appear and disappear in response to specific environmental stimuli.[1–7] They are present in nearly all eukaryotic cells,[8] and condensates such as stress granules, nucleoli, and nuage rapidly form, dissolve, and change their material properties in response to small changes in parameters such as pH, temperature, and salt concentration.[9–11] Underlying the self-assembly and emergent properties of biomolecular condensates are regulated networks of many transient, individually weak molecular interactions.[12] A hierarchy of condensate-stabilizing interactions, largely between charged and aromatic amino acids, have been identified through the study of intrinsically disordered regions (IDRs) of model condensate proteins such as Ddx4,[13,14] FUS,[15] and hnRNPA1.[16,17]

External salt ions exert significant influence over transient interactions between charged moieties underlying condensate stability, and small changes in ion concentration can switch condensate formation on and off.[13] Specific ion concentrations regulate autophagosome initiation,[18] P-body stability,[19] and nuage/chromatoid body condensates change in size and shape after exposure to Ca^{2+} during spermatogenesis.[20] In the context of protein misfolding and aggregation diseases such as Parkinson's and Alzheimer's diseases, small changes in Ca^{2+} and Zn^{2+} concentration influence phase separation of α-synuclein (α-Syn) and tau, respectively,[21–23] and solution ions greatly alter the stability of amyloid fibrils composed of insulin.[24] In aqueous buffer, tri- and tetravalent metal ions can modulate phase separation and aggregation of both globular proteins[25] and DNA.[26] Although our understanding of individual interactions between amino acids that stabilize biomolecular condensates has developed substantially, the molecular mechanisms by which different ions modulate and tune these interactions remains relatively poorly understood.

A range of ions have prominent roles *in vivo*. Na^+, K^+, and Cl^- are abundant, monovalent intracellular ions that contribute significantly to intracellular ionic strength (~150 mM). By contrast, intracellular concentrations of divalent Ca^{2+}, Mg^{2+}, and Zn^{2+} ions are typically lower and time dependent, peaking at mM during signaling events.[27–29] These ions can act as

© 2023 The Authors.
This is an open access article under the CC BY license (http://creativecommons.org/licenses/by/4.0/).

second messengers, causing conformational changes and stabilization of protein structure, often via direct binding to specific arrangements of acidic amino acid sidechains. For example, a single Ca^{2+} ion stabilizes EF-hand motifs by cooperatively coordinating 6 sites within a αDxDxDGx$_5$Eα consensus sequence (where x is any amino acid and α is an alpha helix).[30] In IDRs, Excalibur-like motifs can bind Ca^{2+} ions using a sequence similar to the EF-hand loop (DxDxDGxxCE), or via condensed-charge motifs that carry repetitive negative charges (e.g., VAEEDEDDDG in NHE1 or NEVDEEEEEG in ProTα)[31] with K_d values of ~0.05–0.1 mM. How mono- and multivalent ions affect condensate IDRs without these types of sequence is less well characterized.

From a fundamental perspective, increasing the background ion concentration (ionic strength) should electrostatically "screen" the interactions between charged moieties, which could either disfavor condensate formation if electrostatics are driving the phase separation or favor condensate formation if electrostatic repulsions are inhibiting phase separation. Where the ionic strength is approximately 150 mM, these effects should be well modeled by the extended Debye-Hückel limiting law.[32] Here, screening depends predominantly on the charge on the ions and so cannot account for effects that are strongly ion specific. Moving beyond this, the empirical Hofmeister series provides a ranking for how individual ions are expected to, for example, alter the solubility of proteins, although the specific mechanism by which this is achieved remains unclear.[33] By contrast, in the context of structural biology, conserved sequence motifs such as EF hands, Excalibur-like motifs, or condensed-charge motifs explain how specific ions can bind with proteins.[27,31] However, these ion-binding motifs are typically not present in the IDRs of key condensate proteins that are, nonetheless, highly sensitive to specific ions.[2,4,21,34] Current models of IDR phase separation account for the presence of ions via simple Debye-Hückel screening terms[13,35,36] or are based on observations of Hofmeister series effects.[35,37] A mechanism that brings together these observations is essential to provide insight into how ions can interact with IDRs and influence condensate formation.

To address this, here we study the effects of the presence of a range of different ions on the phase separation of Ddx4 and Ddx3 proteins created with varying levels of overall charge. These evolutionarily related model condensate proteins are RNA-dependent DEAD box ATPases, and readily phase separate *in vitro* and in cells.[13,38] Ddx4 is an essential protein component of mammalian nuage/chromatoid body[39,40] and has been extensively studied via mutagenesis; condensates are stabilized predominantly by cation-π interactions between RG (Arg Gly) and FG (Phe Gly) residue motifs and a periodic pattern of grouped charged residues.[13,41] Ddx4 homologues such as Vasa and GLH1–4 are conserved components of germ cell specific condensates in *Drosophila* (pole plasm, nuage) and *Caenorhabditis elegans* (P granules).[42] Ddx3 and its homologues, such as Bel (*Drosophila*) and Laf-1 (*C. elegans*), are expressed in somatic and germ cells, are key components of cytoplasmic ribonucleoprotein (RNP) granules, and play essential roles in multiple steps of RNA metabolism.

By challenging this family of sequences with a set of ions varying in type, ionic strength, and charge at near physiological concentrations, while retaining the core features in the sequence that stabilize condensates, we show that phase separation responds strongly to multivalent cations such as Ca^{2+}, Mg^{2+}, and Y^{3+}. We show that these specific effects cannot be understood by Debye-Hückel theory alone[32]; whereas monovalent (1+) cations destabilize Ddx condensates, multivalent (2+, 3+, etc.) cations are strongly stabilizing. Noting the absence of well recognized ion-binding motifs in our sequences, we determine by means of electrophoresis and NMR, that multivalent cations nevertheless bind negatively charged residues in the chain non-cooperatively with modest K_d values of ~100 mM. Ion binding specifically alters the protein charge, where, for example, binding a Ca^{2+} or Mg^{2+} ion to a -1 carboxylic acid sidechain effectively inverts the charge on the sidechain and results in combined moiety of charge +1 (charge inversion), effectively inverting the charge at that site.

We develop a general quantitative model to explain these effects on phase separation and demonstrate its effectiveness on a large dataset of conditions and Ddx protein sequences. We show that although core "sticker" residues are responsible for driving condensate formation, condensate stability is strongly tuned by the charge carried on positive and negatively charged residues. The pairwise electrostatic interactions between the charged residues can be either attractive or repulsive, which are screened by Debye-Hückel effects.[12] But crucially, the balance of these can be predictably tuned by varying the pH, mutagenesis (adding/subtracting charged residues), and charge inversion following specific ion binding. Globally analyzing the model identifies a K_d for Ca^{2+} of ~100 mM, which is consistent with the nuclear magnetic resonance (NMR) measurements. By contrast, Y^{3+} is found to bind more tightly, with $K_d \sim 10$ mM. Because of the abundance of charged residues in the disordered protein sequences, effects of ions were seen orders of magnitude below these individual binding affinities. Most remarkably, in the Ddx4 cases studied here, optimally stable condensates are obtained when the chain carries a net positive charge of +13, whereas under physiological conditions, the chain is negative. Thus adding multivalent ions results in binding that lowers the negative charge carried by the protein, and favoring phase separation, where the further the net charge is from the optimal value, the more sensitive condensate formation is to small changes in ion concentration.

Taken together, our model provides a general framework that can quantitatively describe the effects of ions over a range of condensate forming IDRs whose net charges can be predictably modulated by mutation, posttranslational modification, and pH. Any given condensate will be stabilized by a specific set of interactions. Our model predicts that the specific numbers of additional positively and negatively charged sidechains together with their affinities for different ions will predictably modulate the condensate stability via binding and charge inversion. Moreover, we show that functional properties of Ddx condensates such as their wetting, internal mobility, and selection for specific molecules (partitioning), correlate with condensate stability, and so are strongly but predictably modulated by charge and ions. Consistent with these findings, we demonstrate that the net charge of this family of protein sequences has been evolutionarily conserved. By demonstrating the need to incorporate

CellPress
OPEN ACCESS

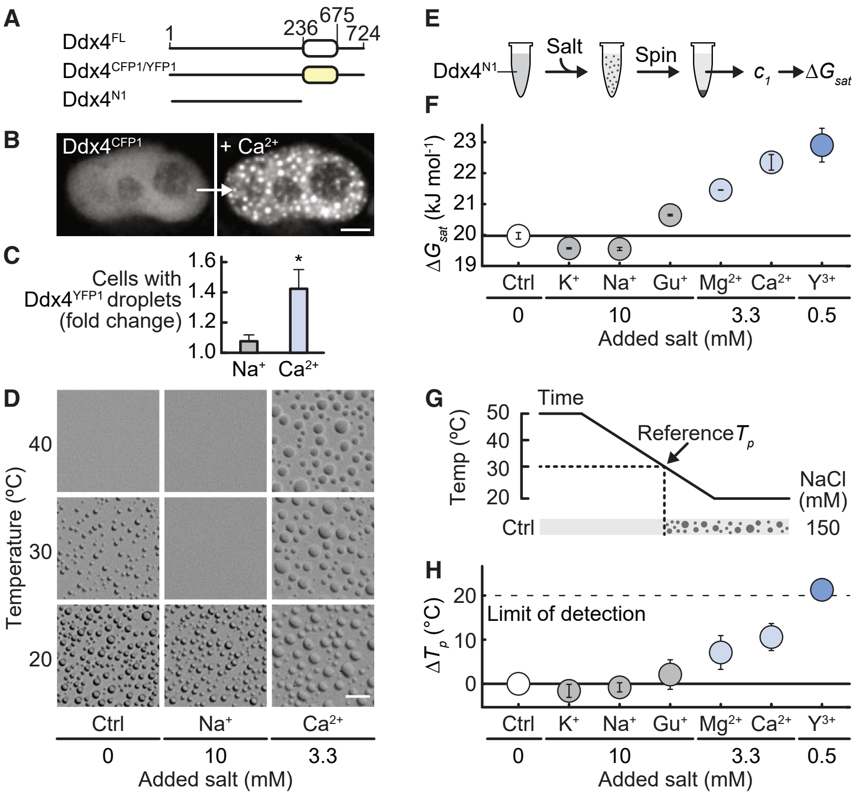

Figure 1. Cation valency modulates Ddx4^{N1} phase separation propensity

(A) Architecture of full-length human Ddx4 (Ddx4^{FL}), Ddx4^{CFP1} and Ddx4^{YFP1}, and Ddx4^{N1}. White oval indicates DEAD box helicase domain, yellow oval indicates mCerulean (CFP) or mCitrine (YFP), and black lines indicate IDRs. Amino acid numbers at domain boundaries are shown.

(B) Addition of 10 mM $CaCl_2$ to the cellular growth media induced formation of Ddx4^{CFP} condensates in live HeLa cells. Scale bar, 5 μm.

(C) Fold change of HeLa cells containing Ddx4^{YFP1} condensates following addition of 30 mM NaCl and 10 mM $CaCl_2$, relative to the control. Error bars indicate SD.

(D) Ddx4^{N1} condensates formed in a control sample at 30°C and with additional 10 mM NaCl and 3.3 mM $CaCl_2$. Scale bar, 20 μm.

(E) Schematic of ΔG_{sat} measurements. c_1 indicates the concentration of protein in the supernatant (dilute) phase.

(F) Additional monovalent cations (as chloride salts) decreased Ddx4^{N1} ΔG_{sat}, whereas addition of multivalent cations increased Ddx4^{N1} ΔG_{sat} relative to a control sample. Error bars indicate SD.

(G) Schematic of T_p measurements. T_p of the control sample is 30°C.

(H) Effect of additional monovalent and multivalent cations on Ddx4^{N1} T_p. Dotted line represents the maximum change in T_p that could be recorded. Error bars indicate SD.

"charge inversion" of sidechains on binding specific salt ions, our model provides a general means by which ion effects on condensate formation and emergent properties can be quantitatively understood.

RESULTS

Multivalent cations promote Ddx4 phase separation

Motivated by the observation that changes in intracellular Ca^{2+} ion concentrations in germ cells leads to morphological and functional changes in mammalian nuage/chromatoid bodies,[20] we initially examined how Ddx4 condensates responded to changes in Ca^{2+} ion concentration in cells. HeLa cells were transfected with a Ddx4 mimic, in which the DEAD box helicase domain was replaced by mCerulean (Ddx4^{CFP}), a fluorescent protein of similar size and charge to the DEAD box helicase domain, and we used microscopy to monitor Ddx4^{CFP} condensate formation (Figures 1A and 1B). Adding $CaCl_2$ to the media surrounding the cells promoted rapid formation of Ddx4^{CFP} condensates, in cells that both did, and did not previously contain Ddx4^{CFP} condensates (Figure S1A; Video S1).

To test whether this effect can be explained by simple changes in ionic strength, we developed an assay to quantitatively determine the fluorescence contrast and intensity coming from a cell transfected with fluorescent Ddx4 following brief incubation in media supplemented with different salts. Introducing 30 mM NaCl resulted in a modest, ~8% increase in the number of cells with Ddx4 condensates, whereas adding 10 mM $CaCl_2$, which matches the ionic strength of adding 30 mM NaCl, resulted in a 40% increase, suggesting that the effect is ion specific and cannot be explained by a change in media ionic strength alone (Figures 1C and S1B).

We pursued these observations *in vitro* under near physiological conditions of pH and ionic strength. A construct containing residues 1–236 of the N-terminal IDR of Ddx4 (Ddx4^{N1}) phase separates in a manner that is highly similar to the wild-type (WT).[13] The temperature at which a phase transition occurs, T_p, can be used to determine the relative stability of condensates.[13,43] A sample with 170 μM Ddx4^{N1} and 150 mM NaCl, had a transition point of 30°C. Adding 10 mM extra NaCl to this control sample reduced T_p marginally and disfavored phase separation (Figure 1D) in a manner consistent with screening attractive electrostatic interactions, as previously observed.[13] By contrast, addition of either ionic strength or concentration matched quantities of $CaCl_2$ (3.3 or 10 mM, respectively) to the control sample increased T_p, greatly promoting Ddx4^{N1} phase separation, just as we had seen in cells. Both *in vitro* and in cells, Ca^{2+}-induced condensate stabilization cannot be explained by electrostatic screening alone.

A self-assembly polymerization model describes condensate stability

T_p alone does not allow direct comparisons between the stability of condensates where the total protein concentration is varied between samples, as is necessary to do in this work. An attractive way to do this is via the Flory-Huggins interaction parameter (χ),

which directly measures the self-association interaction strength of a protein, that drives condensate formation. To calculate χ requires the dilute and condensed phase protein concentrations together with the overall condensate volume fraction.[13,41,44] These measurements remain technically challenging to perform on a large scale. An alternative measurement of stability has been proposed that requires only the concentration of protein in the dilute phase, c_{sat}.[15–17] Flory-Huggins theory applied to a two-state system predicts that at a constant temperature (where phase separation can occur) at a concentration that lies within the binodal limits, the concentration in the dilute phase is expected to be constant (and not vary with the total protein concentration). This implies that one can infer a free energy from $\Delta G_{FH} = -RT\ln(c_{sat})$. In general, Flory-Huggins theory can be used to derive an expression for this in the limit where protein in the protein poor phase is dilute (derivation in STAR Methods),

$$\Delta G_{FH} = RT\left(\chi\phi_1^b N_1(2-\phi_1^b) - \ln\left(\frac{\phi_1^b}{N_1 V_0}\right) - \phi_1^b(N_1-1)\right), \quad \text{(Equation 1)}$$

which depends on a complicated mixture of both interactions (χ), volume fraction of protein in the condensed phase (ϕ_1^b), the molar segment volume (V_0), and the number of monomeric subunits in the protein (N_1). In the limit where the condensed phase volume fraction is the same when comparing two such measurements,[17] an expression for the difference in free energy can be obtained that is proportional to the difference in χ and reflects the difference in stability between condensates, though this too is also proportional to the density of the condensed phase (STAR Methods). In this work, the volume fraction of the condensed phase is known to vary,[44] and so we cannot use this approach to directly map c_{sat} values onto a measure of stability. Moreover, in the presence of different salts and buffer ions, we do not expect to have, in general, a situation in which a simple 2-state Flory-Huggins approach will apply, as we anticipate different solute concentrations in the two phases.[36] Consistent with this physical view, we observed that the measured value of Ddx4^{N1} c_{sat} varies with the total protein concentration, where after crossing the lowest concentration binodal, the two scale roughly linearly, with a 20% increase in total concentration giving a 15% increase in c_{sat}. The reasons for this are not entirely clear. As noted, 2-state Flory-Huggins theory should lead to a c_{sat} value that is independent of c_{tot}. In principle, additional chemical factors such as salts mean that we no longer have a 2-state situation, and so the assumption that c_{sat} should not vary with c_{tot} becomes unsafe.

To make progress, in this work we propose an alternative approach to measure the thermodynamic stability of condensates on the basis of a generalized self-assembly scheme by Oosawa and Kasai.[45] In this model, free protein (c_1) can be considered in equilibrium with condensates (c_i, $i > 1$), where protein chains assemble in a successive manner where a mean field "average" association equilibrium constant K_a governs each successive step in the self-assembly pathway, allowing K_a to behave as though it is independent of condensate size (derivation in STAR Methods), leading to

$$K_a = \frac{1}{c_1} - \frac{1}{\sqrt{c_1 c_{tot}}}. \quad \text{(Equation 2)}$$

Oosawa and Kasai's approach allows us to define $\Delta G_{sat} = RT\ln K_a$, which is a measure of the stability of condensates[15–17] suitable for generalized comparisons. Defined in this way, an increasingly positive ΔG_{sat} reflects more stable condensates. Aggregation phenomena as diverse as actin filaments,[45] micelle formation,[46] chaperone oligomerization,[47] and amyloid fibril formation[48] are among the systems that have been characterized previously using this scheme. In the limit where condensates are highly stable, $c_{tot} \gg c_1$, then $K_a \sim \frac{1}{c_1}$, $c_1 = c_{sat}$, and the two approaches converge, $\Delta G_{sat} = \Delta G_{FH}$. The polymerization model is not suitable for generally analyzing phase separation, but nevertheless it is a convenient method that allows us to quantify approximate thermodynamic stabilities of condensates from measured values of c_{tot} and c_{sat}, and avoids the challenges associated with projecting potentially high dimensional phase diagrams on to two-state Flory-Huggins theory. Moreover, the physical intuition is extremely clear with the polymerization model, a stronger interaction between proteins leads to a larger value of K_a which yields a more positive value for ΔG_{sat}. The polymerization model presented here thus provides a method to roughly account for the variation in c_{sat} with c_{tot}, though we stress that the major conclusion of the paper, that we can accurately model ΔG by accounting for the effects stated, is the same whether or not we use ΔG_{FH} or ΔG_{sat}.

Armed with an expression that allows us to estimate the stability of condensates, ΔG_{sat} (Equation 3), we could quantitatively explore the effects on condensate stability of a wide range of cations and other conditions (Figures 1E–H). Importantly, the effects of adding $CaCl_2$ to Ddx4^{N1} were reflected by both a higher T_p and a higher ΔG_{sat}, revealing that both measures describe increased condensate stability.

We would have naively expected the effects of different ions on condensate stability to follow the Hofmeister series[35,37,49,50]: $K^+ < Na^+ < Mg^{2+} < Ca^{2+} < Gu^+$ (guanidinium [$CH_6N_3^+$]). Broadly speaking, the series for ΔG_{sat} roughly follows this order, with K^+ and Na^+ being slightly destabilizing and Mg^{2+} and Ca^{2+} stabilizing. Nevertheless Gu^+ was found to act more like Na^+ (Figure 1F). Although the Hofmeister series qualitatively rationalizes the effects of ions on protein structure, it does not directly translate to understand the stability of Ddx4^{N1} condensates. Finally, we determined using both ΔG_{sat} and T_p measurements that the Y^{3+} ion is significantly more condensate stabilizing than either Ca^{2+} or Mg^{2+} (Figures 1F–1H and S1C).

Electrostatic screening has previously well explained lowered condensate stability because of the additions of monovalent ions such as Na^+ and Cl^-[13,17,35,36] In search of a plausible mechanism to explain our data, we hypothesized that the enhanced stability due to modest quantities of Mg^{2+}, Ca^{2+}, and Y^{3+} suggested specific binding. However, a bioinformatic analysis revealed no established consensus sequences for binding of either ion in the Ddx4 sequence.

CellPress
OPEN ACCESS

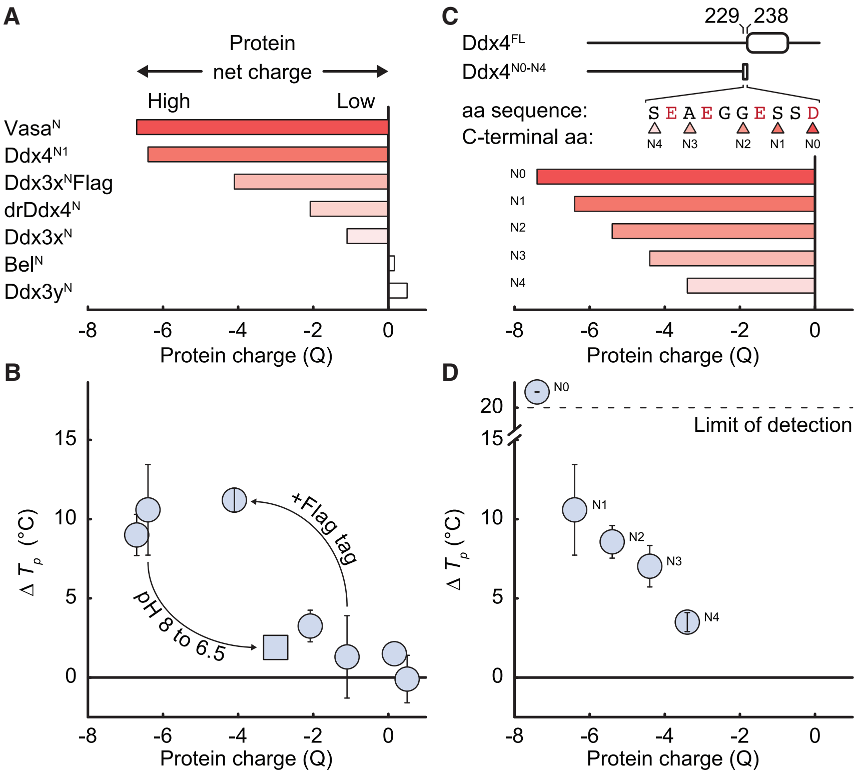

Figure 2. The ability of Ca^{2+} ions to promote Ddx4 N-terminal IDR protein phase separation depends on protein net charge

(A) Net charge (*Q*) at pH 8 for the N-terminal IDRs of Ddx4 homologues and paralogs.

(B) Ca^{2+} ions promote phase separation in a manner that depends on protein net charge rather than amino acid sequence. ΔT_p is the difference in transition temperature upon adding ionic strength-matched amounts of $CaCl_2$ (3.3 mM) or NaCl (10 mM) to samples containing 150 mM NaCl and 20 mM Tris (pH 8) (or PIPES [pH 6.5]). Square marker indicates the result of altering the net charge of Ddx4^{N1} from −6.4 to −3.1 by changing pH from 8.0 to 6.5. Error bars indicate SD.

(C) Net charge at pH 8 for Ddx4$^{N0\text{-}N4}$ charge series. C-terminal amino acid sequences are shown for the different charge series constructs. Amino acid numbers encompassing mutations in the Ddx4$^{N0\text{-}N4}$ charge series (229–238) relative to Ddx4^{FL} are indicated.

(D) Ca^{2+} ions promote phase separation of the Ddx4$^{N0\text{-}N4}$ charge series in a manner that depends on protein net charge. ΔT_p obtained as described in (B) legend. Dotted line represents the maximum change in T_p that could be recorded. Error bars indicate SD.

Ca^{2+} promotes phase separation of more negatively charged Ddx4 and Ddx3 proteins

To ascertain the generality of the ion effects observed for Ddx4^{N1}, we developed a library of related sequences comprising the N-terminal IDRs of Ddx4 (Figure 2A; Table S1) including homologues (*Drosophila* Vasa^{N} and zebrafish drDdx4^{N}) and somatically expressed paralogs (human Ddx3x^{N}, Ddx3y^{N}, and *Drosophila* Bel^{N}). Although the evolutionary relationships are obtained from aligning sequences of the DEAD box helicase domains, the N-terminal domains are of low sequence identity (Table S2). However, an algorithm we derived previously[13] that examines the number and patterning of FG and RG residue pairs finds these sequences highly similar, predicting that all should phase separate. We experimentally confirmed that this was indeed the case. Most notably, phase separation of the Ddx4 homologue Vasa^{N} was promoted by Ca^{2+}, whereas the paralogs (Ddx3x^{N}, Ddx3y^{N}, and Bel^{N}) were relatively unaffected under these conditions (Figure 2B). Interestingly, drDdx4^{N} showed a more Ddx3x-like response to Ca^{2+}.

Although none of the proteins possessed a predicted Ca^{2+}-binding motif, inspection of the sequences revealed that the highly negatively charged protein sequences were the most sensitive to Ca^{2+} ions, whereas the neutrally charged sequences carried no sensitivity. We tested the significance of this observation by adding an acidic FLAG tag at the C-terminus of Ddx3x^{N}. This changed Ddx3x^{N} net charge (at pH 8.0) from −1.1 to −4.1 and resulted in re-introducing Ca^{2+} ion-induced phase separation, as predicted (Figure 2B).

To further investigate the effects of the net charge, we systematically varied the charge on Ddx4^{N1} by reducing the pH from 8 to 6.5, which was expected to change the charge from −6.4 to −3.1.[51] Consistently, this lowered the sensitivity of phase separation of Ddx4^{N1} to Ca^{2+} addition (Figure 2B). Finally, we noted a cluster of negatively charged amino acids at the C-terminal end of Ddx4^{N1}. This allowed us to engineer a further set of 5 sequences, Ddx4$^{N4\text{-}N0}$, whose charges span the range −3.4 to −7.4 (residues 1–229 to 1–238) at pH 8 and so make Ddx4 sequences that are less charged and more "Ddx3 like" (Figure 2C). As with the homologues and paralogs, the sensitivity of Ddx4$^{N4\text{-}N0}$ condensate formation to Ca^{2+} was determined to be highly predictable, decreasing with decreasing total net charge on the protein chain (Figure 2D).

Net charge of Ddx4 and Ddx3 proteins is evolutionarily conserved

As the net charge on the protein chain predicts its sensitivity to multivalent ions *in vitro*, we sought to explore this characteristic within the evolution of Ddx4 and Ddx3 proteins. Our phylogenetic analysis revealed that the ancestor of Ddx4 and Ddx3 proteins may have shared a net charge slightly above neutral, with Ddx4 evolving toward a net negative charge after its divergence from Ddx3 via a gene duplication that occurred early in animal evolution (Figure S2). Despite clear differences in overall net charge, Ddx4 and Ddx3 protein clades appear to have retained relatively similar levels of condensate-stabilizing amino acids such as F (Phe) and R (Arg). Taken together, our analysis

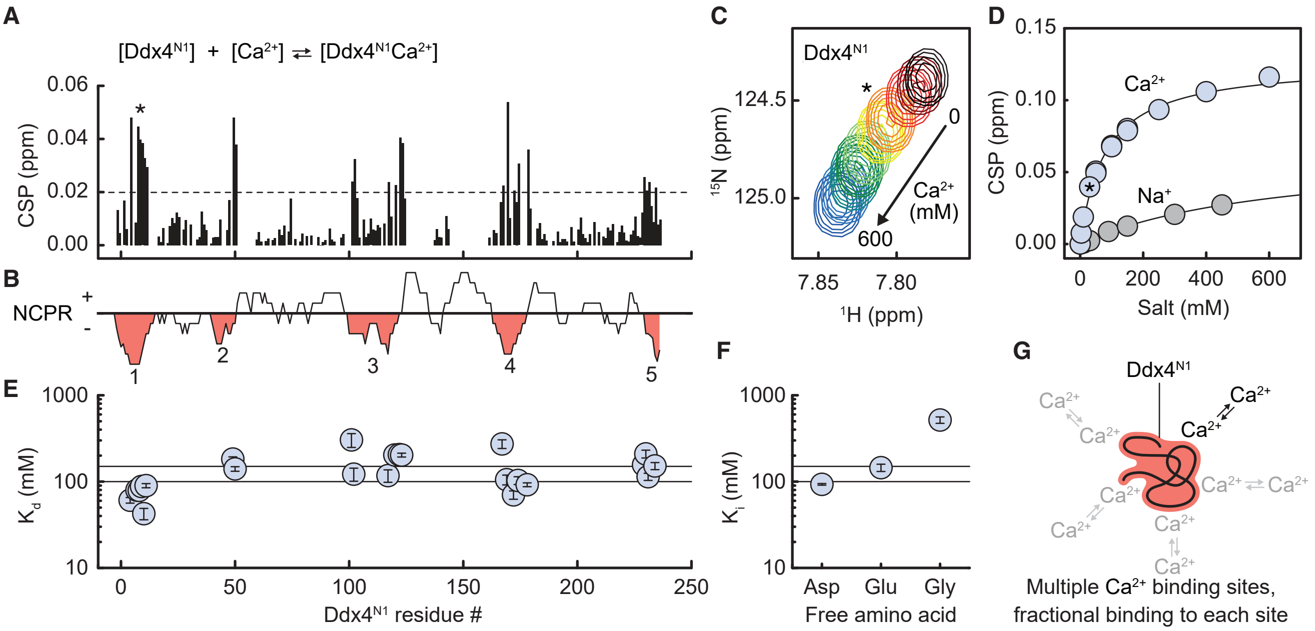

Figure 3. Ca^{2+} binds to individual negatively charged sidechains

(A) Backbone amide chemical shift perturbations (CSPs) for $Ddx4^{N1}$ upon addition of 30 mM $CaCl_2$. Dotted line indicates a CSP of 0.02 ppm. Amino acid (aa) numbering as in (E). A (Ala) 8 (asterisk) is next to E (Glu) 7 and E (Glu) 9.

(B) Net charge per residue (NCPR; 10 aa sliding window, 1 aa step) for $Ddx4^{N1}$ indicating 5 regions of local negative charge (red, numbered 1–5).

(C) Backbone chemical shifts of A (Ala) 8 as $CaCl_2$ is titrated from 0 to 600 mM. Asterisk indicates the chemical shift at 30 mM added $CaCl_2$ (orange).

(D) CSPs of A (Ala) 8 for 0–450 mM additional NaCl (gray points) and 0–600 mM additional $CaCl_2$ (blue points). Black lines indicate the fit to the data. Asterisk indicates the chemical shift at 30 mM added $CaCl_2$.

(E) K_d values for residues with a CSP at 30 mM $CaCl_2$ greater than 0.02. Error bars indicate the estimated error for the best-fit value using a bootstrap method. Horizontal lines are drawn at 100 and 150 mM K_d.

(F) Affinity of free D (Asp), E (Glu), and G (Gly) amino acids for Ca^{2+}. Values are expressed in terms of K_i as affinities were derived from a competition binding experiment. Error bars indicate the estimated error for the best-fit value.

(G) Ca^{2+} ions bind weakly to multiple sites on the $Ddx4^{N1}$ protein chain.

indicated that Ddx4 has evolved and conserved a net negative charge across a vast range of animal lineages, suggesting functional importance.

Calcium ions bind with low affinity to negatively charged residues in disordered proteins

Having established that Ca^{2+} promotes phase separation of a wide range of Ddx protein sequences in a manner that depends on the net charge of the sequence, we sought to determine if we can measure binding of ions under conditions in which phase separation does not occur. NMR spectroscopy is a natural choice for this measurement, as the protein chains are expected to be highly disordered and the affinities of ions for individual residues are likely to be weak ($K_d >$ mM). Following assignment of $Ddx4^{N1}$ backbone amide resonances, we observed in BEST-TROSY HN experiments that titration of $CaCl_2$ into solutions of ^{15}N-labeled $Ddx4^{N1}$ led to changes in backbone amide chemical shifts across the length of the protein, indicating binding (Figures 3A and S3A). Most notably, the largest changes clustered to the 5 regions that contain negatively charged residues (D [Asp] and E [Glu]; Figures 3B and S3B), and chemical shifts of the backbone suggests slight movement toward more disordered secondary structure in the affected regions (Figure S3G).

For the 37 residues where the variation in chemical shift with Ca^{2+} was significant, a residue specific K_d was obtained through fitting the deviation in chemical shift to a model that assumes the binding to be "fast" (Figures 3C and 3D). K_d values varied from 82 to 174 mM for the 5 negatively charged regions, and clustered to give a central distribution of 146 ± 41 mM (Figure 3E) suggesting modest variation in the affinity with position in the sequence. Adding NaCl at concentrations that match the ionic strength of $CaCl_2$ revealed similar changes, though the magnitude of the effect was substantially reduced to the extent that it was not possible to reliably obtain a K_d, suggesting a value >1,000 mM (Figures 3D, S3C, and S3D).

We repeated the $CaCl_2$ titration with $Ddx4^{N0}$, $Ddx4^{N4}$, and $Ddx4^{N1}$ at pH 8, finding that although the total number of binding sites varied with the net charge on the protein (−0.1 to −6.4, $Ddx4^{N4}$, $Ddx4^{N0}$, and $Ddx4^{N1}$ [pH 6.5 and 8]), the Ca^{2+} affinity for each residue was highly similar for all sequences tested (Figure S3E). This affinity is markedly weaker than expected for typical Ca^{2+} binding sites such as EF-hand motifs, C2 domains, annexins,[30] Excalibur-like, or condensed-charge motifs,[31] expected in the range 0.01–1 mM. The binding affinities measured

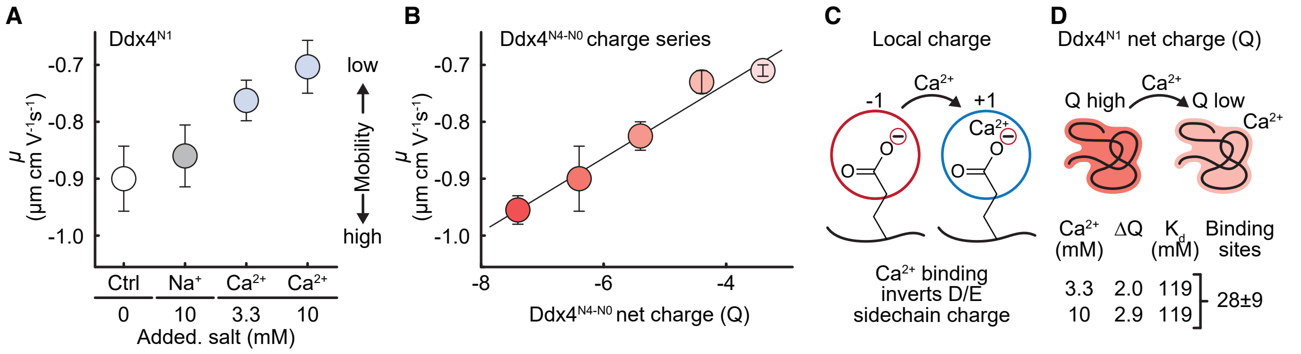

Figure 4. Charge reversal explains the correlation between divalent ion effects on Ddx4$^{N0\text{-}N4}$ phase separation and protein net charge

(A) Addition of $CaCl_2$ lowers the electrophoretic mobility (μ) of Ddx4^{N1} toward 0. Error bars indicate the SD.

(B) Standard curve of μ created with the Ddx4$^{N0\text{-}N4}$ charge series. Error bars indicate the SD.

(C) Upon binding, the valency mismatch between a divalent Ca^{2+} ion and a negatively charged D (Asp) or E (Glu) sidechain leads to a reversal in charge from negative (−1) to positive (+1). This change in sidechain charge upon Ca^{2+} ion binding is independent of the starting net charge of the protein.

(D) Binding of Ca^{2+} ions to Ddx4^{N1} lowers the overall protein net charge. 28 ± 9 individual binding sites (negatively charged amino acid sidechains) are required to explain the change in charge observed in ELS experiments.

were instead consistent with Ca^{2+} binding to the sidechains of individual D (Asp) (K_i = 93 ± 2 mM) and E (Glu) (K_i = 145 ± 12 mM) residues (Figures 3F and S3F).

Taken together, NMR spectroscopy reveals that Ca^{2+} ions specifically bind the negatively charged regions of Ddx4 (Figure 3G), and that the data are well explained by assuming that each negatively charged residue behaves independently (non-cooperatively), with a K_d value consistent with that expected for free, negatively charged amino acids.

Multivalent ion binding alters Ddx4 protein net charge

Having established that Ca^{2+} ions specifically bind to residues in negatively charged regions of Ddx4, we sought to ascertain the effects of this on the net charge of the protein. Electrophoretic light scattering (ELS) determines the steady-state mobility of molecules (or particles) in a liquid under an applied electric field. As with the NMR experiments, ELS was performed using dilute solutions under conditions in which the protein does not phase separate. Compared with a control sample containing 150 mM NaCl, addition of 10 mM extra NaCl did not significantly change the mobility of freely diffusing Ddx4^{N1} protein molecules (Figure 4A). By contrast, addition of 3.3 mM $CaCl_2$ (matching the ionic strength) significantly reduced the mobility, consistent with Ca^{2+} ions weakly binding the protein, lowering its average net charge.

Control measurements were repeated for the Ddx4$^{N4\text{-}N0}$ charge series to ascertain the variation in mobility with net charge over the range −3.4 to −7.4 (Figure 4B). These values were compared with the mobility of Ddx4^{N1} with 3.3 and 10 mM $CaCl_2$, allowing us to estimate that the net charge on the chain was reduced by 2 and 2.9 units, respectively. By assuming that the binding of one Ca^{2+} ion to a negatively charged sidechain, such as D (Asp) or E (Glu), changes the apparent charge of the sidechain from −1 to +1 (Figure 4C), and using a K_d for Ca^{2+} ion binding of 119 ± 37 mM (average of free D [Asp] and E [Glu]; Figures 3F and S3F), we estimated that 28 ± 9 binding sites are required to explain the change in protein charge (Figure 4D). This range is consistent with the 24 D (Asp) and E (Glu) residues present in the negatively charged regions identified by NMR to bind Ca^{2+} ions.

Taken together, under physiologically relevant salt concentrations, we expect the negatively charged regions of Ddx proteins to at most be only fractionally bound to Ca^{2+} ions. Yet each Ca^{2+} ion bound is sufficient to locally invert the charge, which in turn alters the average net charge on the chain which tunes the stability of condensates. We next sought to understand quantitatively how variation in total charge can affect the stability of the condensates.

Optimally stable Ddx4^{N1} condensates occur when the chain carries +13 charge

To quantitatively explore how the total charge affects the stability of the condensates, we first measured ΔG_{sat} for Ddx4^{N1} as a function of pH, in the range 7.6–3.2 (Figure 5A). Over this pH range, the ionization state of C (Cys), H (His), D (Asp), and E (Glu) residue sidechains and the N and C termini all change, causing the expected net charge to vary from −5.2 to +31.4 (Figure 5B). ΔG_{sat} followed a parabolic form with a maximum (most stable condensates) occurring when the chain carries a charge of +13 (pH 4.5) (Figure 5C). On either side of this protein charge, phase separation is disfavored, indicated by the decreasing ΔG_{sat}.

This observation can be quantitatively understood using a modified "stickers and spacers" model.[17] In such a model, regions within a polymer chain are designated "stickers," whose associations drive condensate formation. For Ddx4^{N1} we expect this to be driven largely by the pairing of F (Phe) and R (Arg) residues that are adjacent to at least one G (Gly) or S (Ser).[13,41] Having explored this in detail previously, in what follows we do not vary the composition of these "core" condensate forming residues and this contribution to the stability will be a constant. We introduce two specific electrostatic stickers reflecting positively (R (Arg) and K (Lys); p), and negatively (D [Asp] or E [Glu]; n) charged residues and allow both pairs of FGs/RGs and p/n residues to interact favorably and pairs of positively or negatively charged residues (p/p and n/n) to repel each other.

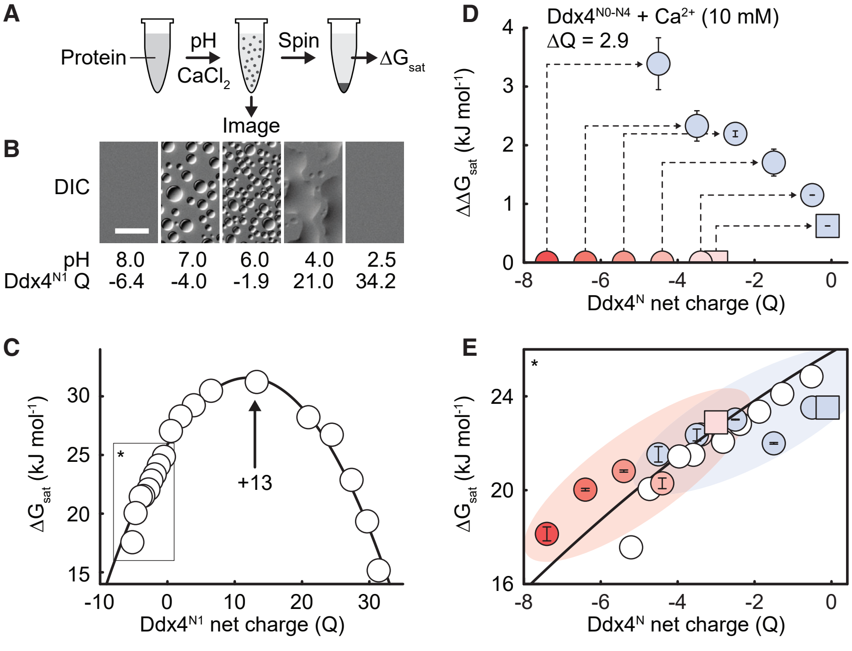

Figure 5. Effect of pH, mutation, and mono- and multivalent cations on Ddx4 phase separation

(A) Schematic of how Ddx4^{N1} ΔG_{sat} measurements and images were obtained.

(B) Differential interference contrast (DIC) images of Ddx4^{N1} condensates at indicated pH and protein charge. Ddx4^{N1} phase separation was not observed in samples at pH 8.0 or 2.5. Scale bar, 20 μm.

(C) ΔG_{sat} of Ddx4^{N1} as a function of net charge (Q) adjusted by pH. Black line is a fit to Equation 3 that includes both the Ddx4^{N1} pH series data (white points) and the Ddx4^{N0-N4} charge mutant (pH 8) data in (E) (red-pink data points). Black rectangle and asterisk indicate the area plotted in (E).

(D) $\Delta\Delta G_{sat}$ of Ddx4^{N0-N4} following addition of 10 mM CaCl$_2$ (blue data points) diminishes with decreasing Q. Binding of Ca^{2+} ions to Ddx4^{N0-N4} changes the protein net charge by a fixed amount (here, +2.9 with 10 mM CaCl$_2$). Initial charge of Ddx4^{N0-N4} (pH 8; round) and Ddx4^{N1} pH 6.5 (square) is indicated with red-pink points. Dashed lines indicate of effects of adding CaCl$_2$ on $\Delta\Delta G_{sat}$ and ΔQ for Ddx4^{N0-N4} proteins. Error bars indicate the propagated SD.

(E) Binding of Ca^{2+} ions to Ddx4^{N0-N4} changes Q by a fixed amount (D legend), causing the charge-adjusted ΔG_{sat} data (blue) to align well with the fitted line (black). Red-pink data points as in (D). White data points as in (C). Error bars indicate the SD. Data points without error bars represent single measurements.

Taking a linear free energy relationship on the basis of counting the pairwise interactions between sidechains in a mean-field manner, we expect

$$\Delta G_{sat} = x_{FG}x_{RG}\Delta G_{FG/RG} + \frac{1}{2}x_p(x_p - 1)\Delta G_{pp} + \frac{1}{2}x_n(x_n - 1)\Delta G_{nn} + x_n x_p \Delta G_{pn}, \quad \text{(Equation 3)}$$

where x is a count of a single "sticker" type. Although x_{FG} and x_{RG} will not substantially vary in what follows, we anticipate that the number of positively and negatively charged residues (x_p, x_n) will be a predictable function of pH following the Henderson-Hasselbalch equation[51] and that the net charge of the protein, Q, is the difference in positive and negative residues, $x_p - x_n$. In the pH range (7.6–3.2), where all the variation in charge can be attributed to variation in x_n only, the model can be re-written simply in parabolic form, $\Delta G_{sat} = \gamma Q^2 + \beta Q + \alpha$, where α, β, γ are simply constants related to the various free energies (derivation in STAR Methods).

This model has several interesting features. Assuming that x_p is constant, the model predicts the condensates will be maximally stable when the chain carries the following charge (STAR Methods)

$$Q_{opt} = -\frac{\beta}{2\gamma} = x_p\left(1 + \frac{\Delta G_{pn}}{\Delta G_{nn}}\right) - \frac{1}{2}. \quad \text{(Equation 4)}$$

For the Ddx4^{N} proteins, this charge is +13. As $x_p = 32$, $\frac{\Delta G_{pn}}{\Delta G_{nn}} = -0.57$ and we ascertain that individual repulsions between negatively charged residues are stronger than the attractions. Similarly, we note that the condensates are maximally stable when the pH is approximately equal to the pK_a of the acidic residues (pH 4.3). Our model allows us to calculate that this occurs when

$$\frac{\Delta G_{pn}}{\Delta G_{nn}} = \frac{1 - x_n^0}{2x_p}, \quad \text{(Equation 5)}$$

where the superscript 0 indicates the total count of residues before pH adjustment. As $x_n^0 = 36$, the ratio is -0.55, which is remarkably consistent with the estimate obtained from the peak stability occurring for +13 protein chains. From these two quite simple observations, our model, provides significant physical insight into how condensate stability is affected by adjacent charged residues.

The major interaction that stabilizes Ddx condensates, the cation-π interaction, carries a positive charge, thus perhaps it is not surprising that the optimal stability occurs when the chain is positive. Nevertheless, it is not intuitive that optimal stability should occur between chains that naively should strongly repel each other. As the FG/RG motifs are distributed across the chain, adding in a relatively modest number of negatively charged residues introduces additional electrostatic attractions that help stabilize the condensates. But add too many and condensate stability starts to decrease as repulsions begin to dominate (Figure 5C).

Sidechain charge inversion quantitatively rationalizes condensate stability

We used this framework to re-examine condensate stability following Ca^{2+} addition and its charge reversal effects. We first

CellPress
OPEN ACCESS

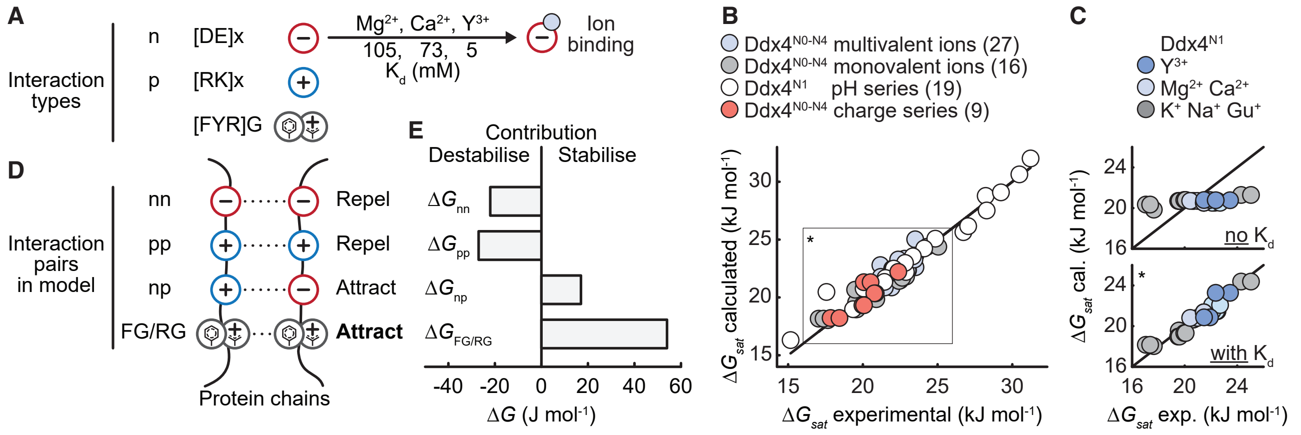

Figure 6. Modeling of electrostatic interactions confirms multivalent cations invert sidechain anion charge

(A) Three types of interactors are sufficient to explain the experimental ΔG_{sat} data: negatively charged amino acids (D [Asp] or E [Glu]; denoted n), positively charged amino acids (K [Lys] or R [Arg]; denoted p), and amino acids capable of engaging in cation-π interactions (F [Phe], Y [Tyr], or R [Arg] bounded on at least one side by a G [Gly]). x denotes any amino acid.

(B) Correlation between experimental and calculated ΔG_{sat} values. Calculated Ddx4^{N} ΔG_{sat} values were in good agreement with the experimental values (RMS error = 0.75 kJ mol^{-1}) and predicted K_d values that matched the affinities calculated by NMR. Black line shows x = y. Black rectangle and asterisk indicates the area plotted for Ddx4^{N1} without and with ion binding K_d values in (C).

(C) When the effects of ion binding were excluded, the model was not able to capture the trends in the ion data, shown for Ddx4^{N1}. Asterisk relates to the area plotted in (B).

(D) Our model includes Debye-Hückel screening, ion binding, repulsion between like-charged amino acids (nn, pp), attraction between oppositely charged amino acids (np), and cation-π attractions (FG/RG) to quantitively describe the effects of mutation, pH, and ions on Ddx4 condensate stability.

(E) Contributions of individual pairwise interactions to condensate stability.

defined a change in free energy on adding Ca^{2+}, $\Delta\Delta G_{sat} = \Delta G_{sat}^{10\text{ mM }Ca^{2+}} - \Delta G_{sat}^{0\text{ mM }Ca^{2+}}$. For Ddx4$^{N0\text{-}N4}$, $\Delta\Delta G_{sat}$ decreases with decreasing net charge on the chain. This suggests that we can understand the sensitivity to Ca^{2+} addition by looking at how rapidly ΔG_{sat} changes with charge (Figure 5D). Under conditions in which $d\Delta G_{sat}/dQ$ is large, we expect condensate stability is highly sensitive to small changes in total charge, and hence highly Ca^{2+} ion concentration dependent. By contrast, when condensates are at or near their optimal stability, the free energy is less sensitive to small changes in charge.

Consistent with this, when we adjust the total charge on the Ddx4$^{N0\text{-}N4}$ protein chains upon addition of Ca^{2+} by the values obtained experimentally from the ELS measurements, we find that the ΔG_{sat} values with Ca^{2+} are in close agreement with those obtained from the pH titration of Ddx4^{N1} (Figure 5E). This suggests that we can quantitatively rationalize condensate stabilities purely through considering the effects of salt ion binding, and charge inversion.

We can model this by modifying Equation 3 to allow each negatively charged residue to bind a cation with an ion-specific K_d value (Figure 6A). When bound, in addition to decreasing the number of available negatively charged residues, the number of positively charged moieties is also increased in a manner that depends on the charge of the cation. Finally, following from previous work we note that all 4 free energies in Equation 3 are electrostatic in origin, and should in principle be screened according to Debye-Hückel theory. This provides a simple global model that should allow us to predict all ΔG_{sat} values for Ddx4 chains provided we know the protein sequence charge, pH, and salt concentrations (Figure S4A).

Globally, we have 71 ΔG_{sat} measurements to account for with this model (Figure 6B). The pK_a values of the amino acids are assumed to be constant and equal to standard values, a reasonable assumption for a disordered protein, and so we have only to fit 4 free energies extrapolated to zero salt ($\Delta G_{FG/RG}$, ΔG_{nn}, ΔG_{pp}, and ΔG_{pn}), 4 characteristic distances that re-scale the screening for each interaction type, the relative dielectric, and 6 K_d values, one for each salt ion systematically varied in the dataset (K^+, Na^+, Gu^+, Mg^{2+}, Ca^{2+}, and Y^{3+}): 15 parameters in total (Table S3). When the effects of ion binding were excluded, our model was not able to capture the trends in the ion data (Figure 6C) and when the effects of screening were omitted, the destabilization of condensates with increasing NaCl concentration was not captured, and so it is clearly essential that we include these aspects.

Most remarkably, the optimized model is in outstanding agreement with the measured data. The measured values of ΔG_{sat} fall in the range 16–32 kJ mol^{-1}, and the root-mean-square (RMS) error of the fit is 0.75 kJ mol^{-1} (Figure 6B). We determine that, as expected, $\Delta G_{FG/RG}$ is the dominant term, acting to stabilize the condensates (Figures 6D and 6E). ΔG_{nn} and ΔG_{pp} are similar in magnitude and repulsive as expected, and the attractive electrostatic interactions ΔG_{pn} was the smallest, as expected from the earlier analysis. The cation-π interactions associated with $\Delta G_{FG/RG}$ were the most sensitive to screening, and the relative dielectric was fitted to 47 ± 2, a value consistent with previous measurements (45 ± 13).[13]

This allows us to interpret the stability of the condensates in a straightforward fashion. Although paired FG/RG interactions placed within characteristic patterns stabilize Ddx condensates,

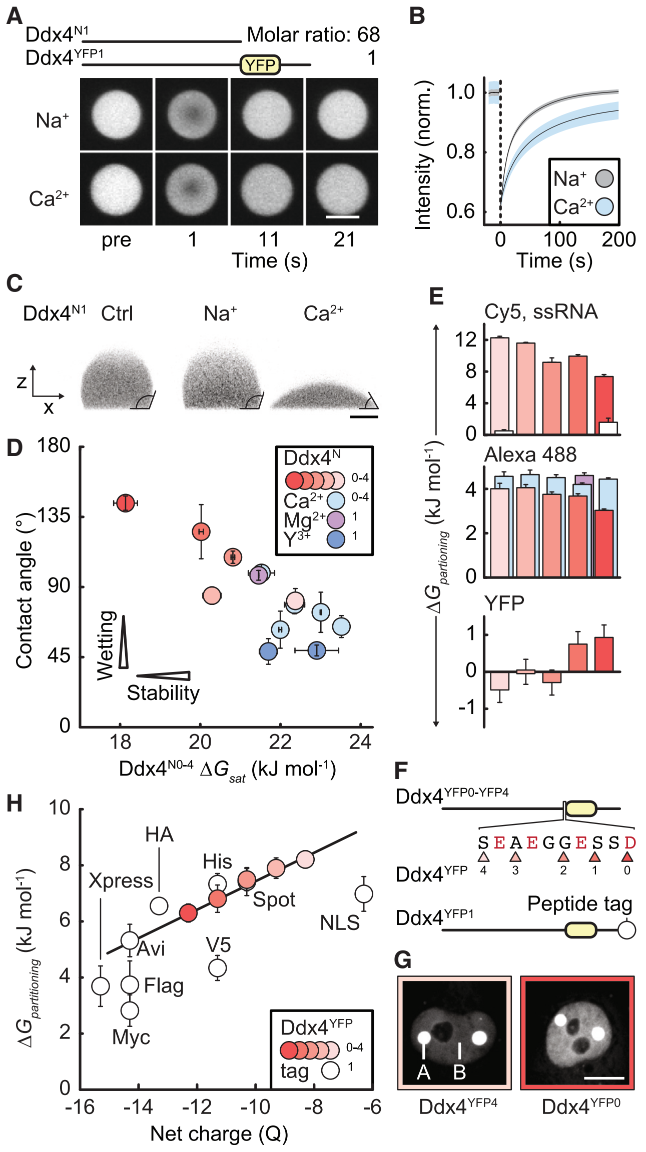

Figure 7. Ddx4^{N0-N4} biomolecular condensates are modulated by ion binding and condensate protein charge

(A) FRAP experiment showing the signal originating from Ddx4^{YFP1} (in condensates otherwise composed of Ddx4^{N1}) pre- and post-bleach. Molar ratio of the two proteins is indicated. Scale bar 5 μm.

(B) Mean fits of Ddx4^{YFP1} FRAP in the presence of additional NaCl (30 mM) or $CaCl_2$ (10 mM). Errors indicate SEM.

(C) Images (xz plane) of Ddx4^{N1} condensates sitting on siliconized glass coverslips. Addition of Ca^{2+} ions (10 mM), but not Na^+ ions (30 mM), changed Ddx4^{N1} condensate contact angle from >90° to <90°. Contrast is derived from Alexa 488 fluorescence. Scale bar 5 μm.

(D) Contact angles of Ddx4^{N0-N4} condensates in the absence and presence of additional multivalent ions ($CaCl_2$ and $MgCl_2$ (10 mM), YCl_3 (0.5 mM)), indicated in box inset. Error bars represent SD.

(E) Partitioning of free Cy5 dye (white bars) and Cy5-labeled 24-mer single-stranded RNA (ssRNA) (top), free Alexa 488 dye (middle), and YFP (bottom) into Ddx4^{N0-N4} charge series condensates. Error bars represent SD. Same color scheme as in (D).

(F) Ddx4^{N0-N4} charge series implemented in the context of Ddx4^{YFP} (Ddx4$^{YFP0-YFP4}$). Triangles (and corresponding construct numbers) indicate the C-terminal residue of the Ddx4 sequence immediately preceding mCitrine. White circle indicates C-terminal peptide tag.

(G) Fluorescence originating from Ddx4^{YFP4} and Ddx4^{YFP0} in HeLa cell nuclei (A, condensates; B, nucleoplasm). Scale bar, 10 μm.

(H) Stability of Ddx4$^{YFP0-YFP4}$ and Ddx4^{YFP1} with C-terminal peptide tags. Error bars indicate SD.

repulsions between pairs of negatively charged residues are destabilizing, but general attractions between negative and positive residues are "slightly" stabilizing. Cations can weakly and transiently bind the negatively charged residues in a specific manner, following the trend Y^{3+} (5 ± 2 mM) > Ca^{2+} (73 ± 14 mM) > Mg^{2+} (105 ± 19 mM) > Gu^+ (173 ± 21 mM) > K^+ (487 ± 103 mM) > Na^+ (1,000 mM), where the value for Ca^{2+} is consistent with the average value measured using NMR on the dilute phase. This quantitative ranking for Ddx4^{N1} is fundamentally dissimilar to the Hofmeister series and instead reflects the specific, independent interaction strengths of charged residues within the protein, and the free ions. In the case where repulsions between negatively charged residues dominate, such as when the chain is negatively charged, binding di- and trivalent cations stabilizes condensates. In the case where the repulsions between negatively charged residues is modest, when the overall chain is close to neutral, then the condensate stability becomes largely invariant to cation binding (Figures 2B, 2D, and 5D). More generally, adding all salts such as NaCl will act to screen all interactions which are fundamentally electrostatic in character, which tends to destabilize Ddx condensates.

Overall, multivalent cations can have either dramatic effects on condensate stability (e.g., Ddx4), or modest (e.g., Ddx3) in a manner that simply depends on the net charge of the chain under the conditions studied reflecting binding and sidechain charge inversion, and the sensitivity to changes in salt, and this can be quantitatively captured using our model.

Ion binding alters functional condensate properties *in vitro*

Having established that binding of multivalent cations can greatly influence condensate stability, we sought to ascertain if they can also alter functional characteristics such as internal mobility, shape/size, and the partitioning of different types of molecules. First, to assess if Ca^{2+} alters the mobility of Ddx4 protein chains inside the condensates, we used fluorescence recovery after photobleaching (FRAP) to monitor Ddx4^{YFP1} inside Ddx4^{N1} condensates. Adding ionic strength matched amounts of NaCl (30 mM) or $CaCl_2$ (10 mM) had fluorescence in both cases recovering to near the initial level (99% ± 2% and 94% ± 3%, respectively), demonstrating that condensates had retained their liquid-like nature in the presence of additional cations (Figure 7A). Quantitative analysis of FRAP curves revealed both cases were well described by a biexponential model, with fast and slow phases diffusing at similar rates following the addition of equivalent amounts of NaCl or $CaCl_2$ (Figures 7B and S5A). The populations of fast and slow phases were similar following Na^+ ion addition. By contrast, Ca^{2+} ion addition significantly increased the proportion of slow diffusing proteins, consistent with a model where Ca^{2+} ions can form transient bridges between protein chains inside the condensates, decreasing their

mobility. The mobility of protein chains within the condensates is therefore strongly affected by modest quantities of Ca^{2+}.

The shape and size of biomolecular condensates, as well as how they interact with other surfaces within the cell, will be affected by their surface tension.[3,52–54] We determined the relative strength of the surface tensions of Ddx4 and Ddx3x condensates and how these are affected by salts by comparing the contact angle of condensates on a siliconized glass surface. Under conditions in which Ddx4^{N1} formed beaded droplets, the addition of $CaCl_2$ caused near complete wetting of the surface, whereas the addition of NaCl had no effect (Figure 7C). Notably, over the entire dataset incorporating different proteins (Ddx4^{N4-N0}, Ddx3x^{N}, and Ddx3x^{N}FLAG) and different ion additives (Na^{+}, Mg^{2+}, Ca^{2+}, and Y^{3+}), condensate contact angle was found to correlate with ΔG_{sat}, suggesting a relationship between condensate stability and the condensate surface (Figures 7D and S5B).

Condensates are known to differentially partition biomolecules.[13,14,55–57] We hypothesized that condensate protein charge would affect uptake of different molecules, as estimated from the differences in fluorescence.[14] Cy5, an electrically neutral dye showed little preference for condensates formed of either Ddx4^{N0} (charge of -7.4) or Ddx4^{N4} (charge of -3.4) (Figure 7E, top, white bars). By contrast, Alexa 488 with a net charge of -3, was absorbed more strongly by the more neutral Ddx4^{N4} than Ddx4^{N0} condensates (Figure 7E middle). Consistent with this, adding Ca^{2+} greatly increased the absorption of the dye, with the largest effect observed for the condensates formed from Ddx4^{N0}. Going further, a 24-mer single-stranded RNA fused to Cy5 was strongly absorbed by Ddx4^{N0} condensates and enhanced further by Ddx4^{N4} condensates (Figure 7E, top). By contrast, all Ddx4 condensates were relatively unselective for YFP, with little variation (Figure 7E, bottom). Clearly, condensate protein charge, as well as different salt ions, can affect the uptake into condensates in a manner that depends on the biomolecule.

Adding Ca^{2+} to Ddx4^{N1} altered both the wetting of condensates and the degree to which they absorbed Alex 488, which could be monitored in real time (Figure S5C; Video S2). Subsequent removal of Ca^{2+} ions, via the addition of EDTA, immediately returned the Ddx4^{N1} condensates to their initial state. The speed and reversibility of the reaction suggested that Ca^{2+} ions could freely enter and leave the Ddx4^{N1} condensates, consistent with weakly binding to the Ddx4 protein chain, and that the condensate interior remained dynamic and liquid-like during this process, consistent with FRAP measurements. Simulations have indicated that ions should enter condensates to maintain electroneutrality,[58] but our data indicate that there can be additional, direct binding between proteins and ions that alter emergent condensate properties.

Protein charge determines condensate stability in cells

Finally, having established that protein charge alters the stability and emergent properties of proteinaceous condensates *in vitro*, we sought to ascertain its impact on condensates in cells. Using a series of fluorescent proteins based on our Ddx4^{N0-N4} charge series (Figure 7F), condensate stability in cells varied markedly and predictably with protein charge as it had done *in vitro* (Figures 7G and 7H). Moreover, adding short peptide tags (6–15 residues) to the C-terminal end of Ddx4^{YFP1} also altered condensate stability in a predictable fashion, with condensate stability decreasing when the tags increased the negative charge (Figures 7F, 7H, and S5D–S5E). These results show that condensate stability can be predictably manipulated in cells via changes in overall protein charge, regardless of whether the changes are made at the end or in the middle of the protein sequence.

DISCUSSION

We have extensively manipulated the charge on a series of phase separating Ddx proteins both biochemically, through creating a series of minimally edited sequences (Ddx4^{N0-N4}), by altering the pH and by adding various salts, *in vitro* and in cells. We derive a modified "stickers and spacers" model that quantitatively accounts for how the "core stability" of the condensate is tuned by charged residues and modulated by ion binding that crucially is highly sensitive to the total charge carried by a protein chain. We also show that condensate wetting, internal mobility, and biomolecule partitioning reflect the stability of the condensates and so are also strongly affected by protein net charge. Consistent with its biophysical importance, we demonstrate that the net charge of these sequences has been evolutionarily conserved.

The stability of a wide range of condensates formed from both proteins and nucleic acids are pH, and hence charge, sensitive.[15,17,59] Our model provides a quantitative means to understand fundamental properties of the interactions holding condensates together from such measurements. We might assume that to get maximally stable condensates, we should just engineer only the "good" sticker interactions, which for Ddx4 are predominantly between FG and RG residue motifs. However, consistent with observations of FUS,[15] our quantitative analysis shows that condensate stability can be enhanced by including a small number of additional positively and negatively charged residues. But if a sequence has "too many" of any one charge type, repulsions will dominate, and the resulting condensates become less stable. When designing and tuning the properties of condensates, these factors can be quantitatively accounted for using our model, once the free energies have been determined for a sequence of interest. The model can also be used qualitatively, allowing cellular observations describing how condensates respond to ions to be explained in terms of the net charge on the condensate proteins.

Ddx4^{N1} condensates are most stable when the protein carries a net charge of +13. Interestingly, hnRNPA1 condensates are most stable at a protein charge of $\sim$+3.[17] These are counterintuitive findings, as naively we might expect optimal attraction to occur for neutral chains. However, as the key inter-residue interactions that govern Ddx4 and hnRNPA1 condensate stability are driven by cation-π pairs (with a combined charge of +1), this finding becomes less surprising. Our model allows calculation of the optimal charge by accounting for the balance of competing electrostatic interactions. Optimal stability of Ddx4^{N1} condensates occurs when the pH matches the pK_a of the negatively charged residues. For this to happen, according to our model, the magnitude of the repulsion strength needs to exceed the magnitude of the attraction, $\Delta G_{nn} > \Delta G_{pn}$ (Equation 3). We would

not expect this result to hold for all condensates, with the specific values likely to vary with the patterning of core stabilizing residues in the sequences under study.

By examining how condensate formation responds to changes in protein charge and the presence of different salt ions, our work demonstrates an unexpected but powerful mechanism by which low concentrations of multivalent cations can influence condensate stability.[35] Although the effects of physiological concentrations of monovalent ions like Na^+ and K^+ are well accounted for by Debye-Hückel screening, multivalent ions such as Ca^{2+}, Mg^{2+}, and Y^{3+} act through binding individual charged residues, which effectively inverts their charge and hence modifies the overall charge on the protein chain. Although the specific K_d values that govern the interactions are relatively weak (~100 mM for Ca^{2+}; Figure 3), as individually charged residues behave independently and there are many in the protein sequence, ion concentrations of ~1 mM can be sufficient to raise the charge by 1 or 2 units, which under physiological conditions is sufficient to effectively switch condensates "on."

Our model also reveals features in protein sequences that render a condensate sensitive to pH and salt ions. The further away a condensate is from its optimal stability, the larger $d\Delta G_{sat}/dQ$, and so the more sensitive it will be to changes in pH, salt, and the presence of other charged species. The charge encoded in specific primary condensate constituents could therefore confer either robustness against environmental changes or sensitivity to them. Near neutrally charged Ddx3 is expressed in both somatic and germline cells, whereas Ddx4 proteins are only expressed in the germline and is highly negative. It is interesting to note from our evolutionary analysis that the two proteins diverged at the point where organisms developed germ cells. As Ddx4 proteins generally carry more negative charge than Ddx3 proteins, they are further from their optimal stability ($d\Delta G_{sat}/dQ$ is large), rendering them more sensitive to external factors such as time dependent Ca^{2+} concentrations that occur during spermatogenesis or in mature sperm cells.[20,60,61] Similarly, we also anticipate the net charge on the protein chain and external ions driving variations in condensate shape, size, internal mobility, and partitioning of biomolecules in cells.

This mechanism is highly similar to the "charge inversion" mechanism of DNA and RNA condensation in the presence of multivalent cations such as spermine (+4), Ca^{2+}/Mg^{2+}, or short polycationic peptides (e.g., $[RGRGG]_5$ and $[KGKGG]_5$), where the cation associates with the phosphate backbone to lower the net charge carried by the nucleic acid.[26,62–64] Analogous observations have been made of the effect of ATP (−4) on a highly positively charged fragment of CAPRIN1.[65] In these cases, once repulsion between nucleic acid or protein molecules is sufficiently low, condensation occurs and so our model is suitable to quantitatively explain these effects.

Multivalent ions such as Ca^{2+} and Mg^{2+} have many known roles in cellular biology[27,66,67] and changes in divalent ion concentrations are associated with human disease.[22,23,68,69] Here we establish for IDRs particularly a salt ion binding-induced charge inversion mechanism that can dramatically alter the net charge on a protein, and hence critical properties for condensates such as stability, internal mobility, interaction with surfaces and their ability to selectively absorb biomolecules. Our model accounts for all these features and can be widely applied to dissect the various competing roles of the interactions described here, and we anticipate its utility in teasing apart biological interactions and in the rational design of synthetic condensates. Multivalent ions are important signaling ligands in cells, and so together these results reveal an additional mechanism by which protein-protein interactions and biomolecular condensate formation and properties can be tuned.

Limitations of the study

Here, we have studied how mono-, di-, and trivalent cations interact with the IDRs of Ddx4 and Ddx3 proteins. All cations were introduced as chloride salts. We did not consider the role of different anions or their valency on Ddx protein phase separation. Studying more diverse, non-evolutionarily related, phase separating sequences would enable greater testing of the generality of the model. Using full-length proteins that incorporate the DEAD box helicase domains could have potentially led to additional insight.

In this work, we use a free energy expression derived from Oosawa and Kasai's polymerization model. This is a generalized self-assembly model that has seen extensive use in biophysics for many decades to measure the stability of aggregates. We could alternatively have used $\Delta G_{FH} = -RT\ln(c_{sat})$ in our work as described in the text, and the conclusions, trends, and global model selection would be unchanged (apart from some modest variation in fitting parameters). As discussed in the text and STAR Methods, neither approach is completely satisfactory for describing condensate formation, but both provide a means to assess condensate stability from simple concentration measures suitable to interpret a series of related measurements. A rigorous derivation of ΔG_{FH} from Flory-Huggins theory is presented in the STAR Methods. As empirically we see variation in c_{sat} with c_{tot} we prefer to use the polymerization model as our thermodynamic barometer here.

STAR★METHODS

Detailed methods are provided in the online version of this paper and include the following:

- KEY RESOURCES TABLE
- RESOURCE AVAILABILITY
  - Lead contact
  - Materials availability
  - Data and code availability
- EXPERIMENTAL MODEL AND STUDY PARTICIPANT DETAILS
  - Cells and culture conditions
- METHOD DETAILS
  - Protein expression and purification
  - Protein concentration determination
  - Transition temperature (T_p) determination
  - Net charge calculation
  - Sequence alignment and phylogenetic inference
  - Sequence identity and similarity
  - NMR
  - Affinity determination of free amino acids for calcium

- ○ Electrophoretic light scattering
- ○ Confocal fluorescence microscopy of Ddx4 condensates *in vitro*
- ○ Real-time droplet imaging
- ○ *In vitro* partitioning
- ○ Fluorescence recovery after photo bleaching (FRAP)
- ○ Contact angle determination
- ○ HeLa cell culture and transfection
- ○ Imaging fixed HeLa cells
- ○ Live cell imaging
- ○ In cell determination of condensate stability of Ddx4^{YFP0-4} charge series and Ddx4^{YFP1} C-terminally tagged constructs
- ○ Flow cytometry
- ○ Generalised condensate stability model
- ○ Stability model
- ○ Condensate stability versus charge
- ○ Optimal pH
- ○ Optimal condensate stability
- ○ Applying the model to Ddx data in a global optimisation
- ○ Ionic complexes in both free and bound states?
- ○ Derivation of ΔG_{FH} from Flory Huggins theory

● QUANTIFICATION AND STATISTICAL ANALYSIS

SUPPLEMENTAL INFORMATION

Supplemental information can be found online at https://doi.org/10.1016/j.celrep.2023.113375.

ACKNOWLEDGMENTS

M.D.C. and T.J.N. thank Micron Oxford for microscopy support and M. Maj for support with flow cytometry experiments. T.J.N. thanks F. Barr and members of the Barr group for the gift of HeLa cells and support with tissue culture. We thank M. Krishnan for discussions on charge-inversion and seeing the significance of this mechanism in our data. We thank the Engineering and Physical Sciences Research Council (EPSRC) (EP/R029849/1) and the Wellcome Institutional Strategic Support Fund, John Fell Fund, and Edward Penley Abraham Cephalosporin Fund at the University of Oxford for funding the 950 MHz NMR facility. A.J.B. is supported by ERC grant 101002859. For the purpose of open access, the author has applied a CC BY public copyright license to any author-accepted manuscript version arising from this submission. Funding for this work came from a Sir Henry Dale Fellowship jointly funded by the Wellcome Trust and the Royal Society (grant 202320/Z/16/Z) awarded to T.J.N. T.J.N. and M.D.C. thank New College, Oxford, for support. L.B. thanks Studienstiftung des deutschen Volkes, and J.H. acknowledges the Synthetic Biology Centre for Doctoral Training (EPSRC grant EP/L016494/1) for support. G.K.A.H. is support by the Max Planck Society.

AUTHOR CONTRIBUTIONS

Conceptualization, M.D.C. and T.J.N.; methodology, M.D.C. and A.J.B.; resources, J.T.E., D.G.A.L.A., and C.R.; data curation, C.R.; investigation, M.D.C., J.H., A.S.P., P.S.K., L.B., N.N.T., and T.J.N.; formal analysis, M.D.C., J.H., A.S.P., A.J.B., and T.J.N.; visualization, M.D.C., A.J.B., and T.J.N.; writing – original draft, M.D.C.; writing – review & editing, J.H., G.K.A.H., D.G.A.L.A., A.J.B., and T.J.N.; funding acquisition, T.J.N.; supervision, T.J.N.

DECLARATION OF INTERESTS

The authors declare no competing interests.

Received: March 10, 2023
Revised: July 14, 2023
Accepted: October 18, 2023
Published: November 18, 2023

REFERENCES

1. Han, T.W., Kato, M., Xie, S., Wu, L.C., Mirzaei, H., Pei, J., Chen, M., Xie, Y., Allen, J., Xiao, G., and McKnight, S.L. (2012). Cell-free formation of RNA granules: Bound RNAs identify features and components of cellular assemblies. Cell *149*, 768–779.
2. Jain, S., Wheeler, J.R., Walters, R.W., Agrawal, A., Barsic, A., and Parker, R. (2016). ATPase-Modulated Stress Granules Contain a Diverse Proteome and Substructure. Cell *164*, 487–498.
3. Feric, M., Vaidya, N., Harmon, T.S., Mitrea, D.M., Zhu, L., Richardson, T.M., Kriwacki, R.W., Pappu, R.V., and Brangwynne, C.P. (2016). Coexisting Liquid Phases Underlie Nucleolar Subcompartments. Cell *165*, 1686–1697.
4. Stenström, L., Mahdessian, D., Gnann, C., Cesnik, A.J., Ouyang, W., Leonetti, M.D., Uhlén, M., Cuylen-Haering, S., Thul, P.J., and Lundberg, E. (2020). Mapping the nucleolar proteome reveals a spatiotemporal organization related to intrinsic protein disorder. Mol. Syst. Biol. *16*, e9469.
5. Dodson, A.E., and Kennedy, S. (2020). Phase Separation in Germ Cells and Development. Dev. Cell *55*, 4–17.
6. Yang, P., Mathieu, C., Kolaitis, R.M., Zhang, P., Messing, J., Yurtsever, U., Yang, Z., Wu, J., Li, Y., Pan, Q., et al. (2020). G3BP1 Is a Tunable Switch that Triggers Phase Separation to Assemble Stress Granules. Cell *181*, 325–345.e28.
7. Marmor-Kollet, H., Siany, A., Kedersha, N., Knafo, N., Rivkin, N., Danino, Y.M., Moens, T.G., Olender, T., Sheban, D., Cohen, N., et al. (2020). Spatiotemporal Proteomic Analysis of Stress Granule Disassembly Using APEX Reveals Regulation by SUMOylation and Links to ALS Pathogenesis. Mol. Cell *80*, 876–891.e6.
8. Lafontaine, D.L.J., Riback, J.A., Bascetin, R., and Brangwynne, C.P. (2021). The nucleolus as a multiphase liquid condensate. Nat. Rev. Mol. Cell Biol. *22*, 165–182.
9. Iserman, C., Desroches Altamirano, C., Jegers, C., Friedrich, U., Zarin, T., Fritsch, A.W., Mittasch, M., Domingues, A., Hersemann, L., Jahnel, M., et al. (2020). Condensation of Ded1p Promotes a Translational Switch from Housekeeping to Stress Protein Production. Cell *181*, 818–831.e19.
10. Zhu, P., Lister, C., and Dean, C. (2021). Cold-induced Arabidopsis FRIGIDA nuclear condensates for FLC repression. Nat *599*, 657–661.
11. Watanabe, K., Morishita, K., Zhou, X., Shiizaki, S., Uchiyama, Y., Koike, M., Naguro, I., and Ichijo, H. (2021). Cells recognize osmotic stress through liquid–liquid phase separation lubricated with poly(ADP-ribose). Nat. Commun. *12*, 1353.
12. Choi, J.M., Holehouse, A.S., and Pappu, R.V. (2020). Physical Principles Underlying the Complex Biology of Intracellular Phase Transitions. Annu. Rev. Biophys. *49*, 107–133.
13. Nott, T.J., Petsalaki, E., Farber, P., Jervis, D., Fussner, E., Plochowietz, A., Craggs, T.D., Bazett-Jones, D.P., Pawson, T., Forman-Kay, J.D., and Baldwin, A.J. (2015). Phase Transition of a Disordered Nuage Protein Generates Environmentally Responsive Membraneless Organelles. Mol. Cell *57*, 936–947.
14. Nott, T.J., Craggs, T.D., and Baldwin, A.J. (2016). Membraneless organelles can melt nucleic acid duplexes and act as biomolecular filters. Nat. Chem. *8*, 569–575.
15. Wang, J., Choi, J.M., Holehouse, A.S., Lee, H.O., Zhang, X., Jahnel, M., Maharana, S., Lemaitre, R., Pozniakovsky, A., Drechsel, D., et al. (2018). A Molecular Grammar Governing the Driving Forces for Phase Separation of Prion-like RNA Binding Proteins. Cell *174*, 688–699.e16.
16. Martin, E.W., Holehouse, A.S., Peran, I., Farag, M., Incicco, J.J., Bremer, A., Grace, C.R., Soranno, A., Pappu, R.V., and Mittag, T. (2020). Valence

and patterning of aromatic residues determine the phase behavior of prion-like domains. Science *367*, 694–699.

17. Bremer, A., Farag, M., Borcherds, W.M., Peran, I., Martin, E.W., Pappu, R.V., and Mittag, T. (2022). Deciphering how naturally occurring sequence features impact the phase behaviours of disordered prion-like domains. Nat. Chem. *14*, 196–207.
18. Zheng, Q., Chen, Y., Chen, D., Zhao, H., Feng, Y., Meng, Q., Zhao, Y., and Zhang, H. (2022). Calcium transients on the ER surface trigger liquid-liquid phase separation of FIP200 to specify autophagosome initiation sites. Cell *185*, 4082–4098.e22. https://doi.org/10.1016/J.CELL.2022.09.001.
19. Sankaranarayanan, M., Emenecker, R.J., Wilby, E.L., Jahnel, M., Trussina, I.R.E.A., Wayland, M., Alberti, S., Holehouse, A.S., and Weil, T.T. (2021). Adaptable P body physical states differentially regulate bicoid mRNA storage during early Drosophila development. Dev. Cell *56*, 2886–2901.e6.
20. Andonov, M.D., and Chaldakov, G.N. (1989). Morphological evidence for calcium storage in the chromatoid body of rat spermatids. Exp *45*, 377–378.
21. Huang, S., Xu, B., and Liu, Y. (2022). Calcium promotes α-synuclein liquid-liquid phase separation to accelerate amyloid aggregation. Biochem. Biophys. Res. Commun. *603*, 13–20.
22. Singh, V., Xu, L., Boyko, S., Surewicz, K., and Surewicz, W.K. (2020). Zinc promotes liquid–liquid phase separation of tau protein. J. Biol. Chem. *295*, 5850–5856.
23. Yatoui, D., Tsvetkov, P.O., La Rocca, R., Baksheeva, V.E., Allegro, D., Breuzard, G., Ferracci, G., Byrne, D., and Devred, F. (2022). Binding of two zinc ions promotes liquid-liquid phase separation of Tau. Int. J. Biol. Macromol. *223*, 1223–1229.
24. Shammas, S.L., Knowles, T.P.J., Baldwin, A.J., Macphee, C.E., Welland, M.E., Dobson, C.M., and Devlin, G.L. (2011). Perturbation of the Stability of Amyloid Fibrils through Alteration of Electrostatic Interactions. Biophys. J. *100*, 2783–2791.
25. Matsarskaia, O., Roosen-Runge, F., Lotze, G., Möller, J., Mariani, A., Zhang, F., and Schreiber, F. (2018). Tuning phase transitions of aqueous protein solutions by multivalent cations. Phys. Chem. Chem. Phys. *20*, 27214–27225.
26. Besteman, K., Van Eijk, K., and Lemay, S.G. (2007). Charge inversion accompanies DNA condensation by multivalent ions. Nat. Phys. *3*, 641–644.
27. Matsarskaia, O., Roosen-Runge, F., and Schreiber, F. (2020). Multivalent ions and biomolecules: Attempting a comprehensive perspective. ChemPhysChem *21*, 1742–1767.
28. Bagur, R., and Hajnóczky, G. (2017). Intracellular Ca^{2+} Sensing: Its Role in Calcium Homeostasis and Signaling. Mol. Cell *66*, 780–788.
29. Maeshima, K., Matsuda, T., Shindo, Y., Imamura, H., Tamura, S., Imai, R., Kawakami, S., Nagashima, R., Soga, T., Noji, H., et al. (2018). A Transient Rise in Free Mg2+ Ions Released from ATP-Mg Hydrolysis Contributes to Mitotic Chromosome Condensation. Curr. Biol. *28*, 444–451.e6.
30. Rigden, D.J., and Galperin, M.Y. (2004). The DxDxDG motif for calcium binding: Multiple structural contexts and implications for evolution. J. Mol. Biol. *343*, 971–984.
31. Newcombe, E.A., Fernandes, C.B., Lundsgaard, J.E., Brakti, I., Lindorff-Larsen, K., Langkilde, A.E., Skriver, K., and Kragelund, B.B. (2021). Insight into Calcium-Binding Motifs of Intrinsically Disordered Proteins. Biomolecules *11*, 1173.
32. Outhwaite, C.W. (1969). Extension of the Debye–Hückel Theory of Electrolyte Solutions. J. Chem. Phys. *50*, 2277–2288.
33. Gregory, K.P., Elliott, G.R., Robertson, H., Kumar, A., Wanless, E.J., Webber, G.B., Craig, V.S.J., Andersson, G.G., and Page, A.J. (2022). Understanding specific ion effects and the Hofmeister series. Phys. Chem. Chem. Phys. *24*, 12682–12718.
34. Millar, S.R., Huang, J.Q., Schreiber, K.J., Tsai, Y.C., Won, J., Zhang, J., Moses, A.M., and Youn, J.Y. (2023). A New Phase of Networking: The Molecular Composition and Regulatory Dynamics of Mammalian Stress Granules. Chem. Rev. *123*, 9036–9064.
35. Krainer, G., Welsh, T.J., Joseph, J.A., Espinosa, J.R., Wittmann, S., de Csilléry, E., Sridhar, A., Toprakcioglu, Z., Gudiškytė, G., Czekalska, M.A., et al. (2021). Reentrant liquid condensate phase of proteins is stabilized by hydrophobic and non-ionic interactions. Nat. Commun. *12*, 1085.
36. Dignon, G.L., Zheng, W., Kim, Y.C., Best, R.B., and Mittal, J. (2018). Sequence determinants of protein phase behavior from a coarse-grained model. PLoS Comput. Biol. *14*, e1005941.
37. Murthy, A.C., Dignon, G.L., Kan, Y., Zerze, G.H., Parekh, S.H., Mittal, J., and Fawzi, N.L. (2019). Molecular interactions underlying liquid−liquid phase separation of the FUS low-complexity domain. Nat. Struct. Mol. Biol. *26*, 637–648.
38. Hondele, M., Sachdev, R., Heinrich, S., Wang, J., Vallotton, P., Fontoura, B.M.A., and Weis, K. (2019). DEAD-box ATPases are global regulators of phase-separated organelles. Nature *573*, 144–148.
39. Meikar, O., Da Ros, M., Liljenbäck, H., Toppari, J., and Kotaja, N. (2010). Accumulation of piRNAs in the chromatoid bodies purified by a novel isolation protocol. Exp. Cell Res. *316*, 1567–1575.
40. Meikar, O., Vagin, V.V., Chalmel, F., Sõstar, K., Lardenois, A., Hammell, M., Jin, Y., Da Ros, M., Wasik, K.A., Toppari, J., et al. (2014). An atlas of chromatoid body components. RNA *20*, 483–495.
41. Brady, J.P., Farber, P.J., Sekhar, A., Lin, Y.H., Huang, R., Bah, A., Nott, T.J., Chan, H.S., Baldwin, A.J., Forman-Kay, J.D., and Kay, L.E. (2017). Structural and hydrodynamic properties of an intrinsically disordered region of a germ cell-specific protein on phase separation. Proc. Natl. Acad. Sci. USA *114*, E8194–E8203.
42. Voronina, E., Seydoux, G., Sassone-Corsi, P., and Nagamori, I. (2011). RNA Granules in Germ Cells. Cold Spring Harb. Perspect. Biol. *3*, a002774.
43. Holland, J., Crabtree, M.D., and Nott, T.J. (2020). In Vitro Transition Temperature Measurement of Phase-Separating Proteins by Microscopy. Methods Mol. Biol. *2141*, 703–714.
44. Holland, J., Castrejón-Pita, A.A., Tuinier, R., Aarts, D.G.A.L., and Nott, T.J. (2023). Surface tension measurement and calculation of model biomolecular condensates. Soft Matter. https://doi.org/10.1039/D3SM00820G.
45. Oosawa, F., and Kasai, M. (1962). A theory of linear and helical aggregations of macromolecules. J. Mol. Biol. *4*, 10–21.
46. Rosenholm, J.B., Burchfield, T.E., and Hepler, L.G. (1980). Thermodynamics of micelle formation: Standard states, temperature dependence of the critical micelle concentration, and thermal expansion of the solvent. J. Colloid Interface Sci. *78*, 191–194.
47. Baldwin, A.J., Lioe, H., Robinson, C.V., Kay, L.E., and Benesch, J.L.P. (2011). αB-Crystallin Polydispersity Is a Consequence of Unbiased Quaternary Dynamics. J. Mol. Biol. *413*, 297–309.
48. Baldwin, A.J., Knowles, T.P.J., Tartaglia, G.G., Fitzpatrick, A.W., Devlin, G.L., Shammas, S.L., Waudby, C.A., Mossuto, M.F., Meehan, S., Gras, S.L., et al. (2011). Metastability of Native Proteins and the Phenomenon of Amyloid Formation. J. Am. Chem. Soc. *133*, 14160–14163.
49. Hofmeister, F. (1888). Archiv f. experiment. Pathol. u. Pharmakol. *24*, 247–260.
50. Wicky, B.I.M., Shammas, S.L., and Clarke, J. (2017). Affinity of IDPs to their targets is modulated by ion-specific changes in kinetics and residual structure. Proc. Natl. Acad. Sci. *114*, 9882–9887.
51. Po, H.N., and Senozan, N.M. (2001). The Henderson-Hasselbalch Equation: Its History and Limitations. J. Chem. Educ. *78*, 1499–1503.
52. Agudo-Canalejo, J., Schultz, S.W., Chino, H., Migliano, S.M., Saito, C., Koyama-Honda, I., Stenmark, H., Brech, A., May, A.I., Mizushima, N., and Knorr, R.L. (2021). Wetting regulates autophagy of phase-separated compartments and the cytosol. Nat *591*, 142–146.
53. Setru, S.U., Gouveia, B., Alfaro-Aco, R., Shaevitz, J.W., Stone, H.A., and Petry, S. (2021). A hydrodynamic instability drives protein droplet formation on microtubules to nucleate branches. Nat. Phys. *17*, 493–498.
54. Brangwynne, C.P., Eckmann, C.R., Courson, D.S., Rybarska, A., Hoege, C., Gharakhani, J., Jülicher, F., and Hyman, A.A. (2009). Germline P

CellPress
OPEN ACCESS

Granules Are Liquid Droplets That Localize by Controlled Dissolution/Condensation. Science *324*, 1729–1732.

55. Wippich, F., Bodenmiller, B., Trajkovska, M.G., Wanka, S., Aebersold, R., and Pelkmans, L. (2013). Dual Specificity Kinase DYRK3 Couples Stress Granule Condensation/Dissolution to mTORC1 Signaling. Cell *152*, 791–805.

56. Banani, S.F., Rice, A.M., Peeples, W.B., Lin, Y., Jain, S., Parker, R., and Rosen, M.K. (2016). Compositional Control of Phase-Separated Cellular Bodies. Cell *166*, 651–663.

57. Powers, S.K., Holehouse, A.S., Korasick, D.A., Schreiber, K.H., Clark, N.M., Jing, H., Emenecker, R., Han, S., Tycksen, E., Hwang, I., et al. (2019). Nucleo-cytoplasmic Partitioning of ARF Proteins Controls Auxin Responses in Arabidopsis thaliana. Mol. Cell *76*, 177–190.e5.

58. Zheng, W., Dignon, G.L., Jovic, N., Xu, X., Regy, R.M., Fawzi, N.L., Kim, Y.C., Best, R.B., and Mittal, J. (2020). Molecular details of protein condensates probed by microsecond long atomistic simulations. J. Phys. Chem. B *124*, 11671–11679.

59. Jin, X., Zhou, M., Chen, S., Li, D., Cao, X., and Liu, B. (2022). Effects of pH alterations on stress- and aging-induced protein phase separation. Cell. Mol. Life Sci. *79*, 380.

60. Beigi Harchegani, A., Irandoost, A., Mirnamniha, M., Rahmani, H., Tahmasbpour, E., and Shahriary, A. (2019). Possible Mechanisms for The Effects of Calcium Deficiency on Male Infertility. Int. J. Fertil. Steril. *12*, 267–272.

61. Mata-Martínez, E., Sánchez-Cárdenas, C., Chávez, J.C., Guerrero, A., Treviño, C.L., Corkidi, G., Montoya, F., Hernandez-Herrera, P., Buffone, M.G., Balestrini, P.A., and Darszon, A. (2021). Role of calcium oscillations in sperm physiology. Biosystems *209*, 104524.

62. Banerjee, P.R., Milin, A.N., Moosa, M.M., Onuchic, P.L., and Deniz, A.A. (2017). Reentrant Phase Transition Drives Dynamic Substructure Formation in Ribonucleoprotein Droplets. Angew. Chem. Int. Ed. Engl. *56*, 11354–11359.

63. Pullara, P., Alshareedah, I., and Banerjee, P.R. (2022). Temperature-dependent reentrant phase transition of RNA–polycation mixtures. Soft Matter *18*, 1342–1349.

64. Alshareedah, I., Kaur, T., Ngo, J., Seppala, H., Kounatse, L.A.D., Wang, W., Moosa, M.M., and Banerjee, P.R. (2019). Interplay between Short-Range Attraction and Long-Range Repulsion Controls Reentrant Liquid Condensation of Ribonucleoprotein–RNA Complexes. J. Am. Chem. Soc. *141*, 14593–14602.

65. Toyama, Y., Rangadurai, A.K., Forman-Kay, J.D., and Kay, L.E. (2022). Mapping the per-residue surface electrostatic potential of CAPRIN1 along its phase-separation trajectory. Proc. Natl. Acad. Sci. *119*, e2210492119.

66. Whitaker, M. (2006). Calcium at fertilization and in early development. Physiol. Rev. *86*, 25–88.

67. Clapham, D.E. (2007). Calcium Signaling. Cell *131*, 1047–1058.

68. Maynard, C.J., Cappai, R., Volitakis, I., Cherny, R.A., White, A.R., Beyreuther, K., Masters, C.L., Bush, A.I., and Li, Q.X. (2002). Overexpression of Alzheimer's Disease Amyloid-β Opposes the Age-dependent Elevations of Brain Copper and Iron. J. Biol. Chem. *277*, 44670–44676.

69. Bush, A.I., Pettingell, W.H., Multhaup, G., d Paradis, M., Vonsattel, J.P., Gusella, J.F., Beyreuther, K., Masters, C.L., and Tanzi, R.E. (1994). Rapid induction of Alzheimer A beta amyloid formation by zinc. Science *265*, 1464–1467.

70. Schneider, C.A., Rasband, W.S., and Eliceiri, K.W. (2012). NIH Image to ImageJ: 25 years of image analysis. Nat. Methods *9*, 671–675.

71. Waterhouse, A.M., Procter, J.B., Martin, D.M.A., Clamp, M., and Barton, G.J. (2009). Jalview Version 2—a multiple sequence alignment editor and analysis workbench. Bioinformatics *25*, 1189–1191.

72. Stothard, P. (2000). The sequence manipulation suite: JavaScript programs for analyzing and formatting protein and DNA sequences. Biotechniques *28*, 1102–1104.

73. Schulte-Herbrüggen, T., Sørensen, O.W., and Clean, T.R.O.S.Y. (2000). Compensation for Relaxation-Induced Artifacts. J. Magn. Reson. *144*, 123–128.

74. Lescop, E., Schanda, P., and Brutscher, B. (2007). A set of BEST triple-resonance experiments for time-optimized protein resonance assignment. J. Magn. Reson. *187*, 163–169.

75. Delaglio, F., Grzesiek, S., Vuister, G.W., Zhu, G., Pfeifer, J., and Bax, A. (1995). NMRPipe: A multidimensional spectral processing system based on UNIX pipes. J. Biomol. NMR *6*, 277–293.

76. Vranken, W.F., Boucher, W., Stevens, T.J., Fogh, R.H., Pajon, A., Llinas, M., Ulrich, E.L., Markley, J.L., Ionides, J., and Laue, E.D. (2005). The CCPN data model for NMR spectroscopy: Development of a software pipeline. Proteins *59*, 687–696.

77. Tamiola, K., Acar, B., and Mulder, F.A.A. (2010). Sequence-specific random coil chemical shifts of intrinsically disordered proteins. J. Am. Chem. Soc. *132*, 18000–18003.

78. Goldberg, I.G., Allan, C., Burel, J.M., Creager, D., Falconi, A., Hochheiser, H., Johnston, J., Mellen, J., Sorger, P.K., and Swedlow, J.R. (2005). The Open Microscopy Environment (OME) Data Model and XML file: open tools for informatics and quantitative analysis in biological imaging. Genome Biol. *6*. R47-R13.

STAR★METHODS

KEY RESOURCES TABLE

REAGENT or RESOURCE	SOURCE	IDENTIFIER
Bacterial and virus strains		
BL-21 CodonPlus RIL E. coli cells	Agilent	230240
Chemicals, peptides, and recombinant proteins		
Fluo 5F	Invitrogen	F14222
Fluo 5N	Invitrogen	F14204
Deposited data		
Ddx4 NMR chemical shift assignments	This paper	BMRB entry 51711
Experimental models: Cell lines		
HeLa S3	Laboratory of Francis Barr	N/A
Oligonucleotides		
Cy5-ssRNA: Cy5-ACUGACUGACUGACUGACUGACUG	Nott et al.[14]	N/A
Recombinant DNA		
$Ddx4^{N0-4}$, $drDdx4^{N}$ and Bel^{N}, $Ddx4^{CFP1}$, $Ddx4^{YFP0-4}$, $Ddx4^{YFP1}$ tags, $Ddx3x^{N}$Flag, $Ddx3x^{N}$, $Ddx3y^{N}$, $Vasa^{N}$, YFP	This paper	N/A
Software and algorithms		
Python version 2.7 and version 3	Python Software Foundation	https://www.python.org
ImageJ	Schneider et al.[70]	https://imagej.nih.gov/ij/
Mathematica	Wolfram Research, Inc., Mathematica, Version 12.0, Champaign, IL (2019)	N/A

RESOURCE AVAILABILITY

Lead contact

Further information and requests for resources and reagents should be directed to and will be fulfilled by the lead contact, Tim Nott (tim.nott@kcl.ac.uk).

Materials availability

All unique/stable reagents generated in this study are available from the lead contact with a completed materials transfer agreement.

Data and code availability

Ddx4 NMR backbone assignment data have been deposited at in the Biological Magnetic Resonance Bank (BMRB) and are publicly available as of the date of publication. Accession numbers are listed in the key resources table. This paper does not report original code. Any additional information required to reanalyse the data reported in this paper is available from the lead contact upon request.

EXPERIMENTAL MODEL AND STUDY PARTICIPANT DETAILS

Cells and culture conditions

BL-21 codon plus RIL *E. coli* cells were grown in the range of 18°C–37°C in terrific broth or minimal media.

HeLa S3 cells were a kind gift from Prof. Francis Barr of the Department of Biochemistry at the University of Oxford. The cells were cultured in high glucose DMEM (Gibco) containing 10% FBS (Sigma)) at 37°C and 5% CO_2. For HeLa cell experiments involving treatment with salts, cells were grown in DMEM (ThermoFisher; 31966021) with 9% FBS (Sigma; F9665) at 37°C and 5% CO_2.

METHOD DETAILS

Protein expression and purification

DNA sequences for $Ddx4^{N0-N4}$, $drDdx4^{N}$ and Bel^{N} constructs were generated by PCR and subcloned into pET SUMO vectors. Transformed BL-21 codon plus RIL E. coli cells were grown at 37°C to an optical density (A_{600}) of 0.6–1 in terrific broth and induced with 0.5 mM IPTG. Protein expression was left to occur overnight at 22°C with shaking at 180 rpm. To reduce protein degradation during

sonication and affinity purification, cell pellets were resuspended in 20 mM sodium phosphate, 10 mM imidazole, 6 M guanidinium hydrochloride (GuCl), pH 7.4. Sonicate supernatants were loaded onto Ni-NTA agarose resin (Agarose Bead Technologies) and incubated for >1 h at 4°C. After washing with 20 mM sodium phosphate, 10 mM imidazole, pH 7.4, the bound SUMO-tag was removed by the protease ULP-1, and cleaved protein was eluted from the resin supernatant. Protein was further purified by size exclusion chromatography (SEC), using an elution buffer of 20 mM Tris, 300 mM NaCl, 5 mM TCEP, pH 8 at 22°C.

GST tagged protein constructs (Ddx3x^N, Ddx3y^N, Vasa^N, YFP) were generated by subcloning sequences of interest into a modified pETM-30 vector containing the pGEX-2T-TEV site and pProEx multiple cloning site. Transformed BL-21 codon plus RIL E. coli cells were grown at 37°C to an optical density (A_{600}) of 0.6–1 in terrific broth and induced with 0.5 mM IPTG. Protein expression was left to occur for 4 h at 37°C or overnight at 22°C with shaking at 180 rpm. Cell pellets were typically resuspended in buffer containing 20 mM Tris pH 8 at 22°C, 300 mM NaCl, 2 mM DTT and EDTA-free protease inhibitor tablets (Roche). Sonicate supernatants were loaded onto GST-4B resin (Amersham) and incubated for >1 h at 4°C. Resin was typically washed with 20 mM Tris pH 8, 1.5 M NaCl, 2 mM DTT and then exchanged into 20 mM Tris pH 8 at 22°C, 300 mM NaCl, 2 mM DTT, 2 mM EDTA. The GST-tag was removed by TEV protease overnight at 4°C, and cleaved protein was eluted from the resin supernatant. The protein was further purified by SEC, using an elution buffer of 20 mM Tris, 300 mM NaCl, 1 mM TCEP, pH 8 at 22°C.

SEC fractions corresponding to pure protein were pooled, concentrated using centrifugal filters (Amicon), aliquoted into 0.2 mL thin-walled polypropylene PCR tubes (Axygen), flash frozen in liquid nitrogen, and stored at −80°C. Protein purity and molecular weight of pooled, stored protein were confirmed by SDS-PAGE and electrospray ionization mass spectrometry.

Protein concentration determination

Protein concentrations were determined using a NanoDrop Lite spectrophotometer (Thermo Scientific), absorbance at 280 nm (A_{280}) and the Beer-Lambert law. Reported protein concentrations were the average of at least 3 measurement replicates.

To measure how Ddx4^{N1} supernatant concentration (c_1) was altered by the presence of different salts, Ddx4^{N1} protein stock solution (in SEC elution buffer) was first diluted with a buffer (typically 20 mM Tris, 5 mM TCEP, pH 8) containing various concentrations of different salts, which initiated protein phase separation. Samples were then incubated for 10 min at room temperature (22°C) before centrifuging at 20,000 x g for 5 min at room temperature. The supernatant was aspirated and A_{280} measured as above to yield c_1.Protein concentrations were calculated using an extinction coefficient of 23950 M^{-1} cm^{-1} for Ddx4$^{N0\text{-}N4}$.

To measure how c_1 was altered by changes in pH (Figures 5B and 5C), Ddx4^{N1} was first buffer exchanged into two stock solutions. One contained 10 mM citric acid, 150 mM NaCl, pH 2.5 (82 μM Ddx4^{N1}), and the other contained 20 mM Na_2HPO_4, 100 mM NaCl, pH 8.0 (137 μM Ddx4^{N1}). The pH was modulated by mixing the two stocks in various ratios. pH of mixed solutions was confirmed using a pH meter (Beckman). Note that after mixing, the total ionic strength varied from 145 to 155 mM and the total protein concentration varied from 96 to 133 μM.

Transition temperature (T_p) determination

Unless otherwise stated, T_p experiments were performed in a buffer containing 20 mM Tris (pH 8.0 at 22°C), 150 mM NaCl and 5 mM TCEP. This was typically achieved through mixing protein solutions (stored in 20 mM Tris (pH 8.0 at 22°C), 300 mM NaCl, 5 mM TCEP) with 20 mM Tris (pH 8.0 at 22°C), 5 mM TCEP, which reduced both the ionic strength and protein concentration of the stock solution and promoted protein phase separation. For salt studies, 4x salt solutions (in deionized water (diH2O)) were mixed with 2x buffer solutions (e.g., 40 mM $CaCl_2$ mixed with 40 mM Tris, 10 mM TCEP). This gave a final 2x salt solution in 1x buffer that was mixed with the protein solution (e.g., providing final buffer conditions of 20 mM Tris, 150 mM NaCl, 10 mM $CaCl_2$, 5 mM TCEP). To improve the accuracy of the dilutions, all mixing steps outlined above were performed with a 1:1 volume ratio.

T_p measurements were typically carried out as described previously.[43] Briefly, 0.22 mm thick siliconized glass coverslips (Hampton Research), buffers and protein solutions were preheated using an Eppendorf ThermoMixer C. The ionic strength/salt/pH of the protein solution(s) was altered as above before transferring to the coverslip. The imaging chamber was sealed with 0.12 mm imaging spacers (Sigma) and a second siliconized glass coverslip. Samples were transferred to a pre-heated Linkam PE120xy temperature-controlled imaging stage controlled with LinkSys software (Linkham) and imaged using a 10x differential interference contrast (DIC) objective on an Olympus B×43 microscope. Temperature ramps typically consisted of a 2 °C min^{-1} reduction in temperature and were initiated at least 10°C above the transition temperature.

For analysis, twelve images of an isothermal sample were captured at the peak temperature of the ramp and used to calculate a baseline pixel intensity. Upon phase separation, condensate formation resulted in a change in observed pixel intensity. T_p was then defined as the temperature at which the pixel intensity deviated by > 10 standard deviations of the baseline intensity. Two to four individually prepared repeats were typically collected and averaged (mean) for each protein or salt concentration.

Net charge calculation

To calculate the simple net charge of each protein construct, the proportion of each ionizable group was calculated at each pH using the Henderson-Hasselbalch equation and the following pK_a values; C terminus = 3.6, Asp (D) = 4, Glu (E) = 4.5, His (H) = 6.4, N-term = 7.8, Cys (C) = 8.14, Tyr (Y) = 9.6, Lys (K) = 10.4, Arg (R) = 12.5. This was multiplied by the number and sign of each respective amino acid/ionizable group that was present in the construct, with the net charge determined from the sum of these values. Simple net

charges were calculated by assuming that the net charge of a protein at physiological pH is dominated by the number of its positively charged (R (Arg) and K (Lys)) and negatively charged (D (Asp) and E (Glu)) amino acids.

Sequence alignment and phylogenetic inference

As the net charge on the protein chain predicts its sensitivity to multivalent ions *in vitro*, we sought to explore this characteristic within the evolution of Ddx4 and Ddx3 using a maximum likelihood analysis of the two paralogs and their most sequence-similar homologues in fungi. We collected 94 amino acid sequences of DDX3 and DDX4 from NCBI's reference protein database, representing species from all major Animal phyla, as well as 17 Fungal species to help root the tree. Each sequence in this set was the reciprocal best-scoring BLAST hit for either the human DDX3X or DDX4 proteins. Protein sequences were aligned using MAFFT v740. A subset of sites, corresponding to the predicted DEAD box RNA helicase domains, were readily alignable across species (sequence positions 931–1440 in supplementary alignment, Data S1) and were used for further phylogenetic analyses. The best-fit model of sequence evolution was inferred using PROTTEST to be the LG model with gamma-distributed among-site rate variation and empirical state frequencies. The maximum likelihood (ML) phylogeny and branch lengths were inferred from the protein alignment using raxml 2.0.0-beta.14. The resulting maximum likelihood tree revealed that DDX3 and DDX4 were each monophyletic clades, and that all animal groups appear to have one copy of each paralog. Fungi only contain a single version of the protein (Figure S2A). Statistical confidence in each node in the tree was estimated using the approximate likelihood ratio test (aLRT) statistic.

Strikingly, the difference in the number of positively and negatively charged amino acids $((R\ (Arg)+K\ (Lys)) - (D\ (Asp)+E\ (Glu)))$ full length Ddx3 proteins was very similar to fungal Ddx proteins, both clustering around +3. By contrast, full-length Ddx4 proteins typically contain more negatively charged amino acids than positively charged amino acids, clustering around -11 (Figure S2B). The similarity between Ddx3 and fungal Ddx proteins indicated that the ancestor of Ddx4 and Ddx3 may have shared a net charge slightly above neutral, with Ddx4 evolving toward a net negative charge after its divergence from Ddx3 via a gene duplication that occurred early in animal evolution. Despite clear differences in overall net charge, Ddx4 and Ddx3 protein clades appear to have retained relatively similar levels of condensate stabilising amino acids such as F (Phe) and R (Arg). Our phylogenetic analysis suggested that Ddx4 has evolved and conserved a net negative charge across a vast range of animal lineages, and may therefore be functionally important.

Sequence identity and similarity

Ddx4 and Ddx3 homologue sequences were inspected using Jalview.[71] Percent sequence identity between human, zebrafish, and drosophila Ddx4 and Ddx3x/y N-terminal IDR sequences was calculated using the sequence manipulation suite[72] with amino acid sequence similarity groupings set as AVLIM, FYW, GSTCHNQ, P, KR, DE (Table S2). The residual cloning tag common to all sequences (GAMGS) was not included in the sequence identity and similarity analysis.

NMR

Protein expression and purification was carried out as described above, except minimal media (43 mM NaH_2PO_4, 22 mM KH_2PO_4, 8.5 mM NaCl, 409 μM biotin, 3 mM thiamine HCL, 9.4 mM NH_4Cl, 1 mM D-glucose, 100 μM $FeSO_4$, 2mM $MgCl_2$, 135 μM $CaCl_2$) was used in place of terrific broth. For isotopically labeled samples, NH_4Cl and D-glucose were substituted with ammonium chloride (99% ^{15}N, Sigma-Aldrich) and D-glucose (U-$^{13}C_6$, Goss Scientific Instruments Limited), as required.

NMR experiments were performed at 30°C on 750 and 950 MHz spectrometers equipped with Oxford Instruments Company magnets, Bruker Avance III HD consoles and 5 mm TCI CryoProbes. Assignment of $Ddx4^{N1}$ ^1H, ^{15}N, and ^{13}C resonances was achieved by analysis of 2-dimensional (2D) ^1H-^{15}N BEST-TROSY (BT)[73,74] and 3-dimensional (3D) BT-HNCA, BT-HNCACB, BT-HN(CO)CACB, BT-HN(CA)CO, BT-HNCO, (H)CC(CO)NH, HN(CA)NNH and HN(COCA)NNH double and triple-resonance experiments. All experiments except the 3D BT-HNCACB were collected at 750 MHz. All 3D experiments were collected with 25% non-uniform sampling (NUS) in the two indirect dimensions using standard Bruker sampling schedules. 2D NMR data were processed using NMRPipe.[75] 3D NUS data were processed with the hmsIST software[75] and NMRPipe. Spectra were analyzed and assignments recorded using CcpNmr Analysis versions 2.4 and 2.5.[76] ^1H and ^{13}C chemical shifts were referenced using sodium trimethylsilylpropanesulfonate (DSS) and ^{15}N chemical shifts were referenced indirectly. Chemical shifts of $Ddx4^{N1}$ have been deposited in the Biological Magnetic Resonance Bank (BMRB, Entry ID 51711).

Experiments were performed on samples containing $Ddx4^{N1}$, $Ddx4^{N0}$, and $Ddx4^{N4}$ proteins at concentrations ranging from 18 to 45 μM. 2D ^1H-^{15}N BT experiments, used to monitor backbone amide chemical shifts during salt titrations, were typically conducted in a pH 6.5 buffer containing 20 mM PIPES, 138.5 mM NaCl, 5 mM TCEP, 5% D_2O (Sigma-Aldrich) as only a subset of peaks were present at pH 8 (buffer: 20 mM Tris, 150 mM NaCl and 5 mM TCEP). A typical 2D ^1H-^{15}N BT experiment employed acquisition times of 104.4 and 62.3 ms in the ^1H and ^{15}N dimensions, respectively, with a pre-scan delay of 0.2 s, 128 complex increments of 1024 complex points and 32 scans per increment giving a total experiment time of approximately 1 h. For salt titrations, 3 M NaCl or 1 M (or 3 M) $CaCl_2$ were added to a sample volume of 550 μL, which resulted in additional concentrations of 10–450 mM for NaCl and 3.3–600 mM for $CaCl_2$. Successive 2D ^1H-^{15}N BT spectra were acquired prior to and following each addition NaCl and $CaCl_2$. Protein backbone amides for which 2D ^1H-^{15}N BT peaks could not be unambiguously followed across the whole titration range were not carried forward for analysis. Resonance assignments for $Ddx4^{N1}$ were transferred to $Ddx4^{N0}$ and $Ddx4^{N4}$ via manual inspection of 2D ^1H-^{15}N BT spectra of the respective proteins.

CellPress
OPEN ACCESS

Chemical shift perturbations (CSPs) were calculated as the weighted average of ^1H_N and ^{15}N chemical shifts:

$$CSP = \sqrt{\left[\delta_H{}^2 + (\alpha\delta_N)^2\right]} \quad \text{(Equation 6)}$$

where α is 0.15 (CCPN v2 default) and δ_H and δ_N are the chemical shift differences for backbone amide proton and nitrogen, respectively, following the addition of NaCl or $CaCl_2$.

CSPs were fit to Equation 7 to extract binding affinities:

$$S = \Delta S\left(\frac{K_d + [A] + [B] - \sqrt{\left((K_d + [A] + [B])^2 - 4[A][B]\right)}}{2[A]}\right) \quad \text{(Equation 7)}$$

where S is the chemical shift, K_d is the equilibrium dissociation constant, A is the concentration of protein and B is the additional salt concentration.

Residues that specifically responded to calcium were identified as having a CSP at 30 mM $CaCl_2$ that was larger 0.02 ppm (1 line width). Neighbor-corrected random coil chemical shifts were calculated using the Neighbor Corrected IDP (ncIDP) Library.[77] Deviation from random coil (δRC) was calculated according to:

$$\delta RC = \frac{1}{2}\left(|(H_{ref} - H_{0mM})| - |(H_{ref} - H_{30mM})| + \alpha(|(N_{ref} - N_{0mM})| - |(N_{ref} - N_{30mM})|)\right) \quad \text{(Equation 8)}$$

where H_{ref} and N_{ref} are ncIDP chemical shifts for $Ddx4^{N1}$ backbone amide proton and nitrogen and H_{0mM} and H_{30mM} are the experimentally measured proton chemical shifts at 0 and 30 mM additional $CaCl_2$ (respectively) and N_{0mM} and N_{30mM} are the nitrogen chemical shifts at 0 and 10 mM additional $CaCl_2$ (respectively) and α is 0.15.

Affinity determination of free amino acids for calcium

To provide a signal for the binding of Ca^{2+} to free glutamic and aspartic acid, a calcium sensitive dye (Fluo 5N; Fisher Scientific) that fluoresces upon binding calcium was used. The affinity of this interaction was determined by monitoring the fluorescence intensity of 1.4 μM Fluo 5N in the presence of increasing concentrations of $CaCl_2$ using a Horiba FluoroMax-4 spectrofluorometer. Fluorescence data was fit to Equation 9 to extract the K_d

$$F = F_0 + \Delta F\left(\frac{K_d + [A] + [B] - \sqrt{\left((K_d + [A] + [B])^2 - 4[A][B]\right)}}{2[A]}\right) \quad \text{(Equation 9)}$$

where F is the measured fluorescence intensity, F_0 is the fluorescence intensity in the absence of $CaCl_2$, ΔF is the amplitude of the fluorescence intensity change, K_d is the equilibrium dissociation constant, A is the concentration of Fluo 5N and B is the $CaCl_2$ concentration.

A competition assay format was then used to determine the affinity of the amino acids for calcium. The reduction in signal observed upon incubating different concentrations of each amino acid with a fixed concentration of $CaCl_2$ (100 μM) and Fluo 5N (1.4 μM) was measured and fit to Equation 10 to extract the IC_{50}

$$F = F_0 + \frac{\Delta F}{\left(1 + \frac{[C]}{IC_{50}}\right)^{-n}} \quad \text{(Equation 10)}$$

where F is the measured fluorescence intensity, F_0 is the fluorescence intensity in the absence of $CaCl_2$, ΔF is the amplitude of the fluorescence intensity change, IC_{50} is the midpoint of the binding isotherm, $[C]$ is the concentration of amino acid and n is the Hill coefficient.

To determine the binding affinity of the amino acids for Ca^{2+}, the IC_{50} were converted to equilibrium inhibition constants (K_i) using Equation 11.

$$K_i = \frac{IC_{50}}{\left(1 + \frac{[B]}{K_d}\right)} \quad \text{(Equation 11)}$$

where K_i equilibrium inhibition constant, $[B]$ is the concentration $CaCl_2$ and K_d is the equilibrium dissociation constant for Fluo 5N binding to calcium.

To control for the presence of a C-terminal (carbon 1) carboxylic acid in addition to the sidechain carboxylic acid groups of the free amino acids (D (Asp) and E (Glu)), a competition experiment was also performed with G (Gly).

Settings were adjusted such that the maximum fluorescence intensity was <1,000,000 cps. For Fluo 5N-Ca^{2+} binding experiments the settings were; excitation wavelength of 485 nm (2 nm slit width), emission wavelength of 520 nm (5 nm slit width), 0.5 s integration time, 635 V PMT. For competition experiments, the PMT was increased to 675 V.

Electrophoretic light scattering

Electrophoretic light scattering (ELS) measurements were performed using a Zetasizer Nano (Malvern Panalytical) and folded capillary zeta cells (Malvern Panalytical; DTS1070). Dilute, single-phase protein samples (i.e., samples did not contain protein condensates) were used for ELS measurements and contained a final protein concentration of 15 μM (in 20 mM Tris, 150 mM NaCl, 5 mM TCEP, pH 8 at 22°C). Samples were filtered (0.22 μm) prior to measurement. The mean electrophoretic mobility and associated errors were calculated from 2 to 4 independently prepared sample replicates, with each replicate consisting of at least 3 estimates.

The number of Ca^{2+} ion binding sites was estimated by first considering the concentration of the receptor-ligand ($Ddx4^{N1}$-Ca^{2+}) complex, $[RL]$. Under experimental ELS conditions:

$$[RL] = \frac{([R_T]+[L_T]+K_d) - \sqrt{([R_T]+[L_T]+K_d)^2 - 4[R_T][L_T]}}{2} \qquad \text{(Equation 12)}$$

where $[R_T]$ and $[L_T]$ are the total concentrations of the receptor (0.015 mM) and ligand species (3.3 or 10 mM Ca^{2+} and K_d is the dissociation constant (119 ± 37 mM). Under these conditions, 2.6 or 7.7% of $Ddx4^{N1}$ is predicted to be bound to Ca^{2+} ions ($\%bound = (RL/R_T) * 100$). Using a linear fit of the ELS data for $Ddx4^{N0\text{-}N4}$, for which protein charges at pH 8 are well defined, the charge of $Ddx4^{N1}$ in the presence of 3.3 or 10 mM $CaCl_2$ was predicted to be −4.4 and −3.5, respectively. Assuming that binding of one Ca^{2+} ion alters the charge of an acid sidechain from −1 to +1, the number of binding sites (n) required to account for the change in charge (2.0 and 2.9) upon binding Ca^{2+} ions was 37 and 19, respectively, calculated according to $n = (|\Delta charge|/2) * (100/\%bound)$.

Confocal fluorescence microscopy of Ddx4 condensates *in vitro*

To increase contrast during fluorescence imaging, Cy5 (free or RNA-labelled), Alexa Fluor 488 (Alexa 488; Thermo Fisher Scientific) or mCitrine (YFP), were included at final concentrations of 1 μM, 0.6 μM and 2 μM, respectively. Images were captured at room temperature using a Leica TCS-SP5 confocal fluorescence microscope and an HCX PL APO CS 63x (NA 1.40) oil immersion objective. Samples were illuminated with 488 nm (Alexa 488), 514 nm (YFP), or 633 nm (Cy5) lasers, with power and gain settings adjusted to give mean fluorescence intensity values of protein condensates of approximately 50–70% of the maximum 16-bit depth (~32,000–46,000 a.u.). Images were typically captured with settings of 256 × 256 pixels at ~ 98 × 98 × 98 nm (XYZ) resolution, 1400 Hz scan speed and a line average of 2.

Phase separation was initiated by mixing protein solutions with a buffer of lower ionic strength, as described above, and were left for 10 min to promote droplet growth. Phase separated solutions were then transferred to a 0.22 mm thick siliconized glass coverslip (Hampton Research), before sealing with 0.12 mm imaging spacers (Sigma) and a second siliconized glass coverslip. Samples were then left to equilibrate for approximately 50 min prior to imaging.

Real-time droplet imaging

$Ddx4^{N1}$ (in 20 mM Tris, 300 mM NaCl, 5 mM TCEP) was allowed to phase separate by mixing in a 1:1 volume ratio with 20 mM Tris, 5 mM TCEP, 1.2 μM Alexa 488 (final buffer composition of 20 mM Tris, 150 mM NaCl, 5 mM TCEP, 0.6 μM Alexa 488, pH 8 at 22°C), before transferring 2 μL to a siliconized glass coverslip. To prevent evaporation during the experiment, 10 μL of mineral oil was added on top of the beaded Ddx4 solution, forming a seal. To rapidly collect xyz confocal images (approximately 15 s per stack), settings of 128 × 128 pixels at ~300 × 300 × 300 nm (XYZ) resolution, 1400 Hz scan speed and line average of 2 were used.

Solutions containing $CaCl_2$ and EDTA were generated by mixing a 4x stock with $Ddx4^{N1}$. For example, 20 mM Tris, 5 mM TCEP, pH 8, 40 mM $CaCl_2$, 1.2 μM Alexa 488 was mixed with $Ddx4^{N1}$ (in 20 mM Tris, 300 mM NaCl, 5 mM TCEP) giving final conditions of $Ddx4^{N1}$ in 20 mM Tris, 150 mM NaCl, 5 mM TCEP, pH 8, 20 mM $CaCl_2$, 0.6 μM Alexa 488. Using a 10 μL pipette tip to pierce the mineral oil film, 2 μL of this solution was then added to the solution being imaged. This ensured that all components except the additive (e.g., $CaCl_2$) remained at the same concentration in the sample. Upon removing the pipette tip, the mineral oil reformed the seal, preventing any water loss through evaporation. Data shown are from a single experiment.

Video S2 consists of single xy (lower) and xz (upper) slices of DIC (left) and Alexa 488 fluorescence (right) channels. Horizonal lines at the left of xy slices indicate the position of the corresponding xz slice (and vice versa). Movie frame boarders are white prior to the addition of $CaCl_2$, and change to blue and then to magenta as $CaCl_2$ and EDTA are added, respectively.

In vitro partitioning

Sample preparation for $Ddx4^{N0\text{-}N4}$ condensate partitioning of Alexa 488, Cy5 and Cy5-RNA, and YFP is described in section 'Confocal fluorescence microscopy of Ddx4 condensates *in vitro*'.

Image analyses were performed in Mathematica (Wolfram Research, Inc., Mathematica, Version 12.0, Champaign, IL (2019)). 3D image stacks of sessile droplets resting on a solid substrate were collected using a laser scanning confocal fluorescence microscopy as above. In the positive z-direction, optical sectioning moved out of the solid substrate into the aqueous solution containing droplets.

The xy slices containing the aqueous solution were identified by first measuring the change in pixel standard deviation, per slice, in z. The maxima of the change in standard deviation of pixel intensity in z was taken to approximate the position of the solid-aqueous interface. All images in z positions greater than this value plus 3 were carried forwards. A Gaussian blur was then applied, using the "GaussianFilter" function with a kernel of radius 3, to the 3D image stack composed of the aqueous phase images. The blurred images were then binarized using the "Binarize" function with the default method of Otsu's algorithm. This generated a binary mask containing the condensed protein phase, whilst excluding the dilute protein phase. Dense phase intensity values were then extracted using the "ImageMeasurements" function from the original 3D image stack containing only the aqueous phase as the first argument, "MeanIntensity" as the second argument and an eroded version of the condensed phase mask (generated using the "Erosion" function). Equivalently, dilute phase intensity values were extracted using the same procedure, except for the application of an eroded negative of the condensed phase mask to identify only the dilute phase surrounding protein condensates. In both cases, the erosion ensured no edge effects caused by the blur of dilute phase voxels with dense phase voxels.

YFP typically generated little contrast between the dilute and dense phase, making automated droplet identification difficult. Mean pixel intensities where therefore determined from manually drawn regions of interest from at least five fields of view.

Before calculating the fluorescence intensity ratio (partitioning), background fluorescence from protein condensates and the surrounding dilute phase, determined from samples lacking a fluorophore, were subtracted. Two to four individually prepared repeats, each containing ≥ 5 condensates from different regions of the sample, were collected and averaged (mean) for Alexa 488, Cy5, and Cy5-RNA experiments, while the data from YFP experiments represent multiple condensates from a single sample with ≥ 5 ROIs. Partitioning is expressed as $\Delta G_{part} = RT\ln(K_p)$ where $R = 8.31$, $T = 295.15$, and $K_p = \frac{[inside]}{[outside]}$.

Fluorescence recovery after photo bleaching (FRAP)

Samples were prepared as described above. Samples containing $Ddx4^{N1}$ and $Ddx4^{YFP1}$ (170 and 2.5 μM, respectively) were imaged on an Olympus FV1000 Laser Scanning Microscope based on an Olympus IX81 inverted microscope with a motorised stage and equipped with an Olympus PlanApo N 60x (NA 1.4) oil immersion objective. *In vitro* FRAP was performed on individual 5–10 μm diameter $Ddx4^{N}$ droplets (average diameter 7.6 ± 1.2 μm) using a 515 nm argon laser (YFP channel) to bleach fluorophores. Laser power for the bleach period was 100% for 0.1 s and 2% for acquisition. Fluorescence emission was collected between 530 and 650 nm. Images (128 × 128 pixels) were captured using a 2 μs $pixel^{-1}$ scanning speed, 12-bit depth and 2 x line averaging for a total of 100 time points (2 s interval) with 10 data points recorded pre-bleach. Image analysis was performed using Fiji.[52] Image stacks corresponding to each FRAP experiment were aligned with the StackReg ImageJ plugin using the rigid body transformation method.

Individual FRAP curves were fit to

$$R = R_{tot} - A_1 e^{(-r_1 x)} - A_2 e^{(-r_2 x)} \quad \text{(Equation 13)}$$

where R is recovery, R_{tot} is the total recovery, A_1 and A_2 are fast and slow amplitudes, respectively, r_1 and r_2 are fast and slow rates, respectively, and x is time. Fitted curves, normalized to the mean of their respective pre-bleach signals, were averaged to yield the data presented in Figure S5A. Individual FRAP series data were interpreted using Fick's law of diffusion, as described previously.[13]

Contact angle determination

Contact angles were determined using the Contact Angle plugin available via the NIH distribution of ImageJ.[70] Fits were determined via the manual points procedure from 2 baseline points and 5 defining the condensate profile. For each construct and condition, the data represent the mean contact angle of 3–11 condensates.

HeLa cell culture and transfection

HeLa cells were cultured as previously described.[13,14] Briefly, HeLa cells were grown on 22 mm diameter #1.5 glass coverslips (Agar Scientific) in growth media (high glucose DMEM (GibcoTM) containing 10% FBS (Sigma)) at 37°C and 5% CO_2. Protein constructs were expressed in HeLa cells from pcDNA 3.1+ (Invitrogen) plasmids by transient transfection using the TransIT-LT1 Transfection Reagent (Mirus). Transfections used 0.5–1 μg plasmid DNA per coverslip and were carried out according to the manufacturer's instructions. Transfected cells were fixed approximately 24 h after transfection.

For HeLa cell experiments involving treatment with salts, cells were grown in DMEM (ThermoFisher; 31966021) with 9% FBS (Sigma; F9665).

Imaging fixed HeLa cells

HeLa cells expressing fluorescent proteins were grown on 22 mm diameter #1.5 glass coverslips (Agar Scientific), washed twice with phosphate buffered saline (PBS) at 37°C and fixed with 4% paraformaldehyde (PFA) in PBS (Alfa Aesar) at 37°C, for 5 min. Cells were then washed three times with PBS to remove excess PFA. Cells were incubated for 5–10 min in the wash solution at each step. Coverslips were mounted on microscope slides (Fisher Scientific) using Immu-Mount (Thermo Scientific Shandon) mounting medium.

Cells were imaged on an Olympus FV1000 Laser Scanning Microscope based on an Olympus IX81 inverted microscope with a motorised stage and equipped with an Olympus PlanApo N 60x (NA 1.4) oil immersion objective. YFP (mCitrine) was excited with

an argon 515 nm laser and YFP fluorescence was collected between (530–650 nm). DIC images were collected using illumination from the 515 nm laser.

Unless otherwise stated, imaging data were collected as stacks of 86 z-slices (256 × 256 pixels (xy), 0.1 μm spacing (z), 2 μs pixel^{-1} scanning speed, 12-bit depth, 2 x line averaging). Image analysis was performed using Fiji.[70] Figures were prepared using Fiji and Omero Figure.[78]

Live cell imaging

HeLa cells grown in #1.5H glass bottom 35 mm imaging dishes (Ibidi; IB-81158) were transfected with Ddx4^{CFP1} approximately 24 h prior to observation and were live at the time of recording. Fluo 5F (Thermo Fisher Scientific, F14222) was reconstituted in anhydrous DMSO to 1 mM. 2 μL of 1 mM Fluo 5F in DMSO was added to cells (1 μM final Fluo 5F concentration) and left to incubate at 37°C, 5% CO_2 for 15 min. Media was changed for fresh, pre-warmed media prior to the start of imaging.

Live cell imaging experiments were performed on a DeltaVision Elite widefield fluorescence microscope equipped with a temperature and CO_2 controlled chamber, motorized stage, and an Evolve 512 EMCCD camera (Photometrics). Microscope hardware, image acquisition, deconvolution, and registration were controlled with Resolve3D softWoRx-Acquire software. Samples were observed using CFP (excitation filter = 438/24 nm, emission filter = 475/24 nm; Ddx4^{CFP1}) and YFP (excitation filter = 513/17 nm, emission filter = 548/22 nm; Fluo 5F) filter sets and an Olympus UPlanSApo 60x (NA 1.35) oil immersion objective.

Image stacks (480 × 480 pixels, 16-bit depth, 20 z-slices with 1 μm z increment, 5 msec exposure (CFP channel) and 100 msec exposure (YFP channel)) were captured every 20 s. 20 μL of 1 M $CaCl_2$ (10 mM final) was added 140 s after the start of the live cell recording. Image stacks were registered in z and deconvolved (enhanced ratio, medium noise (200 nm) filtering, 10 cycles) prior to analysis.

Video S1 consists of single z-slices corresponding to those nearest the mid-plane of the cell nucleus at each time point. Three cells within the same field of view (Video S1) were analyzed using Fiji. For each cell at each timepoint, the mean pixel intensity above the mean of the whole stack (for that cell) was divided by the maximum pixel intensity (for that cell) (Figure S1A).

In cell determination of condensate stability of Ddx4^{YFP0-4} charge series and Ddx4^{YFP1} C-terminally tagged constructs

HeLa cells were grown, transfected, fixed, and imaged as described above. Imaging data was collected with identical laser power and detector sensitivity so that fluorescence intensities across difference samples were directly comparable. Image analyses to extract intensities for Ddx4^{YFP0-4} and Ddx4^{YFP1}[tag] condensates and the surrounding nucleoplasm (excluding nucleoli) were performed using Fiji. First, maximum intensity z-projections were made from each stack. Regions of the images corresponding to nucleoli and extra-nuclear regions were then manually removed. The remaining fluorescence signals corresponded to Ddx4^{YFP0-4} and Ddx4^{YFP1}[tag] condensates and their surrounding nucleoplasm. Histograms of pixel intensity (128 bins) typically showed a bimodal distribution of fluorescence intensity. The modal bin in the low pixel intensity region was taken as a proxy for nucleoplasmic Ddx4 protein concentration and the modal bin in the high pixel intensity region was taken as a proxy for Ddx4^{YFP0-4} and Ddx4^{YFP1}[tag] protein in concentration inside condensates. Fluorescence intensity values for Ddx4^{YFP0-4} and Ddx4^{YFP1}[tag] condensates and corresponding nucleoplasm were then averaged across multiple stacks for the same construct. Data represent the mean of ≥10 cells per construct. Condensate stability in cells is expressed as $\Delta G_{partitioning} = RT\ \ln(K_p)$ where $K_p = \frac{[A]}{[B]}$, R = 8.31415, and T = 310.15.

Flow cytometry

HeLa cells were grown in 15 cm plates to a confluency of approximately 50% and transfected with 10 μg of DNA. Expression was allowed to proceed over a 24-h incubation period at 37°C and 5% CO_2, after which cells had typically reached a confluency of 70–80%. Cells were then dissociated using trypsin, pelleted by centrifugation (3000 g for 5 min), and resuspended in PBS. To ensure differences in transfection efficiencies and passage number were not conflated with differences between salt conditions, resuspended cells from the same plate were split into different tubes. Cells were then pelleted and resuspended in 20 mM HEPES, 137 mM NaCl, 2.7 mM KCl, pH 7.4 at 37°C, with or without additional salt (e.g., 20 mM HEPES, 137 mM NaCl, 2.7 mM KCl, 10 mM $CaCl_2$). HEPES was chosen at this stage to prevent phosphate precipitation by calcium. Cells were pelleted and resuspended again before incubating at 37°C and 5% CO_2 for 10 min. Fixation was achieved by resuspending pelleted cells in PBS with 4% PFA and incubating at room temperature for 10 min, before washing cells with PBS. To concentrate cells for flow cytometry, cells were pelleted and resuspended in 100–200 μL of PBS. Cell data was collected by passing cells through an Amnis Imagestream flow cytometer. Typically, data from 100,000 cells were collected from each experimental replicate.

To identify cells with Ddx4^{YFP1} condensates, two parameters were used. Firstly, as formation of Ddx4^{YFP1} condensates occurs in a protein concentration dependent manner, we identified cells with high fluorescence intensity levels, which indicated high expression of Ddx4^{YFP1}. Secondly, the difference in protein concentration between Ddx4^{YFP1} condensates and the surrounding nucleoplasm results in Ddx4^{YFP1} condensates having significantly higher fluorescence intensity than the surrounding nucleoplasm. We reasoned that cells with high fluorescence contrast would therefore be more likely to have condensates, whereas cells with high concentrations of Ddx4^{YFP1} but no condensates would have a more uniform fluorescence intensity and lower contrast. By plotting fluorescence intensity against fluorescence contrast, a population of cells with high intensity and high contrast could clearly be distinguished (Figure S1B). This population of cells was not present in cells expressing only YFP or cells expressing Ddx4^{YFP1}RtoK, FtoA (Ddx4^{YFP1} with 14 R to K and 11 F to A mutations and does not form condensates). To count cells containing Ddx4^{YFP1} condensates, a boundary was

drawn (gray box Figure S1B). This reduced the number of cells from the initial 100,000 to approximately 1000. Cells within this boundary were then manually inspected to remove false positives and ensure that condensate-like features could be identified. At least three experiments were performed for each salt condition.

Generalised condensate stability model

Experimental measurement

We start by showing that in the limit of extensive self-association of protein, we can associate a generalised association constant (K_a) to the assembly that is characterized by the saturating concentration of monomers in the dilute phase (c_{sat}). The association constant can be linked to a free energy, which we can model using a modified stickers and spacers model to account for the various types of electrostatic interactions we encounter.

A simple aggregation scheme as first described by Oosawa[45] can be introduced as follows:

$$c_1 + c_1 \rightleftarrows c_2 \quad \text{(Equation 14)}$$

$$c_2 + c_1 \rightleftarrows c_3 \quad \text{(Equation 15)}$$

$$c_i + c_1 \rightleftarrows c_{i+1} \quad \text{(Equation 16)}$$

where free monomers, c_1, are freely coming on and off aggregates of size i. Assuming we can approximate a similar associated equilibrium constant K_a for each step, the concentration of each oligomer in the steady state will be $c_i = K_a^{i-1} c_1^n$. The total concentration of monomers summed over all oligomeric states will be

$$c_{tot} = \sum_{i=1}^{\infty} i c_i = c_1 \sum_{i=1}^{\infty} i (c_1 K_a)^{i-1} = \frac{c_1}{(1 - c_1 K_a)^2}, \quad \text{(Equation 17)}$$

which we can rearrange to obtain $K_a = \frac{1}{c_1} - \frac{1}{\sqrt{c_1 c_{tot}}}$. Thus, in the steady state,

$$c_1 = \frac{1}{K_a} - \frac{1 - \sqrt{1+4K_a c_{tot}}}{2 c_{tot} K_a^2}. \quad \text{(Equation 18)}$$

In the limit where $c_{tot} > c_1$ then the equilibrium reduces to a simpler expression

$$c_1 + O \rightleftarrows O \quad \text{(Equation 19)}$$

where monomers can be considered to be in equilibrium with larger aggregates whose concentration does not appreciably vary upon monomer addition, and $K_a^{sat} = \frac{1}{c_1^{sat}}$. In this limit we can link the saturating free monomer concentration with K_a^{sat} and, in turn, associate this with a free energy that measures the stability of the oligomers, $\Delta G_{sat} = -RT \ln c_{sat} = RT \ln K_a^{sat}$. Defined in this way, a positive ΔG_{sat} means a low c_{sat} and hence stable droplets.

This approach does not require that all association steps have identical association equilibrium constants, just that the system as a whole can be well explained by an aggregation scheme that is well described by a single averaged association equilibrium constant.

We note that in a Flory-Huggins 2-state phase separation model, we do not expect c_{sat} to vary with total protein concentration. In such a situation, physical intuition might suggest the use of an expression such as $\Delta G_{FH} = -RT \ln(c_{sat})$[17]. The two results converge when $c_{tot} \gg c_1$ and $\Delta G_{FH} = \Delta G_{sat}$. Pragmatically, in this work, either ΔG_{FH} or ΔG_{sat} can be used for modeling and fitting, and the core conclusions are unchanged. The only difference of note is a modest variation in the fitting parameters obtained from the global modeling. However, empirically, we observe variation in c_{sat} with c_{tot}. We would expect this in cases where for example salt concentrations vary in the two phases, meaning that the complete phase diagram is effectively multi-dimensional, and there are limitations associated with assuming that the projection of the phase diagram behaves exactly as a simple Flory-Huggins 2-state case.

In the interests of simplicity for this work, we prefer to use the self-association model ΔG_{sat} because of its clear physical intuition that maps directly onto a measure of thermodynamic stability.

Stability model

To understand and model the determinants of stability, we can express ΔG_{sat} as a linear free energy relationship,

$$\Delta G_{sat} = \sum_i p_i \Delta G_i \quad \text{(Equation 20)}$$

where following the principle of stickers and spacers, p_i is the number of pairs of type i, and ΔG_i is the corresponding contribution to ΔG_{sat}. We are in principle free to introduce any reasonable interaction we wish for ΔG_i between any pairs of residues or residue types. Here, we are going to introduce four types of interaction, stabilising interactions that come from patterned FG/RG repeats $\Delta G_{FG/RG}$, known in the case of Ddx proteins to stabilise condensates,[13,41] repulsions between either negatively or positively charged residues

ΔG_{pp} and ΔG_{nn}, and a generalised electrostatic attraction between negatively and positively charged residues ΔG_{pn}.

$$\Delta G_{sat} = p_{FG/RG}\Delta G_{FG/RG} + p_{pp}\Delta G_{pp} + p_{nn}\Delta G_{nn} + p_{pn}\Delta G_{pn} \quad \text{(Equation 21)}$$

The number of pairs will be $p_{FG/RG} = x_{FG}x_{RG}$, $p_{pp} = \frac{1}{2}x_p(x_p - 1)$, $p_{nn} = \frac{1}{2}x_n(x_n - 1)$ and $p_{pn} = x_p x_n$, yielding

$$\Delta G_{sat} = x_{FG}x_{RG}\Delta G_{FG/RG} + \frac{1}{2}x_p(x_p - 1)\Delta G_{pp} + \frac{1}{2}x_n(x_n - 1)\Delta G_{nn} + x_p x_n \Delta G_{pn} \quad \text{(Equation 22)}$$

Three further extensions are required. First, we need to account for the variation in positive/negative residues with pH, secondly we further adjust these counts by allowing cations to bind to negatively charged groups and finally we rescale the individual free energies according to an extended Debye-Hückel treatment to account for salt screening.

We can account for the effects of pH by rescaling the counts of the positive and negative residues.

$$x_n' = \sum^{\Gamma} x_n^{\Gamma}(0)\frac{1}{1+10^{\left(pK_a^{\Gamma} - pH\right)}} \quad \text{(Equation 23)}$$

$$x_p' = \sum^{\Gamma} x_p^{\Gamma}(0)\frac{1}{1+10^{-\left(pK_a^{\Gamma} - pH\right)}} \quad \text{(Equation 24)}$$

where $x_{n/p}(0)$ are the straight count of negatively/positively charged residues of a given amino acid type Γ with residues contributing to x_n' (pK_a: C-term 3.6, Cys 8.14, Glu 4.5, Asp 4.0, His 6.4) and x_p' (pK_a: N-term 7.8, Arg 12.5, Lys 10.4, Tyr 9.6). For the acidic residues in x_n' when $pH \gg pK_a$, $x_n \rightarrow 0$, and when $pH \ll pK_a$, $x_n \rightarrow \sum^{\Gamma} x_n^{\Gamma}(0)$. A similar result applies for the positively charged residues.

We will further allow positively charged ions I of charge z_c to bind the negatively charged residues R^- according to the following dissociation equilibrium characterized by K_d^{Γ}:

$$R^- I^{z_c} \rightleftarrows R^- + I^{z_c} \quad \text{(Equation 25)}$$

The total concentration of negatively charged residues will be $c_{tot}x_n$. The proportion of negatively charged residues that will be unbound, where $[I]$ is the total ion concentration is given by

$$U(x_n') = \frac{1}{2x_n c_{tot}}\left(c_{tot}x_n' + [I] + K_d - \sqrt{\left([I] - c_{tot}x_n' + K_d\right)^2 + 4c_{tot}x_n' K_d}\right) \quad \text{(Equation 26)}$$

from which we can calculate a modified count of negatively charged residues, and also account for the effects of charge inversion by further subtracting the charge of the complex from the total count. This is only going to contribute when $z_c > 1$:

$$x_n = x_n'(1 - U(x_n')) + x_n' U(x_n')(1 - z_c) \quad \text{(Equation 27)}$$

$$x_p = x_p' \quad \text{(Equation 28)}$$

In the case of this work, we do not consider anion binding, but this can be included by re-writing Equation 28 in the form of Equation 27. Finally, the strength of the electrostatic pairwise interaction free energies can be screened by the addition of salt. To model this, we can consider each pairwise interaction to be an equilibrium between two charged species (defined noting that ΔG_{sat} is positive when condensates are stabilised)

$$A^{z_a}B^{z_b} \rightleftarrows A^{z_a} + B^{z_b} \quad \text{(Equation 29)}$$

The free energy associated with this process is properly specified by an equilibrium constant involving the activities of the ions. These are the product of the concentration of species i, and its activity coefficient γ, such that $a = [c_i]\gamma_i$:

$$\Delta G = -RT \ln \frac{[A][B]\gamma_A \gamma_B}{[AB]}. \quad \text{(Equation 30)}$$

We can separate this into a term containing only the solution concentrations, $\Delta G_0 = -RT \ln \frac{[A][B]}{[AB]}$, and the activity coefficients:

$$\Delta G = \Delta G_0 - RT \ln \gamma_A \gamma_B \quad \text{(Equation 31)}$$

Following the Debye-Hückel extended law to account for how electrostatic interactions between two charges in solution are effectively reduced by the presence of external ions, forming an 'ionic atmosphere', we can note that

$$RT \ln \gamma_A \gamma_B = -\frac{z_a z_b A I'}{1+B r_0 I'} \qquad \text{(Equation 32)}$$

where $A = \frac{1}{N_A}\frac{F^2}{8\pi\epsilon\epsilon_0}B$, $B = \left(\frac{F^2}{\epsilon\epsilon_0 RT}\right)^{1/2}$, $I' = (1000.I)^{1/2}$ and the ionic strength $I = \sum_i 1/2 c_i z_i^2,$ where the sum is over all ions present in solution, each at concentration c_i and stoichiometric charge z_i, F is Faraday's constant, R is the gas constant, T is the thermodynamic temperature, N_A is Avogadro's number, ϵ_0 is the permittivity of free space, ϵ is the relative permittivity and r_0 is a factor to rescale the characteristic the size of the 'ionic atmosphere'. The factor of 1000 converts units so that the individual ion concentrations c_i are specified in the chemically sensible unit of mol dm^{-3}. To provide physical insight into this result, we note that the Debye length r_d at a given ionic strength is set $r_d = (BI')^{-1}$ and so equivalently:

$$RT \ln \gamma_A \gamma_B = -\frac{1}{N_A}\frac{z_a z_b F^2}{4\pi\epsilon\epsilon_0}\frac{1}{2(r_d+r_0)} \qquad \text{(Equation 33)}$$

Which reveals the Debye-Hückel contribution to the electrostatic interaction free energy to be that of one mole of pairs of charges sitting at a distance 2 $(r_d + r_0)$ from each other. This allows us to formally expand any pairwise free energy into an expression that formally includes electrostatic screening:

$$\Delta G = \Delta G_0 + s_r \qquad \text{(Equation 34)}$$

where we define the screening constant

$$s_r = \frac{z_a z_b F^2}{N_A 8\pi\epsilon\epsilon_0}\left(\frac{1}{r_d+r_0}\right) \qquad \text{(Equation 35)}$$

which is a function of the product $z_a z_b$ of an interaction under consideration, the relative permittivity of the medium ϵ, the ionic strength I, and the characteristic distance that adjusts the Debye length, r_0. In the case of a repulsion between two similarly charged ions, the contribution to ΔG from the screening is positive, indicating a reduction in repulsion because of the salt. By contrast, in the case where the two interactions are of opposite sign, we expect the interaction to be stabilising, but the effect of the screening is to reduce the strength of the attraction. Taken together, our model states:

$$\Delta G_{sat} = x_{FG} x_{RG}\left(\Delta G^0_{FG/RG} - s_{FG/RG}\right) + x_p(x_p - 1)\left(\Delta G^0_{pp} + s_{pp}\right) + x_n\left((x_n - 1)\left(\Delta G^0_{nn} + s_{nn}\right) + x_p\left(\Delta G^0_{pn} - s_{pn}\right)\right) \qquad \text{(Equation 36)}$$

Where the counts of negative and positive residues are scaled by salt binding and pH as described by Equations 23, 24, 26 and 27, 28. The model as expressed in Equation 36 is parametrised by 4 ΔG^0 values, an r_0 value per ΔG^0, a global ϵ value and 1 K_d per salt. It is important to note that numerical optimisation of this model is not straight-forward and care needs to be taken to choose good initial parameters, combined with an optimisation strategy that knows how to avoid local minima. Our model has been implemented in python3 using an algorithm that methodically increases the complexity of the model, using a basin-hopping algorithm. Before discussing the global optimisation, we will derive some useful results from Equation 36 that allow physical insight from qualitative observations.

Condensate stability versus charge

We can simply determine ratios of the electrostatic constants by looking at the charge on the chain where we see peak stability. Temporarily neglecting the effects of salt (both binding and screening), we can calculate the charge on the chain (Q), and look at ΔG_{sat} versus Q. By using adjusted counts of x_p and x_n, where pH has already been accounted for, $Q = x_p - x_n$, and noting that in the pH range 3.2–7.6 only x_n is varying, then

$$\Delta G_{sat} = x_{FG} x_{RG} \Delta G_{FG/RG} + \frac{1}{2} x_p(x_p - 1)\Delta G_{pp} + (x_p - Q)\left((x_p - Q - 1)\frac{1}{2}\Delta G_{nn} + x_p \Delta G_{pn}\right). \qquad \text{(Equation 37)}$$

By expanding and grouping constants together, we can identify a parabolic dependence of stability with net charge.

$$\Delta G_{sat} = \alpha + \beta Q + \gamma Q^2, \qquad \text{(Equation 38)}$$

where we can group terms into three constants:

$$\alpha = x_{FG} x_{RG} \Delta G_{FG/RG} + \frac{1}{2} x_p(x_p - 1)\Delta G_{pp} + x_p^2 \Delta G_{pn} + \frac{1}{2}\left(x_p^2 - x_p\right)\Delta G_{nn} \qquad \text{(Equation 39)}$$

$$\beta = -x_p \Delta G_{pn} + \frac{1}{2}(1 - 2x_p)\Delta G_{nn} \qquad \text{(Equation 40)}$$

$$\gamma = \frac{1}{2}\Delta G_{nn} \qquad \text{(Equation 41)}$$

This has some nice features. Firstly, the optimal stability occurs when $Q^{opt} = -\beta/2\gamma$. This allows us to relate the ratio of the attractions, and the negative repulsions to the optimal charge on the chain and the number of positively charged residues:

$$Q^{opt} = x_p\left(\frac{\Delta G_{pn}}{\Delta G_{nn}} + 1\right) - \frac{1}{2} \quad \text{(Equation 42)}$$

$$x_n^{opt} = x_p - Q^{opt} = \frac{1}{2} - x_p\left(\frac{\Delta G_{pn}}{\Delta G_{nn}}\right) \quad \text{(Equation 43)}$$

Which we can rearrange to estimate the ratio of the attractions to the negative repulsions:

$$\frac{\Delta G_{pn}}{\Delta G_{nn}} = \frac{1+2Q^{opt}}{2x_p} - 1 \quad \text{(Equation 44)}$$

As $Q^{opt} = +13, x_p = 32$, we can estimate this ratio to be -0.57, suggesting one term is attractive and the other repulsive, and that the two are similar in magnitude. In this simplistic treatment, we are neglecting the effects of salts, both binding and screening. These additional effects can be accounted for in a global analysis (Figures 6B and S4B; Table S3), but the above analysis shows that significant insight can be achieved from simply noting the charge on the chain where condensates are maximally stable.

We can rearrange this to find the optimum number of negatively charged residues for a given number of positive residues:

$$x_n^{opt} = x_p - Q^{opt} = \frac{1}{2} - x_p\left(\frac{\Delta G_{pn}}{\Delta G_{nn}}\right) \quad \text{(Equation 45)}$$

With $x_p = 32, x_n^{opt} = 18$. As $x_n^o = 36$, we can see that for optimally stable condensates, we need to half the total charge. This can be achieved when the pH is approximately equal to the average pK_a of the negatively charged residues. This matches exactly our observations (Figure 5C).

Interpreting Equation 44 more broadly, we can see that the charge on the chain for maximally stable condensates will be positive if $\Delta G_{pn} < |\Delta G_{nn}|$, where generalised repulsions dominate attractions, with the optimal charge increasing as $|\Delta G_{nn}|/\Delta G_{pn}$ increases. This follows from the requirement that optimally stable condensates have fewer negatively charged resides, and so the optimal charge on the chain approaches x_p.

Optimal pH

We can perform a similar calculation looking at pH more directly. By comparing the pH where condensates are maximally stable to the pK_a of the titratable residues, we can estimate the ratio of the electrostatic interaction free energies. We first explicitly include the pH dependence of the negatively charged residues (which assumes we are operating in a regime where the number of positively charged residues is pH independent), and calculate

$$\frac{d\Delta G_{sat}}{dpH}. \quad \text{(Equation 46)}$$

Setting this to zero allows us to solve for the optimal pH, where x_n^o is the total number of negatively charged residues before scaling by pH:

$$pH^{opt} = pK_A - \frac{1}{\log[10]}\log_{10}\left(Abs\left[1 - \frac{2\,\Delta G_{nn}\,x_n^o}{\Delta G_{nn} - 2\,\Delta G_{pn}x_p}\right]\right) \quad \text{(Equation 47)}$$

This can be re-arranged to allow the ratio of the electrostatic interactions to be estimated from x_n^o, x_p, the average pK_a and the optimum pH.

$$\frac{\Delta G_{pn}}{\Delta G_{nn}} = \frac{1}{x_p}\left(\frac{1}{2} - \frac{x_n^0}{\pm 10^{pK_a - pH^{opt}} + 1}\right) \quad \text{(Equation 48)}$$

There are strictly two solutions to this, but as we expect ΔG_{nn} and ΔG_{pn} to be opposite in sign, we expect the positive solution to be the more useful expression. Finally, noting that in the case of the Ddx sequences, the pK_a is approximately equal to pH^{opt}, and so

$$\frac{\Delta G_{pn}}{\Delta G_{nn}} = \frac{1 - x_n^o}{2x_p}. \quad \text{(Equation 49)}$$

For the Ddx chains studied here, $x_n^o = 36$, $x_p = 32$, and the ratio is -0.55. This estimate is very similar to the estimate of this ratio obtained from noting that the maximal chain stability occurs at +13. Comparing the pH where condensates are maximally stable to the numbers of positive and negative residues provides a convenient method by which the internal electrostatic interactions can be studied.

CellPress
OPEN ACCESS

Optimal condensate stability

We can further examine the chain composition to determine the numbers of positive and negative residues that leads to maximum condensate stability. Firstly, as R (Arg) are positively charged residues, $x_{RG} = x_p - x'_p$, where x'_p are the positively charged residues that are not in RGs (arg gly dipeptides). As we expect $\Delta G_{FG/RG}$ to be the dominant term, we would expect that decreasing x'_p, by replacing positively charged residues that are not RGs for RGs should lead to more stable condensates, provided the characteristic FG/RG placement patterns are maintained.

We can optimise ΔG_{sat} by taking its derivatives with respect to x_p and x_n, setting these to zero, and solving for the optimal number of positive and negative residues, for which the net chain charge $Q^{opt} = x_p^{opt} - x_n^{opt}$. Several regimes are possible. For simplicity, we note the repulsions ΔG_{nn} and ΔG_{pp} will always be negative, the attractions ΔG_{pn} and $\Delta G_{FG/RG}$ will be positive, and we can define the following ratios:

$$A = \frac{\Delta G_{FG/RG}}{\Delta G_{pn}}, B = \frac{|\Delta G_{pp}|}{\Delta G_{pn}}, C = \frac{|\Delta G_{nn}|}{\Delta G_{pn}}, \quad \text{(Equation 50)}$$

And so:

$$x_p^{opt} = \frac{B(C+1)+2x_{FG}A}{2BC-2} \quad \text{(Equation 51)}$$

$$x_n^{opt} = \frac{C(1+B+2x_{FG}A)}{2BC-2} \quad \text{(Equation 52)}$$

$$Q^{opt} = \frac{B-C+2x_{FG}A(1-C)}{2BC-2} \quad \text{(Equation 53)}$$

$$\frac{\Delta G^{opt}}{\Delta G_{pn}} = \frac{C(1+B+2Ax_{FG})}{8(BC-1)^2}\Big(BC(B+C+1) - C^2 - 2B - 2Ax_{FG}(2+(C-B-2)C)\Big) - Ax_{FG}(x_p - x_{FG}) \quad \text{(Equation 54)}$$

To determine if the critical point is a maximum or a minimum we consider the second derivatives and determine that the discriminant $D = BC - 1$. If this is greater than zero, the critical point is a maximum, and if less than zero, the critical point will correspond to a minimum. There will only be a global maximum in the stability function if $\Delta G_{nn}\Delta G_{pp} > \Delta G_{pn}^2$, indicating that a global optimum can exist in the case where the repulsion terms dominate the local attractions.

In the case of Ddx proteins, the balance of the interactions depends critically on ionic strength. At very low salt, this condition is satisfied. However, under near physiological conditions when $I = 160\ mM$, $\Delta G_{pp} \approx 0$, and so there is global optimum in the stability. Following from Equation 42, optimising ΔG_{sat} with respect to x_n (holding x_p constant) yields:

$$\left(\frac{\Delta G_{sat}}{\Delta G_{pn}}\right)_{x_p}^{opt} = x_{FG}x_{RG}A + \frac{C}{8} + \frac{x_p}{2}\left(1 + B + x_p\left(\frac{1}{C} - B\right)\right). \quad \text{(Equation 55)}$$

In the limit where B is small then the derivative of this with respect to x_p will always be positive, meaning that increasing the number of positively charged residues (provided the number of negatively charged residues is increased proportionately according to Equation 42) will always stabilise the condensates, reflecting FG/RG interactions dominating condensate stability, with generalised electrostatic attractions further enhancing condensate stability. As $|\Delta G_{nn}| > \Delta G_{pn}$, and $\Delta G_{nn}\Delta G_{pp} < \Delta G_{pn}^2$, the optimal charge carried by the chain will always be positive.

Applying the model to Ddx data in a global optimisation

71 individual ΔG_{sat} measurements were taken with six different cations, Na^+, K^+, Ca^{2+}, Mg^{2+}, Y^{3+} and Gu^+ (guanidinium), using 5 different sequences carrying different numbers of charges, $Ddx4^{N0\text{-}N4}$. The model in Equation 36 is associated with 15 free parameters, 4 ΔG^0 values with 4 r_0 distances to characterize screening, 6 K_d values, one for each ion tested, and one global ϵ value.

Empirically we also know that the patterns of FG/RG residues in the sequence are vital for condensate stability, and specifically the location of the FGs tend to be within positively charged blocks. We have previously created new sequences where we conserve the placement of FG/RG residues, but we scramble the positions of the charged residues ($Ddx4^{CS}$),[13] and find that condensate stability is reduced substantially. Effects such as this are not currently within our model. While a detailed quantitative understanding of condensate stability with respect to the patterns of residues is outside the scope of this work, one could imagine an extension where we distinguish $\Delta G_{FG/RG}$ pairs involving FGs in positively charged segments, and pairs not in such segments to quantitatively tease apart the contributions of each residue to the overall stability.

The average error was 1.7 kJ mol^{-1} with only 4 parameters, excluding ion binding and screening. The systematic variation in ΔG_{sat} with salt concentration is not explained by this model, but the main trend that follows chain stability versus pH is reasonably explained. Including screening lowered the average error to 1.62 kJ mol^{-1}, and the trends in the Ca^{2+}, Mg^{2+} and Y^{3+} addition were not explained. Including ion binding lowered the average error to 1.44 kJ mol^{-1}, and the trends with Na^{+} addition were not explained. Including both ion binding and screening reduced the average error to 0.75 kJ mol^{-1}. As the analysis contains 71 datapoints, the improvements in the quality of the fit is statistically justified for the increasing number of parameters, as determined from an F-test.

Ionic complexes in both free and bound states?

We could consider our interactions between charged groups to be an equilibrium between 'tight' and 'loose' complexes:

$$\{A^{z_a}+B^{z_b}\}_{\text{tight}} \rightleftarrows \{A^{z_a}+B^{z_b}\}_{\text{loose}} \quad \text{(Equation 56)}$$

The free energy associated with this process is properly specified by an equilibrium constant involving the activities of the ions.

$$\Delta G = -RT \ln \frac{[A][B]\gamma_A \gamma_{B,loose}}{[A][B]\gamma_A \gamma_{B,tight}}. \quad \text{(Equation 57)}$$

We can separate this into a term containing only the solution concentrations, $\Delta G_0 = -RT \ln\frac{[A][B]}{[A][B]}$, and the activity coefficients:

$$\Delta G = \Delta G_0 - RT \ln \gamma_A \gamma_{B,loose} + RT \ln \gamma_A \gamma_{B,tight} \quad \text{(Equation 58)}$$

Which yields a modified expression including screening:

$$\Delta G = \Delta G_0 + s_s \quad \text{(Equation 59)}$$

where

$$s_s = \frac{z_a z_b F^2}{N_A 8\pi\epsilon\epsilon_0}\left(\frac{1}{(r_d+r_0)} - \frac{1}{(r_d+nr_0)}\right) = \frac{z_a z_b F^2}{N_A 8\pi\epsilon\epsilon_0}\left(\frac{\Delta r_0}{r_d^2+(n+1)r_0+nr_0^2}\right). \quad \text{(Equation 60)}$$

Where the characteristic distance in the tight complex is nr_0, and $\Delta r_0 = r_0^{tight} - r_0^{loose} = (n-1)r_0$. Further, taking the limit where this difference is small compared to the Debye length:

$$s_s \approx \frac{z_a z_b F^2}{N_A 8\pi\epsilon\epsilon_0}\left(\frac{\Delta r_0}{r_d^2}\right) = \frac{z_a z_b 1000 F^4}{N_A 8\pi(\epsilon\epsilon_0)^2 RT} I \Delta r_0 = z_a z_b \zeta I \Delta r_0 \quad \text{(Equation 61)}$$

Where the constant $\zeta = \frac{1000F^4}{N_A 8\pi(\epsilon\epsilon_0)^2 RT}$.

This result is similar to the Debye-Hückel limiting law ($s_d = z_a z_b A I'$) except the scaling here is linear in ionic strength, and smaller in magnitude. Where $\Delta r_0 = 0$, $s_s = 0$. We speculate that this is the cause of the cation-π interaction having a steeper dependence on salt concentration than the other pure electrostatic terms (Figure S4B).

Derivation of ΔG_{FH} from Flory Huggins theory

Using a 2-state Flory Huggins theory, the chemical potential of dilute and condensed phases is given by

$$\Delta\mu_1 = \mu_1 - \mu_1^0 = RT\left(\ln\phi_1 + (1-\mathrm{N}_1)(1-\phi_1) + \chi N_1(1-\phi_1)^2\right) \quad \text{(Equation 62)}$$

Where N_1 is the number of monomer units in the polymer, whose volume fraction in a phase is given by ϕ_1, and χ is the Flory Huggins interaction constant, a measurement of the difference in stability when moving between the two phases.

One of the conditions for the binodal, the point at which two phases can coexist, is $\mu_1^a = \mu_1^b$, where a and b represent the protein poor and protein rich phases respectively. Inserting the above expression and taking the limit where the protein poor region is dilute, $\phi_1^a \ll 1$, we can write

$$\ln\phi_1^a = \chi\phi_1^b N_1\left(\phi_1^b - 2\right) + \ln\phi_1^b + \phi_1^b(\mathrm{N}_1 - 1) \quad \text{(Equation 63)}$$

Finally, we can express this in terms of a concentration, $\phi_1^a = c_{sat}N_1V_0$ where V_0 is the molar segment volume, and so:

$$\Delta G_{FH} = -RT\ln c_{sat} = RT\left(\chi\phi_1^b N_1\left(2-\phi_1^b\right) - \ln\left(\frac{\phi_1^b}{N_1V_0}\right) - \phi_1^b(\mathrm{N}_1-1)\right) \quad \text{(Equation 64)}$$

We can compare the stability of two conditions, α and β, where N_1 is the same using

$$\Delta\Delta G = -RT\ln\frac{c_{sat}^{\alpha}}{c_{sat}^{\beta}} = RT\left(N_1\left(\chi^{\alpha}\phi_1^{b,\alpha}\left(2-\phi_1^{b,\alpha}\right) - \chi^{\beta}\phi_1^{b,\beta}\left(2-\phi_1^{b,\beta}\right)\right) - \ln\left(\frac{\phi_1^{b,\alpha}}{\phi_1^{b,\beta}}\right) - \left(\phi_1^{b,\alpha}-\phi_1^{b,\beta}\right)(\mathrm{N}_1-1)\right) \quad \text{(Equation 65)}$$

Which is a complicated expression. If we impose the limit that the volume fraction in the condense phases are also the same then we obtain:

$$\Delta\Delta G = -RTln\frac{c_{sat}^{\alpha}}{c_{sat}^{\beta}} = 2\left(\chi^{\alpha} - \chi^{\beta}\right)RT\phi_1^b N_1 \quad \text{(Equation 66)}$$

$\Delta\Delta G$ defined in terms of $-RTln\frac{c_{sat}^{\alpha}}{c_{sat}^{\beta}}$ under the assumption that the condensate volume fraction is the same has used to great effect in previous work.[17] We note however that the derivation used in this work to obtain this equation has some specific challenges, and the route provided here via Flory Huggins theory is more suitable justification of this result and Equation 66 more general.

In the previous work,[17] $\Delta\Delta G$ was derived through consideration of chemical potentials, where essentially the difference in stability between two measurements can be written

$$\Delta\Delta G^0 = \Delta G^{\alpha} - \Delta G^{\beta} = \mu_{dilute}^{\alpha} + \mu_{condensed}^{\alpha} - \mu_{diluted}^{\beta} - \mu_{condensed}^{\beta}. \quad \text{(Equation 67)}$$

Taking $\mu_{dilute} = \mu^0 - RT\ln c_{sat}$ we can then write

$$\Delta\Delta G^0 = -RT\ln\frac{c_{sat}^{\alpha}}{c_{sat}^{\beta}} + \Delta\Delta G_{cond} \quad \text{(Equation 68)}$$

where $\Delta\Delta G_{cond} = \mu_{condensed}^{\alpha} - \mu_{condensed}^{\beta}$ and so our experimental measure becomes

$$\Delta\Delta G = -RT\ln\frac{c_{sat}^{\alpha}}{c_{sat}^{\beta}} = \Delta\Delta G^0 - \Delta\Delta G_{cond} \quad \text{(Equation 69)}$$

which is a combination of both the desired difference in stability, $\Delta\Delta G^0$, and the difference in chemical potential of the condensed phases $\Delta\Delta G_{cond}$. The earlier work argued essentially that $\Delta\Delta G_{cond}$ will be zero provided that volume fractions (concentrations) of protein in the condensed phase are equal, and so $\Delta\Delta G = -RT\ln\frac{c_{sat}^{\alpha}}{c_{sat}^{\beta}} \approx \Delta\Delta G^0$. There is a problem with this argument. We can also derive this result directly from Flory Huggins theory and leave it in terms of the condensed state potentials,

$$\Delta\Delta G = -RTln\frac{c_{sat}^{\alpha}}{c_{sat}^{\beta}} = RTN_1\left(\chi^{\alpha} - \chi^{\beta}\right) - \Delta\Delta G_{cond} \quad \text{(Equation 70)}$$

Where $\Delta\Delta G_{cond} = \mu_1^{\alpha}(\phi_1^{b,\alpha}) - \mu_1^{\beta}(\phi_1^{b,\beta})$, and intuitively the stability difference is $\Delta\Delta G^0 = RTN_1(\chi^{\alpha} - \chi^{\beta})$. If the volume fractions and chemical potentials of the condensed phase are both equal, therefore $\chi^{\alpha} = \chi^{\beta}$, $c_{sat}^{\alpha} = c_{sat}^{\beta}$ and $\Delta\Delta G = 0$.

More generally then, while the physical intuition that leads to the use of an equation of the form $\Delta\Delta G - RTln\frac{c_{sat}^{\alpha}}{c_{sat}^{\beta}}$ is well founded, and hence all conclusions obtained from using this expression in subsequent analysis in previous work are entirely unaffected by the specific method used to derive the expression. We would suggest that colleagues consider the derivation we have presented here to provide a theoretical underpinning of the result (Equations 64; 66).

QUANTIFICATION AND STATISTICAL ANALYSIS

Python 2.7, Python 3, and Mathematica were used for statistical analyses. Statistical tests, sample sizes, and measures of precision are indicated in the main text, figure legends, STAR Methods, and supplementary material.

Cell Reports, Volume *42*

Supplemental information

Ion binding with charge inversion combined with screening modulates DEAD box helicase phase transitions

Michael D. Crabtree, Jack Holland, Arvind S. Pillai, Purnima S. Kompella, Leon Babl, Noah N. Turner, James T. Eaton, Georg K.A. Hochberg, Dirk G.A.L. Aarts, Christina Redfield, Andrew J. Baldwin, and Timothy J. Nott

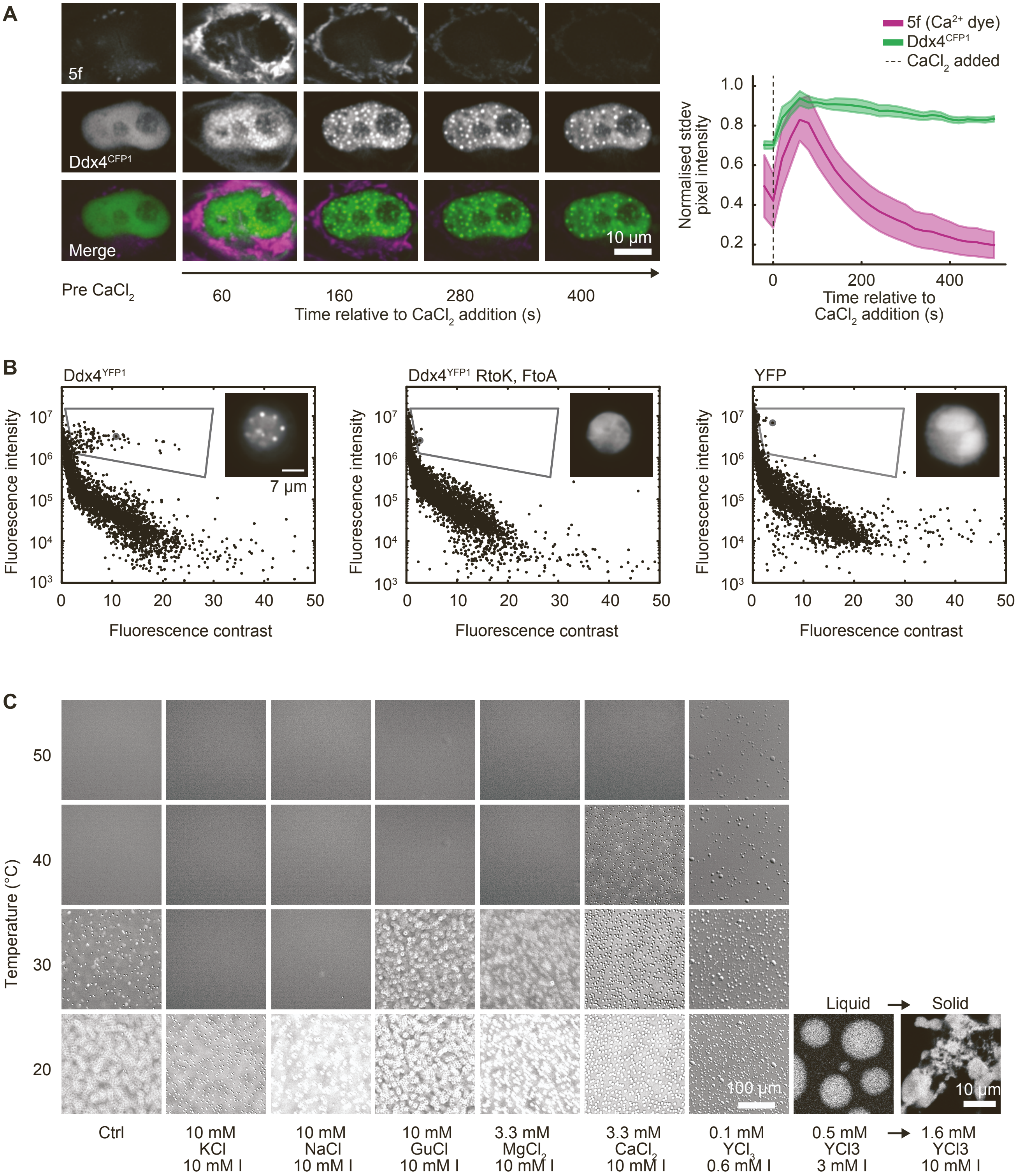

A
5f
Ddx4CFP1
Merge
10 µm
Pre CaCl2
60
160
280
400
Time relative to CaCl2 addition (s)
5f (Ca2+ dye)
Ddx4CFP1
CaCl2 added
Normalised stdev pixel intensity
1.0
0.8
0.6
0.4
0.2
0
200
400
Time relative to CaCl2 addition (s)
B
Ddx4YFP1
Ddx4YFP1 RtoK, FtoA
YFP
Fluorescence intensity
107
106
105
104
103
7 µm
0
10
20
30
40
50
Fluorescence contrast
C
Temperature (°C)
50
40
30
20
Liquid
Solid
100 µm
10 µm
Ctrl
10 mM KCl 10 mM I
10 mM NaCl 10 mM I
10 mM GuCl 10 mM I
3.3 mM MgCl2 10 mM I
3.3 mM CaCl2 10 mM I
0.1 mM YCl3 0.6 mM I
0.5 mM YCl3 3 mM I
1.6 mM YCl3 10 mM I

Supp Fig 1. Cation valency modulates Ddx4^{N1} phase separation propensity.

(A) Snapshots of a live cell imaging experiment in which $CaCl_2$ was added to the growth media surrounding live HeLa cells expressing Ddx4^{CFP1} and continuously monitored (left). Quantitation of the fluorescence emission of a Ca^{2+} ion sensitive dye (Fluo 5F) and Ddx4^{CFP1} showing the coincidence of condensate formation and intracellular influx of Ca^{2+} ions. Scale bar 10 µm. Errors indicate SEM.

(B) Flow cytometry data for HeLa cells expressing Ddx4^{YFP1} indicated a subset of cells with high fluorescence intensity and contrast that were not present in cells expressing either an RtoK + FtoA mutant known to reduce the phase separation propensity of Ddx4[1,2], or YFP alone. Images taken during flow cytometry indicated that this subset was reporting on cells with condensate-like features, allowing the number of cells with condensates to be determined in a high throughput manner. Scale bar 7 µm. A grey box indicates the region where cells with condensates were identified and markers with grey outlines indicate the data point that corresponds to each cell image. Data represents a run of 100,000 cells for each construct, with approximately 1% of Ddx4^{YFP1} cells falling within the region outlined with the grey box.

(C) DIC images indicate the effect of ions on the stability of Ddx4^{N1} condensates. Salt concentrations and ionic strengths (I) are indicated. Scale bar 100 µm. Note the transition from liquid-like to solid-like morphology observed upon increasing YCl_3 concentration from 0.5 to 1.6 mM (fluorescence micrographs where the fluorescence signal comes from free Alexa 488 dye, scale bar 10 µm).

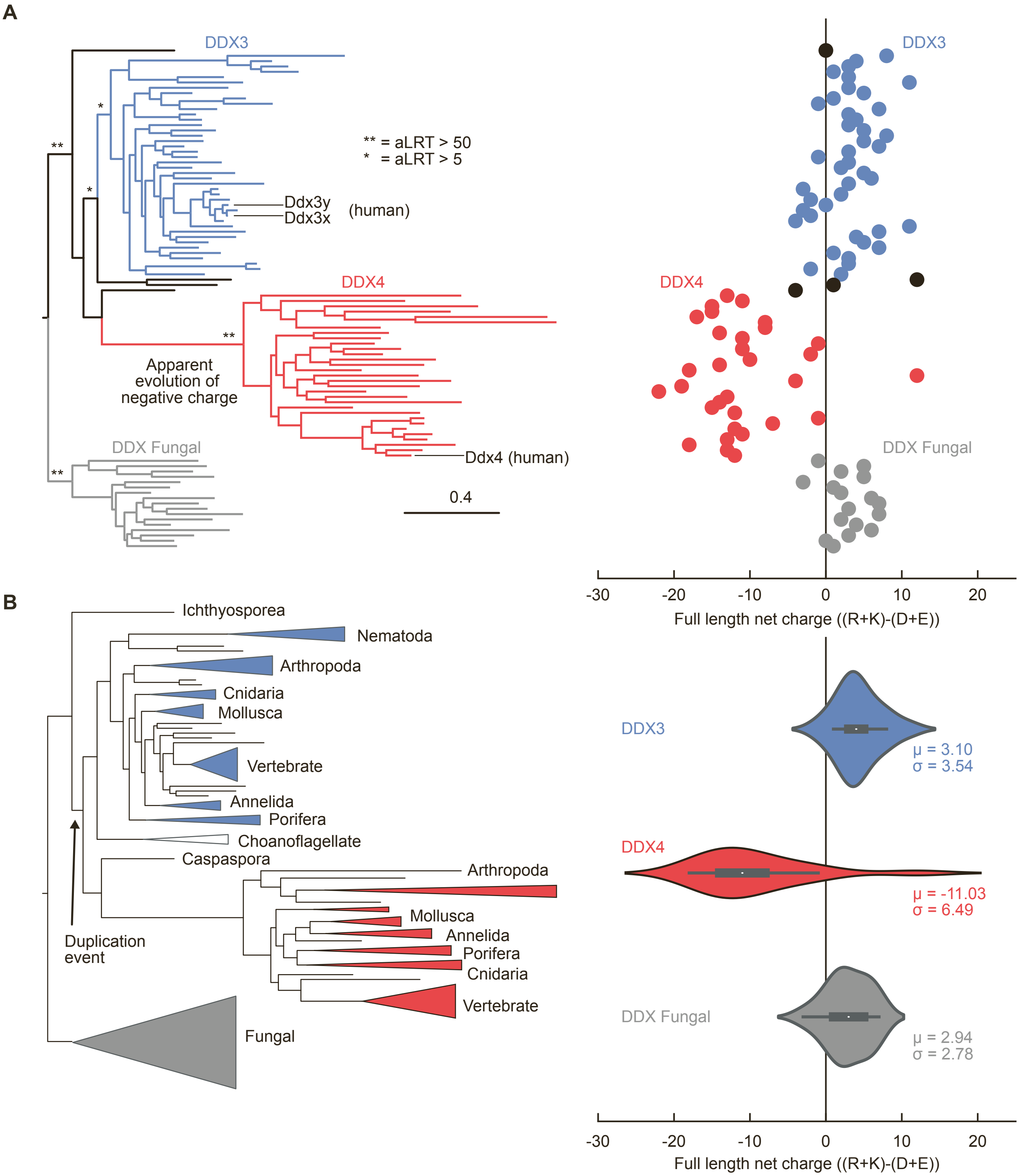

A
DDX3
** = aLRT > 50
* = aLRT > 5
Ddx3y
Ddx3x
(human)
DDX4
Apparent evolution of negative charge
DDX Fungal
Ddx4 (human)
0.4
DDX3
DDX4
DDX Fungal
-30 -20 -10 0 10 20
Full length net charge ((R+K)-(D+E))
B
Ichthyosporea
Nematoda
Arthropoda
Cnidaria
Mollusca
Vertebrate
Annelida
Porifera
Choanoflagellate
Caspaspora
Arthropoda
Mollusca
Annelida
Porifera
Cnidaria
Vertebrate
Duplication event
Fungal
DDX3
μ = 3.10
σ = 3.54
DDX4
μ = -11.03
σ = 6.49
DDX Fungal
μ = 2.94
σ = 2.78
-30 -20 -10 0 10 20
Full length net charge ((R+K)-(D+E))

Supp Fig 2. Evolution of Ddx4 and Ddx3 proteins

(A) Reconstruction of the evolutionary history of DEAD box helicases Ddx4 and Ddx3. Left: Maximum likelihood (ML) tree was constructed based on sequence alignments of the folded domains from 94 animal and fungal species. The animal Ddx4, Ddx3 and fungal clades are shown in red, blue and grey respectively. Eukaryotic clades that are neither animals nor fungi are shown in black. Approximate likelihood-ratio test (aLRT) statistics for selected nodes are indicated. Right: Scatterplot of simple net charge expressed as $(R\ (arg) + K\ (lys)) - (D\ (asp) + E\ (glu))$ for full length Ddx proteins; the values are aligned with branch-tips to which they correspond.

(B) Left: ML phylogeny simplified to show major clades of animals that appear to have one copy of each Ddx paralog. The node that corresponds to the Ddx3/Ddx4 duplication event is indicated. Right: Violin plots of net charge of Ddx4 proteins compared to Ddx3x and fungal Ddx proteins, with mean (μ) and s.d. (σ) indicated.

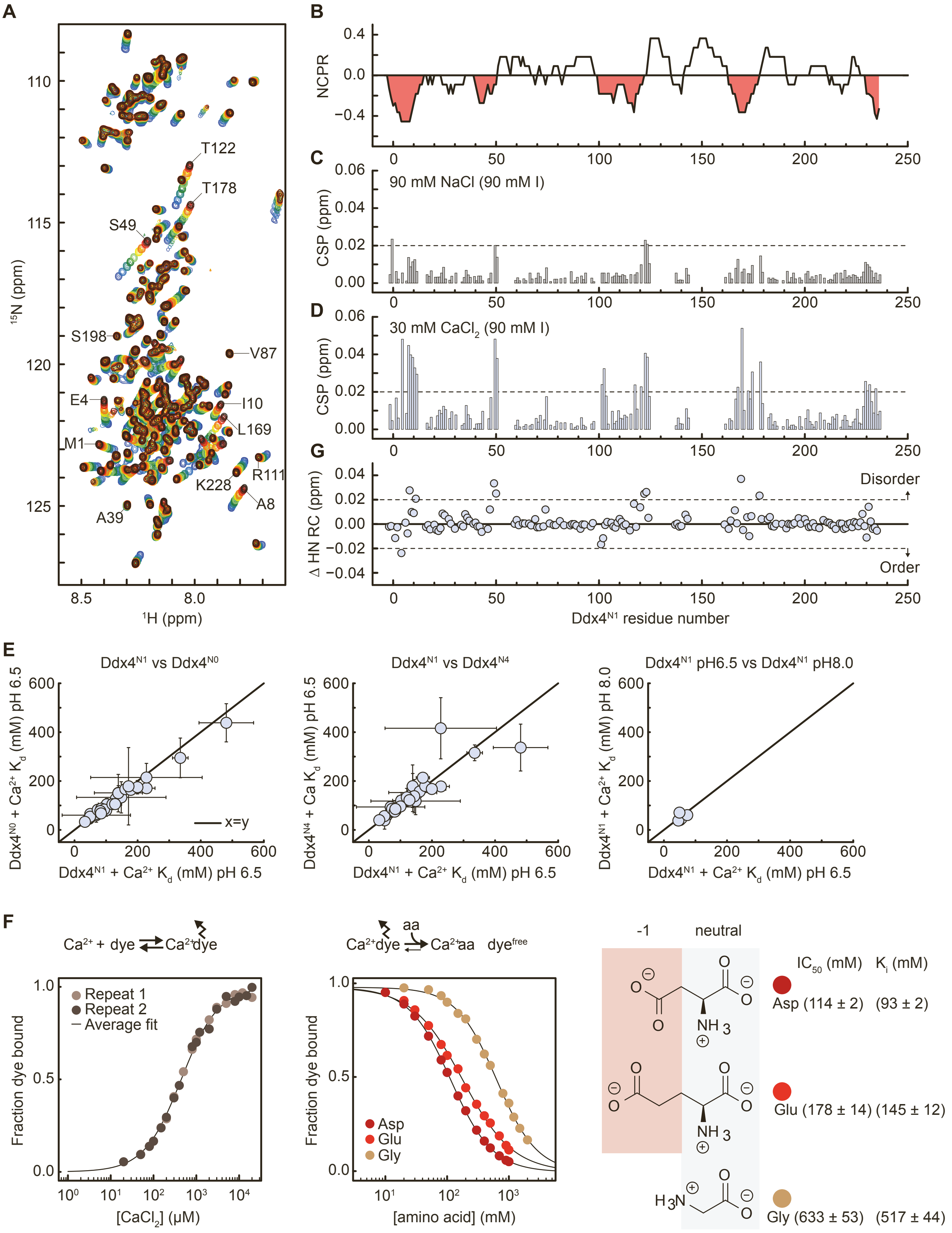

A
B
C
D
G
E
F
T122
T178
S49
S198
V87
E4
I10
L169
M1
R111
K228
A8
A39
¹⁵N (ppm)
¹H (ppm)
NCPR
CSP (ppm)
90 mM NaCl (90 mM I)
30 mM CaCl2 (90 mM I)
Δ HN RC (ppm)
Disorder
Order
Ddx4N1 residue number
Ddx4N1 vs Ddx4N0
Ddx4N1 vs Ddx4N4
Ddx4N1 pH6.5 vs Ddx4N1 pH8.0
Ddx4N0 + Ca2+ Kd (mM) pH 6.5
Ddx4N4 + Ca Kd (mM) pH 6.5
Ddx4N1 + Ca2+ Kd (mM) pH 8.0
Ddx4N1 + Ca2+ Kd (mM) pH 6.5
x=y
Ca2+ + dye ⇄ Ca2+dye
Ca2+dye → Ca2+aa dyefree
aa
Fraction dye bound
Repeat 1
Repeat 2
Average fit
[CaCl2] (μM)
Asp
Glu
Gly
[amino acid] (mM)
-1
neutral
IC50 (mM)
Ki (mM)
Asp (114 ± 2)
(93 ± 2)
Glu (178 ± 14)
(145 ± 12)
Gly (633 ± 53)
(517 ± 44)

Supp Fig 3. The divalent ion Ca^{2+} binds to individual negatively charged sidechains.

(A) ^{1}H-^{15}N BEST-TROSY spectra of $^{15N}Ddx4^{N1}$ at pH 6.5 in the presence of 0 (black), 3.3 (dark red), 10 (red), 30 (orange), 50 (yellow), 100 (light green), 150 mM (green), 250 mM (aqua), 400 mM (light blue), and 600 (dark blue) $CaCl_2$. Residues that show relatively large chemical shift perturbations (CSP) are labelled, as are residues that show minimal CSPs (A39, V87, S198).

(B) Net charge per residue (NCPR, 10 amino acid window, 1 amino acid step) for $Ddx4^{N1}$, calculated using IDDomainSpotter[3].

(C) Per residue CSP for $^{15N}Ddx4^{N1}$ at pH 6.5 at 90 mM additional NaCl compared to 0 mM additional salt. Dashed line indicates a CSP of 0.02 ppm.

(D) Per residue CSP for $^{15N}Ddx4^{N1}$ at pH 6.5 at 30 mM additional $CaCl_2$ compared to 0 mM additional salt. Dashed line indicates a CSP of 0.02 ppm.

(E) Correlations of residue-specific K_d for different samples or conditions compared to the $Ddx4^{N1}$ reference sample. Error bars indicate the estimated standard error for the best-fit value.

(F) Binding isotherm for Ca^{2+} ions and the calcium sensitive dye, Fluo 5N (left). Data from two repeats are shown. Solid line represents the fit values using the mean K_d. A competition-based experiment was used to monitor the binding of amino acids to calcium (right). IC_{50} values were extracted using equation 10 and converted to K_i using equation 11. Carboxylic acid sidechains of D (asp) and E (glu) carry a charge of -1 (pink box). The +1 and -1 charges of the amino and carboxy groups of the amino acid backbone result in a net charge for this region of the molecule of 0 (grey box). Errors represent the error of the fit.

(G) Change in combined ^{1}H and ^{15}N chemical shift relative to the Neighbor Corrected intrinsically disordered protein (IDP) Library (ΔHN RC)[4] at pH 6.5 at 30 mM additional $CaCl_2$ compared to 0 mM additional salt. Dashed line indicates a CSP of 0.02 ppm. Positive and negative values indicate moving towards or away from the reference value for a random coil, respectively.

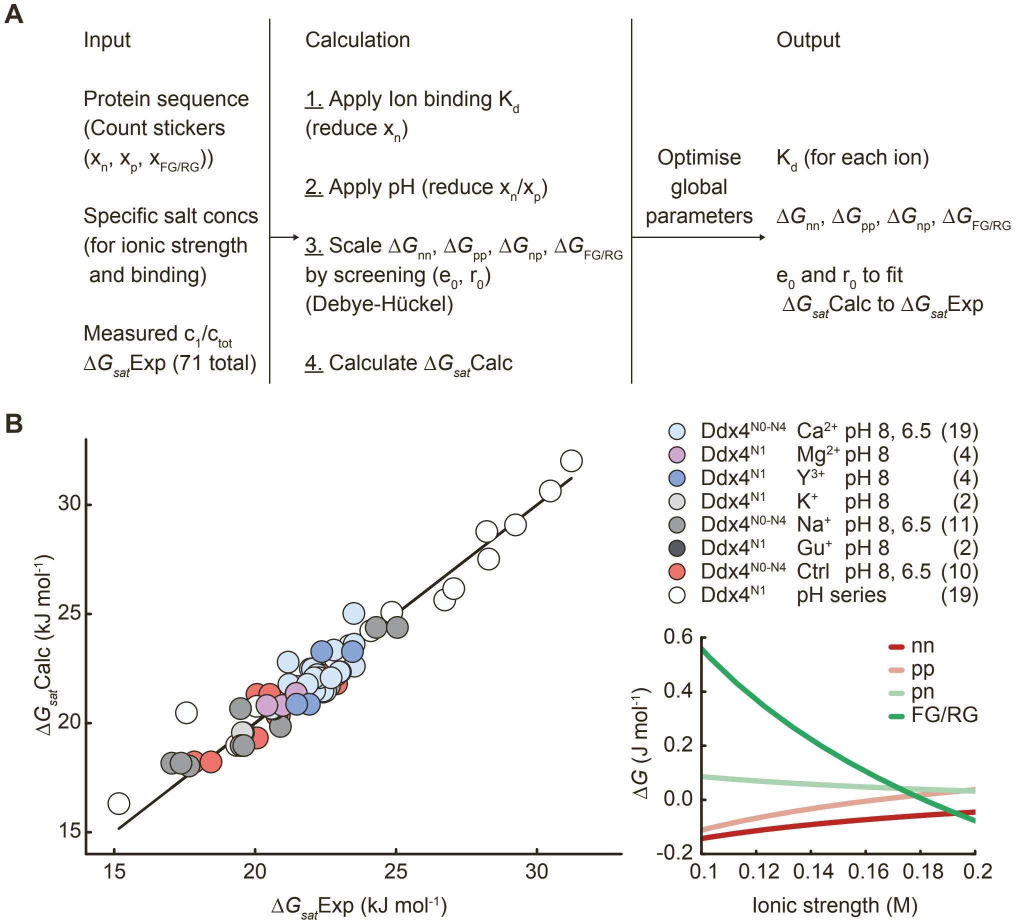
A
Input
Protein sequence
(Count stickers
(x_n, x_p, $x_{FG/RG}$))
Specific salt concs
(for ionic strength
and binding)
Measured c_1/c_{tot}
ΔG_{sat}Exp (71 total)
Calculation
1. Apply Ion binding K_d
(reduce x_n)
2. Apply pH (reduce x_n/x_p)
3. Scale ΔG_{nn}, ΔG_{pp}, ΔG_{np}, $\Delta G_{FG/RG}$
by screening (e_0, r_0)
(Debye-Hückel)
4. Calculate ΔG_{sat}Calc
Optimise
global
parameters
Output
K_d (for each ion)
ΔG_{nn}, ΔG_{pp}, ΔG_{np}, $\Delta G_{FG/RG}$
e_0 and r_0 to fit
ΔG_{sat}Calc to ΔG_{sat}Exp
B
ΔG_{sat}Calc (kJ mol^{-1})
ΔG_{sat}Exp (kJ mol^{-1})
15
20
25
30
Ddx4^{N0-N4} Ca^{2+} pH 8, 6.5 (19)
Ddx4^{N1} Mg^{2+} pH 8 (4)
Ddx4^{N1} Y^{3+} pH 8 (4)
Ddx4^{N1} K^{+} pH 8 (2)
Ddx4^{N0-N4} Na^{+} pH 8, 6.5 (11)
Ddx4^{N1} Gu^{+} pH 8 (2)
Ddx4^{N0-N4} Ctrl pH 8, 6.5 (10)
Ddx4^{N1} pH series (19)
ΔG (J mol^{-1})
0.6
0.4
0.2
0.0
-0.2
0.1
0.12
0.14
0.16
0.18
0.2
Ionic strength (M)
nn
pp
pn
FG/RG

Supp Fig 4. Modeling condensate stability.
(A) Schematic representation of the stickers and spacers model used to explain condensate stability.
(B) Correlation of experimental and calculated ΔG_{sat}, fitted values, and the impact of ionic strength on attractive and repulsive interactions in the model.

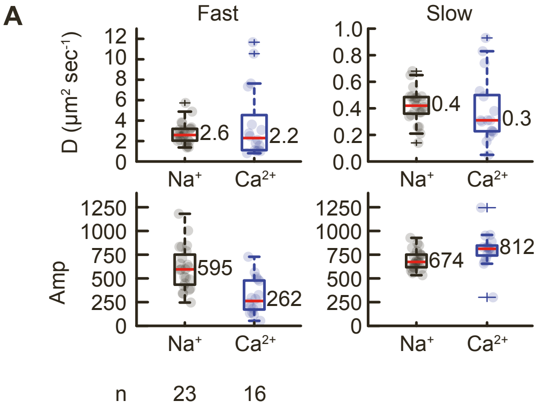

Chi squared values for Na^+ versus Ca^{2+} FRAP datasets:

	Na^+ mono	Na^+ bi	Na^+ ratio: mono/bi	Ca^{2+} mono	Ca^{2+} bi	Ca^{2+} ratio: mono/bi
Mean	124041	14575	8.1	40392	9317	4.4
SD	86039	5262	2.6	27526	4709	1.6

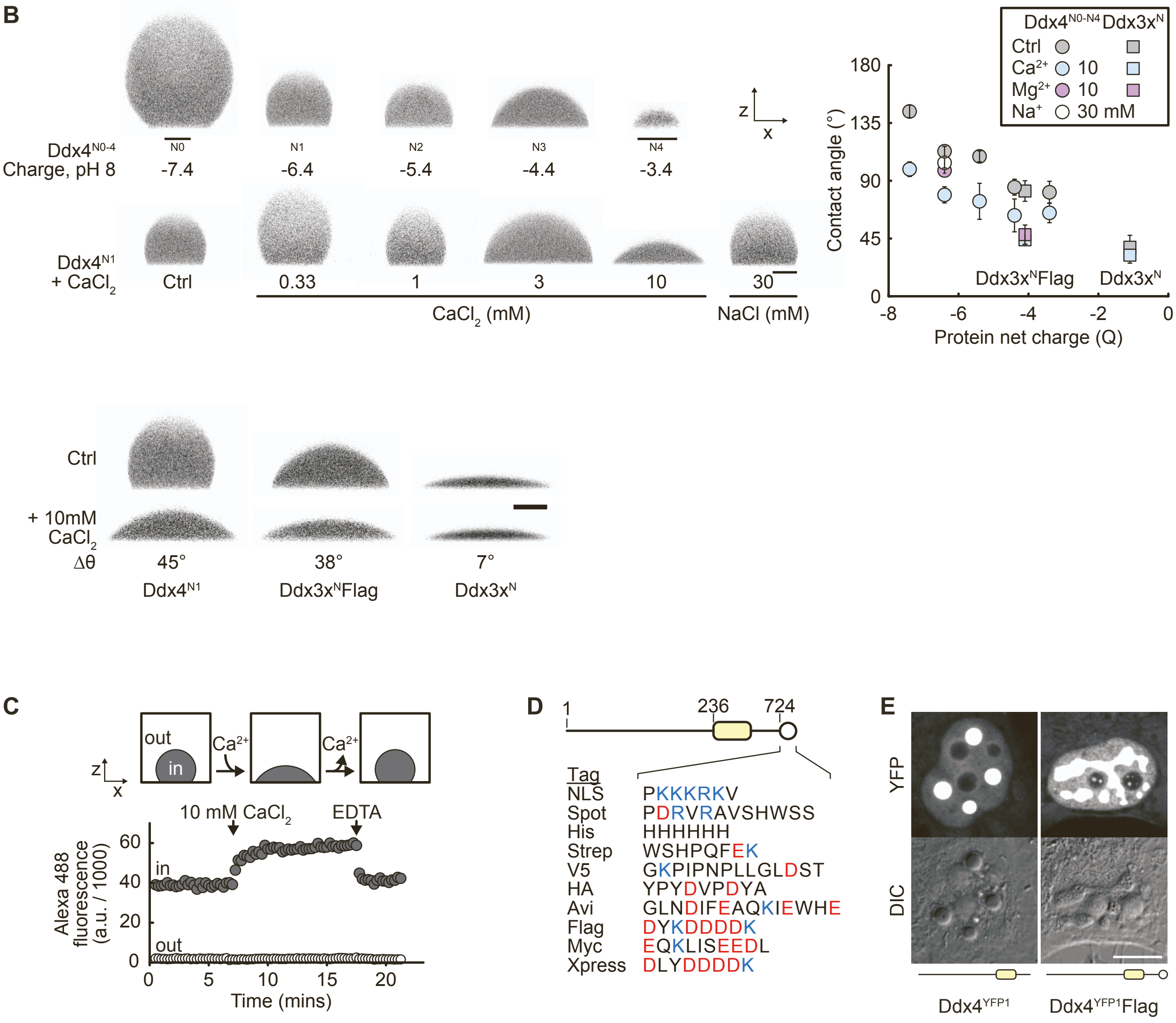

Supp Fig 5. Emergent properties of Ddx4^{N0-N4} biomolecular condensates.
(A) Box plots showing fast and slow diffusion coefficients ($\mu m^2\ sec^{-1}$) and amplitudes from biexponential fits of individual FRAP curves. Red bar indicates median values (indicated for each dataset), box limits indicate interquartile range, whiskers indicate max/min values, crosses indicate outliers. n indicates the number of condensates measured. Comparison of chi squared values from mono and bi exponential fits are tabulated.
(B) Confocal fluorescence microscopy xz images of Ddx4^{N0-N4} charge series, Ddx3x^{N} and Ddx3x^{N}Flag condensates sitting on siliconized glass coverslips indicate the correlation between divalent ion addition, net charge and condensate contact angles. Image contrast, generated using Alexa 488, was adjusted to highlight the profile of the condensates. Scale bars apply to the respective rows of images and indicate 5 µm. Ddx4^{N0} has a unique scale bar in the top row. Plot showing droplet contact angles following addition of 30 mM NaCl, 10 mM $MgCl_2$ or 10 mM $CaCl_2$. Error bars indicate standard deviation.
(C) Summary of the data from Movie S1. Addition of 10 mM $CaCl_2$ to a sample containing Ddx4^{N1} condensates increased the maximum Alexa 488 fluorescence intensity originating inside Ddx4^{N1} condensates (grey) whereas no change in Alexa 488 fluorescence intensity was observed in the dilute phase surrounding Ddx4^{N1} condensates (white). Addition of EDTA (20 mM) returned the condensates to their initial appearance and Alexa 488 fluorescence intensity (partitioning).
(D) Ddx4^{YFP1} was tagged at its C-terminus with a variety of common peptide affinity purification tags. Position and sequences of the tags are indicated. Full protein sequences are given in Table S1.
(E). Morphologies of Ddx4^{YFP1} and Ddx4^{YFP1}Flag condensates inside HeLa cells. The spherical appearance of Ddx4^{YFP1} condensates is consistent with a higher surface tension than the less stable Ddx4^{YFP1}Flag condensates.

Protein name	Sequence
Ddx4^{N1}	GAMGSMGDEDWEAEINPHMSSYVPIFEKDRYSGENGDNFNRTPASSSEMDDGPSRRDHFMKSGFASGRNFGNRDAGECNKR<br>DNTSTMGGFGVGKSFGNRGFSNSRFEDGDSSGFWRESSNDCEDNPTRNRGFSKRGGYRDGNNSEASGPYRRGGRGSFRGCRG<br>GFGLGSPNNDLDPDECMQRTGGLFGSRRPVLSGTGNGDTSQSRSGSGSERGGYKGLNEEVITGSGKNSWKSEAEGGES
Ddx3x^{N}	GAMGSMSHVAVENALGLDQQFAGLDLNSSDNQSGGSTASKGRYIPPHLRNREATKGFYDKDSSGWSSSKDKDAYSSFGSRSDS<br>RGKSSFFSDRGSGSRGRFDDRGRSDYDGIGSRGDRSGFGKFERGGNSRWCDKSDEDDWS
Ddx3x^{N_FLAG}	GAMGSMSHVAVENALGLDQQFAGLDLNSSDNQSGGSTASKGRYIPPHLRNREATKGFYDKDSSGWSSSKDKDAYSSFGSRSDS<br>RGKSSFFSDRGSGSRGRFDDRGRSDYDGIGSRGDRSGFGKFERGGNSRWCDKSDEDDWSDYKDDDDK
Ddx3y^{N}	GAMGSMSHVVVKNDPELDQQLANLDLNSEKQSGGASTASKGRYIPPHLRNREASKGFHDKDSSGWSCSKDKDAYSSFGSRDSR<br>GKPGYFSERGSGSRGRFDDRGRSDYDGIGNRERPGFGRFERSGHSRWCDKSVEDDWS
Vasa^{N}	GAMGSMSDDWDDEPIVDTRGARGGDWSDDEDTAKSFSGEAEGDGVGGSGGEGGGYQGGNRDVFGRIGGGRGGGAGGYRG<br>GNRDGGGFHGGRREGERDFRGGEGGFRGGQGGSRGGQGGSRGGQGGFRGGEGGFRGRLYENEDGDERRGRLDREERGGER<br>RGRLDREERGGERGERGDGGFARRRRNEDDINNNNNIV
Bel^{N}	GAMGSMSNAINQNGTGLEQQVAGLDLNGGSADYSGPITSKTSTNSVTGGVYVPPHLRGGGGNNNAADAESQGQGQGQGQG<br>FDSRSGNPRQETRDPQQSRGGGGEYRRGGGGGGRGFNRQSGDYGYGSGGGGRRGGGGRFEDNYNGGEFDSRRGGDWNRS<br>GGGGGGGRGFGRGPSYRGGGGGSGSNLNEQTAEDGQAQQQQQPRNDRWQEPERPAGFDGSEGGQSAGGNRSYNNRGER<br>GGGGYNSRWKEGGGSNVDYT
drDdx4^{N}	GAMGSMDDWEEDQSPVVSCSSGFGLGSNGSDGGFKSFYTGGAGNDKSNSEGTEGSSWKMTGDSFRGRGGRGGSRGGRGGF<br>SGFKSEIDENGSDGGWNGGESRGRGRGGFRGGFRSGSRDENDENRNDDGWKGGESRGRGRGGFGGSFRGGFRDGGNEDTG<br>RRGFGRENNENGNDEGGEGRGRGRGGFRGGFRDGGGDESGKRGFGRGGFRGRNEEVFSKVTTADKLDQEG
Ddx4^{N0}	GAMGSMGDEDWEAEINPHMSSYVPIFEKDRYSGENGDNFNRTPASSSEMDDGPSRRDHFMKSGFASGRNFGNRDAGECNKR<br>DNTSTMGGFGVGKSFGNRGFSNSRFEDGDSSGFWRESSNDCEDNPTRNRGFSKRGGYRDGNNSEASGPYRRGGRGSFRGCRG<br>GFGLGSPNNDLDPDECMQRTGGLFGSRRPVLSGTGNGDTSQSRSGSGSERGGYKGLNEEVITGSGKNSWKSEAEGGESSD
Ddx4^{N1}	GAMGSMGDEDWEAEINPHMSSYVPIFEKDRYSGENGDNFNRTPASSSEMDDGPSRRDHFMKSGFASGRNFGNRDAGECNKR<br>DNTSTMGGFGVGKSFGNRGFSNSRFEDGDSSGFWRESSNDCEDNPTRNRGFSKRGGYRDGNNSEASGPYRRGGRGSFRGCRG<br>GFGLGSPNNDLDPDECMQRTGGLFGSRRPVLSGTGNGDTSQSRSGSGSERGGYKGLNEEVITGSGKNSWKSEAEGGES
Ddx4^{N2}	GAMGSMGDEDWEAEINPHMSSYVPIFEKDRYSGENGDNFNRTPASSSEMDDGPSRRDHFMKSGFASGRNFGNRDAGECNKR<br>DNTSTMGGFGVGKSFGNRGFSNSRFEDGDSSGFWRESSNDCEDNPTRNRGFSKRGGYRDGNNSEASGPYRRGGRGSFRGCRG<br>GFGLGSPNNDLDPDECMQRTGGLFGSRRPVLSGTGNGDTSQSRSGSGSERGGYKGLNEEVITGSGKNSWKSEAEGG
Ddx4^{N3}	GAMGSMGDEDWEAEINPHMSSYVPIFEKDRYSGENGDNFNRTPASSSEMDDGPSRRDHFMKSGFASGRNFGNRDAGECNKR<br>DNTSTMGGFGVGKSFGNRGFSNSRFEDGDSSGFWRESSNDCEDNPTRNRGFSKRGGYRDGNNSEASGPYRRGGRGSFRGCRG<br>GFGLGSPNNDLDPDECMQRTGGLFGSRRPVLSGTGNGDTSQSRSGSGSERGGYKGLNEEVITGSGKNSWKSEA
Ddx4^{N4}	GAMGSMGDEDWEAEINPHMSSYVPIFEKDRYSGENGDNFNRTPASSSEMDDGPSRRDHFMKSGFASGRNFGNRDAGECNKR<br>DNTSTMGGFGVGKSFGNRGFSNSRFEDGDSSGFWRESSNDCEDNPTRNRGFSKRGGYRDGNNSEASGPYRRGGRGSFRGCRG<br>GFGLGSPNNDLDPDECMQRTGGLFGSRRPVLSGTGNGDTSQSRSGSGSERGGYKGLNEEVITGSGKNSWKS
YFP (mCitrine; recombinantly expressed and purified)	GAMGSMVSKGEELFTGVVPILVELDGDVNGHKFSVSGEGEGDATYGKLTLKFICTTGKLPVPWPTLVTTFGYGLMCFARYPDHM<br>KQHDFFKSAMPEGYVQERTIFFKDDGNYKTRAEVKFEGDTLVNRIELKGIDFKEDGNILGHKLEYNYNSHNVYIMADKQKNGIKV<br>NFKIRHNIEDGSVQLADHYQQNTPIGDGPVLLPDNHYLSYQSKLSKDPNEKRDHMVLLEFVTAAGIT
Ddx4^{YFP0}	MGDEDWEAEINPHMSSYVPIFEKDRYSGENGDNFNRTPASSSEMDDGPSRRDHFMKSGFASGRNFGNRDAGECNKRDNTST<br>MGGFGVGKSFGNRGFSNSRFEDGDSSGFWRESSNDCEDNPTRNRGFSKRGGYRDGNNSEASGPYRRGGRGSFRGCRGGFGLG<br>SPNNDLDPDECMQRTGGLFGSRRPVLSGTGNGDTSQSRSGSGSERGGYKGLNEEVITGSGKNSWKSEAEGGESSDMVSKGEELF<br>TGVVPILVELDGDVNGHKFSVSGEGEGDATYGKLTLKFICTTGKLPVPWPTLVTTFGYGLMCFARYPDHMKQHDFFKSAMPEGY<br>VQERTIFFKDDGNYKTRAEVKFEGDTLVNRIELKGIDFKEDGNILGHKLEYNYNSHNVYIMADKQKNGIKVNFKIRHNIEDGSVQLA<br>DHYQQNTPIGDGPVLLPDNHYLSYQSKLSKDPNEKRDHMVLLEFVTAAGITFSTYIPGFSGSTRGNVFASVDTRKGKSTLNTAGFSS<br>SQAPNPVDDESWD
Ddx4^{YFP1}	MGDEDWEAEINPHMSSYVPIFEKDRYSGENGDNFNRTPASSSEMDDGPSRRDHFMKSGFASGRNFGNRDAGECNKRDNTST<br>MGGFGVGKSFGNRGFSNSRFEDGDSSGFWRESSNDCEDNPTRNRGFSKRGGYRDGNNSEASGPYRRGGRGSFRGCRGGFGLG<br>SPNNDLDPDECMQRTGGLFGSRRPVLSGTGNGDTSQSRSGSGSERGGYKGLNEEVITGSGKNSWKSEAEGGESMVSKGEELFTG<br>VVPILVELDGDVNGHKFSVSGEGEGDATYGKLTLKFICTTGKLPVPWPTLVTTFGYGLMCFARYPDHMKQHDFFKSAMPEGYVQ<br>ERTIFFKDDGNYKTRAEVKFEGDTLVNRIELKGIDFKEDGNILGHKLEYNYNSHNVYIMADKQKNGIKVNFKIRHNIEDGSVQLADH<br>YQQNTPIGDGPVLLPDNHYLSYQSKLSKDPNEKRDHMVLLEFVTAAGITFSTYIPGFSGSTRGNVFASVDTRKGKSTLNTAGFSSSQ<br>APNPVDDESWD
Ddx4^{YFP2}	MGDEDWEAEINPHMSSYVPIFEKDRYSGENGDNFNRTPASSSEMDDGPSRRDHFMKSGFASGRNFGNRDAGECNKRDNTST<br>MGGFGVGKSFGNRGFSNSRFEDGDSSGFWRESSNDCEDNPTRNRGFSKRGGYRDGNNSEASGPYRRGGRGSFRGCRGGFGLG<br>SPNNDLDPDECMQRTGGLFGSRRPVLSGTGNGDTSQSRSGSGSERGGYKGLNEEVITGSGKNSWKSEAEGGMVSKGEELFTGV<br>VPILVELDGDVNGHKFSVSGEGEGDATYGKLTLKFICTTGKLPVPWPTLVTTFGYGLMCFARYPDHMKQHDFFKSAMPEGYVQE<br>RTIFFKDDGNYKTRAEVKFEGDTLVNRIELKGIDFKEDGNILGHKLEYNYNSHNVYIMADKQKNGIKVNFKIRHNIEDGSVQLADHY

	QQNTPIGDGPVLLPDNHYLSYQSKLSKDPNEKRDHMVLLEFVTAAGITFSTYIPGFSGSTRGNVFASVDTRKGKSTLNTAGFSSSQA<br>PNPVDDESWD
Ddx4^{YFP3}	MGDEDWEAEINPHMSSYVPIFEKDRYSGENGDNFNRTPASSSEMDDGPSRRDHFMKSGFASGRNFGNRDAGECNKRDNTST<br>MGGFGVGKSFGNRGFSNSRFEDGDSSGFWRESSNDCEDNPTRNRGFSKRGGYRDGNNSEASGPYRRGGRGSFRGCRGGFGLG<br>SPNNDLDPDECMQRTGGLFGSRRPVLSGTGNGDTSQSRSGSGSERGGYKGLNEEVITGSGKNSWKSEAMVSKGEELFTGVVPIL<br>VELDGDVNGHKFSVSGEGEGDATYGKLTLKFICTTGKLPVPWPTLVTTFGYGLMCFARYPDHMKQHDFFKSAMPEGYVQERTIFF<br>KDDGNYKTRAEVKFEGDTLVNRIELKGIDFKEDGNILGHKLEYNYNSHNVYIMADKQKNGIKVNFKIRHNIEDGSVQLADHYQQN<br>TPIGDGPVLLPDNHYLSYQSKLSKDPNEKRDHMVLLEFVTAAGITFSTYIPGFSGSTRGNVFASVDTRKGKSTLNTAGFSSSQAPNP<br>VDDESWD
Ddx4^{YFP4}	MGDEDWEAEINPHMSSYVPIFEKDRYSGENGDNFNRTPASSSEMDDGPSRRDHFMKSGFASGRNFGNRDAGECNKRDNTST<br>MGGFGVGKSFGNRGFSNSRFEDGDSSGFWRESSNDCEDNPTRNRGFSKRGGYRDGNNSEASGPYRRGGRGSFRGCRGGFGLG<br>SPNNDLDPDECMQRTGGLFGSRRPVLSGTGNGDTSQSRSGSGSERGGYKGLNEEVITGSGKNSWKSMVSKGEELFTGVVPILVE<br>LDGDVNGHKFSVSGEGEGDATYGKLTLKFICTTGKLPVPWPTLVTTFGYGLMCFARYPDHMKQHDFFKSAMPEGYVQERTIFFK<br>DDGNYKTRAEVKFEGDTLVNRIELKGIDFKEDGNILGHKLEYNYNSHNVYIMADKQKNGIKVNFKIRHNIEDGSVQLADHYQQNT<br>PIGDGPVLLPDNHYLSYQSKLSKDPNEKRDHMVLLEFVTAAGITFSTYIPGFSGSTRGNVFASVDTRKGKSTLNTAGFSSSQAPNPV<br>DDESWD
YFP (mCitrine; expressed in HeLa cells)	MVSKGEELFTGVVPILVELDGDVNGHKFSVSGEGEGDATYGKLTLKFICTTGKLPVPWPTLVTTFGYGLMCFARYPDHMKQHDFF<br>KSAMPEGYVQERTIFFKDDGNYKTRAEVKFEGDTLVNRIELKGIDFKEDGNILGHKLEYNYNSHNVYIMADKQKNGIKVNFKIRHNI<br>EDGSVQLADHYQQNTPIGDGPVLLPDNHYLSYQSKLSKDPNEKRDHMVLLEFVTAAGITLERPLESRGPV
Ddx4^{YFP1}RtoK, FtoA	MGDEDWEAEINPHMSSYVPIFEKDRYSGENGDNFNRTPASSSEMDDGPSKRDHFMKSGAASGKNAGNRDAGECNKRDNTST<br>MGGAGVGKSAGNKGASNSKFEDGDSSGFWRESSNDCEDNPTRNKGASKKGGYRDGNNSEASGPYRKGGKGSAKGCKGGAGL<br>GSPNNDLDPDECMQRTGGLAGSKRPVLSGTGNGDTSQSKSGSGSEKGGYKGLNEEVITGSGKNSWKSEAEGGESSDTQGPKVT<br>LQMVSKGEELFTGVVPILVELDGDVNGHKFSVSGEGEGDATYGKLTLKFICTTGKLPVPWPTLVTTFGYGLMCFARYPDHMKQH<br>DFFKSAMPEGYVQERTIFFKDDGNYKTRAEVKFEGDTLVNRIELKGIDFKEDGNILGHKLEYNYNSHNVYIMADKQKNGIKVNFKIR<br>HNIEDGSVQLADHYQQNTPIGDGPVLLPDNHYLSYQSKLSKDPNEKRDHMVLLEFVTAAGITLEASTYIPGASGSTKGNVFASVDT<br>RKGKSTLNTAGFSSSQAPNPVDDESWD
Ddx4^{CFP1}	MGDEDWEAEINPHMSSYVPIFEKDRYSGENGDNFNRTPASSSEMDDGPSRRDHFMKSGFASGRNFGNRDAGECNKRDNTST<br>MGGFGVGKSFGNRGFSNSRFEDGDSSGFWRESSNDCEDNPTRNRGFSKRGGYRDGNNSEASGPYRRGGRGSFRGCRGGFGLG<br>SPNNDLDPDECMQRTGGLFGSRRPVLSGTGNGDTSQSRSGSGSERGGYKGLNEEVITGSGKNSWKSEAEGGESMVSKGEELFTG<br>VVPILVELDGDVNGHKFSVSGEGEGDATYGKLTLKFICTTGKLPVPWPTLVTTLTWGVQCFARYPDHMKQHDFFKSAMPEGYVQ<br>ERTIFFKDDGNYKTRAEVKFEGDTLVNRIELKGIDFKEDGNILGHKLEYNAISDNVYITADKQKNGIKANFKIRHNIEDGSVQLADHY<br>QQNTPIGDGPVLLPDNHYLSTQSKLSKDPNEKRDHMVLLEFVTAAGITFSTYIPGFSGSTRGNVFASVDTRKGKSTLNTAGFSSSQA<br>PNPVDDESWD
Ddx4^{YFP1}His	MGDEDWEAEINPHMSSYVPIFEKDRYSGENGDNFNRTPASSSEMDDGPSRRDHFMKSGFASGRNFGNRDAGECNKRDNTST<br>MGGFGVGKSFGNRGFSNSRFEDGDSSGFWRESSNDCEDNPTRNRGFSKRGGYRDGNNSEASGPYRRGGRGSFRGCRGGFGLG<br>SPNNDLDPDECMQRTGGLFGSRRPVLSGTGNGDTSQSRSGSGSERGGYKGLNEEVITGSGKNSWKSEAEGGESMVSKGEELFTG<br>VVPILVELDGDVNGHKFSVSGEGEGDATYGKLTLKFICTTGKLPVPWPTLVTTFGYGLMCFARYPDHMKQHDFFKSAMPEGYVQ<br>ERTIFFKDDGNYKTRAEVKFEGDTLVNRIELKGIDFKEDGNILGHKLEYNYNSHNVYIMADKQKNGIKVNFKIRHNIEDGSVQLADH<br>YQQNTPIGDGPVLLPDNHYLSYQSKLSKDPNEKRDHMVLLEFVTAAGITFSTYIPGFSGSTRGNVFASVDTRKGKSTLNTAGFSSSQ<br>APNPVDDESWDGSGSGHHHHHH
Ddx4^{YFP1}Myc	MGDEDWEAEINPHMSSYVPIFEKDRYSGENGDNFNRTPASSSEMDDGPSRRDHFMKSGFASGRNFGNRDAGECNKRDNTST<br>MGGFGVGKSFGNRGFSNSRFEDGDSSGFWRESSNDCEDNPTRNRGFSKRGGYRDGNNSEASGPYRRGGRGSFRGCRGGFGLG<br>SPNNDLDPDECMQRTGGLFGSRRPVLSGTGNGDTSQSRSGSGSERGGYKGLNEEVITGSGKNSWKSEAEGGESMVSKGEELFTG<br>VVPILVELDGDVNGHKFSVSGEGEGDATYGKLTLKFICTTGKLPVPWPTLVTTFGYGLMCFARYPDHMKQHDFFKSAMPEGYVQ<br>ERTIFFKDDGNYKTRAEVKFEGDTLVNRIELKGIDFKEDGNILGHKLEYNYNSHNVYIMADKQKNGIKVNFKIRHNIEDGSVQLADH<br>YQQNTPIGDGPVLLPDNHYLSYQSKLSKDPNEKRDHMVLLEFVTAAGITFSTYIPGFSGSTRGNVFASVDTRKGKSTLNTAGFSSSQ<br>APNPVDDESWDGSGSGEQKLISEEDL
Ddx4^{YFP1}Flag	MGDEDWEAEINPHMSSYVPIFEKDRYSGENGDNFNRTPASSSEMDDGPSRRDHFMKSGFASGRNFGNRDAGECNKRDNTST<br>MGGFGVGKSFGNRGFSNSRFEDGDSSGFWRESSNDCEDNPTRNRGFSKRGGYRDGNNSEASGPYRRGGRGSFRGCRGGFGLG<br>SPNNDLDPDECMQRTGGLFGSRRPVLSGTGNGDTSQSRSGSGSERGGYKGLNEEVITGSGKNSWKSEAEGGESMVSKGEELFTG<br>VVPILVELDGDVNGHKFSVSGEGEGDATYGKLTLKFICTTGKLPVPWPTLVTTFGYGLMCFARYPDHMKQHDFFKSAMPEGYVQ<br>ERTIFFKDDGNYKTRAEVKFEGDTLVNRIELKGIDFKEDGNILGHKLEYNYNSHNVYIMADKQKNGIKVNFKIRHNIEDGSVQLADH<br>YQQNTPIGDGPVLLPDNHYLSYQSKLSKDPNEKRDHMVLLEFVTAAGITFSTYIPGFSGSTRGNVFASVDTRKGKSTLNTAGFSSSQ<br>APNPVDDESWDGSGSGDYKDDDDK
Ddx4^{YFP1}HA	MGDEDWEAEINPHMSSYVPIFEKDRYSGENGDNFNRTPASSSEMDDGPSRRDHFMKSGFASGRNFGNRDAGECNKRDNTST<br>MGGFGVGKSFGNRGFSNSRFEDGDSSGFWRESSNDCEDNPTRNRGFSKRGGYRDGNNSEASGPYRRGGRGSFRGCRGGFGLG<br>SPNNDLDPDECMQRTGGLFGSRRPVLSGTGNGDTSQSRSGSGSERGGYKGLNEEVITGSGKNSWKSEAEGGESMVSKGEELFTG<br>VVPILVELDGDVNGHKFSVSGEGEGDATYGKLTLKFICTTGKLPVPWPTLVTTFGYGLMCFARYPDHMKQHDFFKSAMPEGYVQ<br>ERTIFFKDDGNYKTRAEVKFEGDTLVNRIELKGIDFKEDGNILGHKLEYNYNSHNVYIMADKQKNGIKVNFKIRHNIEDGSVQLADH<br>YQQNTPIGDGPVLLPDNHYLSYQSKLSKDPNEKRDHMVLLEFVTAAGITFSTYIPGFSGSTRGNVFASVDTRKGKSTLNTAGFSSSQ<br>APNPVDDESWDGSGSGYPYDVPDYA

Ddx4^{YFP1}Strep	MGDEDWEAEINPHMSSYVPIFEKDRYSGENGDNFNRTPASSSEMDDGPSRRDHFMKSGFASGRNFGNRDAGECNKRDNTST<br>MGGFGVGKSFGNRGFSNSRFEDGDSSGFWRESSNDCEDNPTRNRGFSKRGGYRDGNNSEASGPYRRGGRGSFRGCRGGFGLG<br>SPNNDLDPDECMQRTGGLFGSRRPVLSGTGNGDTSQSRSGSGSERGGYKGLNEEVITGSGKNSWKSEAEGGESMVSKGEELFTG<br>VVPILVELDGDVNGHKFSVSGEGEGDATYGKLTLKFICTTGKLPVPWPTLVTTFGYGLMCFARYPDHMKQHDFFKSAMPEGYVQ<br>ERTIFFKDDGNYKTRAEVKFEGDTLVNRIELKGIDFKEDGNILGHKLEYNYNSHNVYIMADKQKNGIKVNFKIRHNIEDGSVQLADH<br>YQQNTPIGDGPVLLPDNHYLSYQSKLSKDPNEKRDHMVLLEFVTAAGITFSTYIPGFSGSTRGNVFASVDTRKGKSTLNTAGFSSSQ<br>APNPVDDESWDGSGSGWSHPQFEK
Ddx4^{YFP1}Xpress	MGDEDWEAEINPHMSSYVPIFEKDRYSGENGDNFNRTPASSSEMDDGPSRRDHFMKSGFASGRNFGNRDAGECNKRDNTST<br>MGGFGVGKSFGNRGFSNSRFEDGDSSGFWRESSNDCEDNPTRNRGFSKRGGYRDGNNSEASGPYRRGGRGSFRGCRGGFGLG<br>SPNNDLDPDECMQRTGGLFGSRRPVLSGTGNGDTSQSRSGSGSERGGYKGLNEEVITGSGKNSWKSEAEGGESMVSKGEELFTG<br>VVPILVELDGDVNGHKFSVSGEGEGDATYGKLTLKFICTTGKLPVPWPTLVTTFGYGLMCFARYPDHMKQHDFFKSAMPEGYVQ<br>ERTIFFKDDGNYKTRAEVKFEGDTLVNRIELKGIDFKEDGNILGHKLEYNYNSHNVYIMADKQKNGIKVNFKIRHNIEDGSVQLADH<br>YQQNTPIGDGPVLLPDNHYLSYQSKLSKDPNEKRDHMVLLEFVTAAGITFSTYIPGFSGSTRGNVFASVDTRKGKSTLNTAGFSSSQ<br>APNPVDDESWDGSGSGDLYDDDDK
Ddx4^{YFP1}Avi	MGDEDWEAEINPHMSSYVPIFEKDRYSGENGDNFNRTPASSSEMDDGPSRRDHFMKSGFASGRNFGNRDAGECNKRDNTST<br>MGGFGVGKSFGNRGFSNSRFEDGDSSGFWRESSNDCEDNPTRNRGFSKRGGYRDGNNSEASGPYRRGGRGSFRGCRGGFGLG<br>SPNNDLDPDECMQRTGGLFGSRRPVLSGTGNGDTSQSRSGSGSERGGYKGLNEEVITGSGKNSWKSEAEGGESMVSKGEELFTG<br>VVPILVELDGDVNGHKFSVSGEGEGDATYGKLTLKFICTTGKLPVPWPTLVTTFGYGLMCFARYPDHMKQHDFFKSAMPEGYVQ<br>ERTIFFKDDGNYKTRAEVKFEGDTLVNRIELKGIDFKEDGNILGHKLEYNYNSHNVYIMADKQKNGIKVNFKIRHNIEDGSVQLADH<br>YQQNTPIGDGPVLLPDNHYLSYQSKLSKDPNEKRDHMVLLEFVTAAGITFSTYIPGFSGSTRGNVFASVDTRKGKSTLNTAGFSSSQ<br>APNPVDDESWDGSGSGGLNDIFEAQKIEWHE
Ddx4^{YFP1}V5	MGDEDWEAEINPHMSSYVPIFEKDRYSGENGDNFNRTPASSSEMDDGPSRRDHFMKSGFASGRNFGNRDAGECNKRDNTST<br>MGGFGVGKSFGNRGFSNSRFEDGDSSGFWRESSNDCEDNPTRNRGFSKRGGYRDGNNSEASGPYRRGGRGSFRGCRGGFGLG<br>SPNNDLDPDECMQRTGGLFGSRRPVLSGTGNGDTSQSRSGSGSERGGYKGLNEEVITGSGKNSWKSEAEGGESMVSKGEELFTG<br>VVPILVELDGDVNGHKFSVSGEGEGDATYGKLTLKFICTTGKLPVPWPTLVTTFGYGLMCFARYPDHMKQHDFFKSAMPEGYVQ<br>ERTIFFKDDGNYKTRAEVKFEGDTLVNRIELKGIDFKEDGNILGHKLEYNYNSHNVYIMADKQKNGIKVNFKIRHNIEDGSVQLADH<br>YQQNTPIGDGPVLLPDNHYLSYQSKLSKDPNEKRDHMVLLEFVTAAGITFSTYIPGFSGSTRGNVFASVDTRKGKSTLNTAGFSSSQ<br>APNPVDDESWDGSGSGGKPIPNPLLGLDST
Ddx4^{YFP1}NLS	MGDEDWEAEINPHMSSYVPIFEKDRYSGENGDNFNRTPASSSEMDDGPSRRDHFMKSGFASGRNFGNRDAGECNKRDNTST<br>MGGFGVGKSFGNRGFSNSRFEDGDSSGFWRESSNDCEDNPTRNRGFSKRGGYRDGNNSEASGPYRRGGRGSFRGCRGGFGLG<br>SPNNDLDPDECMQRTGGLFGSRRPVLSGTGNGDTSQSRSGSGSERGGYKGLNEEVITGSGKNSWKSEAEGGESMVSKGEELFTG<br>VVPILVELDGDVNGHKFSVSGEGEGDATYGKLTLKFICTTGKLPVPWPTLVTTFGYGLMCFARYPDHMKQHDFFKSAMPEGYVQ<br>ERTIFFKDDGNYKTRAEVKFEGDTLVNRIELKGIDFKEDGNILGHKLEYNYNSHNVYIMADKQKNGIKVNFKIRHNIEDGSVQLADH<br>YQQNTPIGDGPVLLPDNHYLSYQSKLSKDPNEKRDHMVLLEFVTAAGITFSTYIPGFSGSTRGNVFASVDTRKGKSTLNTAGFSSSQ<br>APNPVDDESWDGSGSGPKKKRKV
Ddx4^{YFP1}Spot	MGDEDWEAEINPHMSSYVPIFEKDRYSGENGDNFNRTPASSSEMDDGPSRRDHFMKSGFASGRNFGNRDAGECNKRDNTST<br>MGGFGVGKSFGNRGFSNSRFEDGDSSGFWRESSNDCEDNPTRNRGFSKRGGYRDGNNSEASGPYRRGGRGSFRGCRGGFGLG<br>SPNNDLDPDECMQRTGGLFGSRRPVLSGTGNGDTSQSRSGSGSERGGYKGLNEEVITGSGKNSWKSEAEGGESMVSKGEELFTG<br>VVPILVELDGDVNGHKFSVSGEGEGDATYGKLTLKFICTTGKLPVPWPTLVTTFGYGLMCFARYPDHMKQHDFFKSAMPEGYVQ<br>ERTIFFKDDGNYKTRAEVKFEGDTLVNRIELKGIDFKEDGNILGHKLEYNYNSHNVYIMADKQKNGIKVNFKIRHNIEDGSVQLADH<br>YQQNTPIGDGPVLLPDNHYLSYQSKLSKDPNEKRDHMVLLEFVTAAGITFSTYIPGFSGSTRGNVFASVDTRKGKSTLNTAGFSSSQ<br>APNPVDDESWDGSGSGPDRVRAVSHWSS

Table S1. Amino acid sequences of proteins used in the paper. Residual cloning tags at the N-termini of recombinantly expressed and purified protein sequences (GAMGS) are underlined. Residue numbering indicated in the main text starts from the first non-underlined amino acid.

Protein name / amino acid numbers in construct	**Ddx4^{N1}/6-241 vs other sequences used in the study**	**Alignment length**	**% identity**	**% similarity**
>Ddx4^{N1}/1-241	Ddx4^{N1}/6-241	236	100.0	100.0
>Ddx3x^{N}/1-142	Ddx3x^{N}/6-142	237	12.2	23.6
>Ddx3x^{N}_FLAG/1-150	Ddx3x^{N}_FLAG/6-150	237	12.2	24.5
>Ddx3y^{N}/1-140	Ddx3y^{N}/6-140	237	10.6	22.8
>Vasa^{N}/1-197	Vasa^{N}/6-197	247	17.0	30.8
>Bel^{N}/1-257	Bel^{N}/6-257	288	15.6	32.3
>drDdx4^{N}/1-230	drDdx4^{N}/6-230	255	20.8	38.4
>Ddx4^{N0}_238/1-243	Ddx4^{N0}_238/6-243	238	99.2	99.2
>Ddx4^{N1}_236/1-241	Ddx4^{N1}_236/6-241	236	100.0	100.0
>Ddx4^{N2}_234/1-239	Ddx4^{N2}_234/6-239	236	99.2	99.2
>Ddx4^{N3}_231/1-236	Ddx4^{N3}_231/6-236	236	97.9	97.9
>Ddx4^{N4}_229/1-234	Ddx4^{N4}_229/6-234	236	97.0	97.0

Table S2. Amino acid sequence similarity and identity scores for Ddx4 and Ddx3 homologues, related to Figure 2. Amino acid groupings were: AVLIM, FYW, GSTCHNQ, P, KR, DE. https://www.bioinformatics.org/sms2/ident_sim.html, ref [5].

Parameter	Fitted value	±	$x0$	Mean	Stdev
ΔG_{nn}	-21.91	1.12	-22.45	-22.43	1.07
ΔG_{pp}	-27.02	3.64	-26.13	-25.79	3.23
ΔG_{pn}	16.22	0.93	17.25	16.98	0.90
$\Delta G_{FG/RG}$	55.82	7.40	53.62	51.66	7.24
Ca^{2+}	73.22	14.08	73.18	74.37	16.51
Mg^{2+}	105.21	18.56	108.71	112.59	29.69
Y^{3+}	5.47	1.99	5.83	4.83	2.36
K^{+}	487.27	103.39	503.24	2739.25	15083.85
Na^{+}	3763.19	1700.23	4207.47	231054.69	871979.16
Gu^{+}	173.06	21.10	175.53	180.93	34.66
$r0_{nn}$	67.52	3.67	63.63	63.71	3.78
$r0_{pp}$	54.44	9.32	56.04	56.04	8.44
$r0_{pn}$	91.46	5.95	84.23	84.48	6.10
$r0_{FG/RG}$	25.97	4.53	27.31	27.72	4.71
erval	46.53	2.10	48.36	48.30	1.81

Table S3. Stickers and spacers model parameters.

Supplementary references

1. Nott, T. J. *et al.* Phase Transition of a Disordered Nuage Protein Generates Environmentally Responsive Membraneless Organelles. *Mol. Cell* **57**, 936–947 (2015).
2. Brady, J. P. *et al.* Structural and hydrodynamic properties of an intrinsically disordered region of a germ cell-specific protein on phase separation. *Proc. Natl. Acad. Sci. U. S. A.* **114**, E8194–E8203 (2017).
3. Millard, P. S. *et al.* IDDomainSpotter: Compositional bias reveals domains in long disordered protein regions—Insights from transcription factors. *Protein Sci.* **29**, 169–183 (2020).
4. Tamiola, K., Acar, B. & Mulder, F. A. A. Sequence-specific random coil chemical shifts of intrinsically disordered proteins. *J. Am. Chem. Soc.* **132**, 18000–18003 (2010).
5. Stothard, P. The sequence manipulation suite: JavaScript programs for analyzing and formatting protein and DNA sequences. *Biotechniques* **28**, (2000).
